# Angle-based fluorescence thermometry with high sensitivity and high resolution

**DOI:** 10.1038/s41467-026-73430-4

**Published:** 2026-05-22

**Authors:** Xuanzheng Zhou, Kang Xu, Zekai Li, Yuxuan Dong, Jinghui Chao, Yufei Zhai, Ying Jin, Shaolin Xu, Min Wang

**Affiliations:** 1 School of Microelectronics, Southern University of Science and Technology, Shenzhen, PR China; 2 Department of Mechanical and Energy Engineering, Southern University of Science and Technology, Shenzhen, PR China; 3 State Key Laboratory of Quantum Functional Materials, Southern University of Science and Technology, Shenzhen, PR China

**Keywords:** Nanophotonics and plasmonics, Fluorescence resonance energy transfer, Metamaterials

## Abstract

Fluorescence-based thermometry offers non-contact, high-resolution temperature sensing, yet current methods face challenges in simultaneously achieving high sensitivity, stability and insensitivity to non-thermal perturbations. Here, a sensing paradigm is introduced that translates temperature variations into detectable shifts in the fluorescence emission angle via grating-modulated fluorescence emission. In a prototype device featuring a gold grating coupled with [Ru(phen)₃]Cl₂ fluorophores, an absolute sensitivity of 0.58 °/°C and a relative sensitivity of 81.34 %/°C at 79.36 °C are achieved, with a temperature resolution down to 0.01 °C. Unlike intensity-based methods, this angle-resolved strategy effectively isolates the thermal signal from excitation power fluctuations and environmental interferences and demonstrates robustness across broad humidity ranges (10–89% RH). Sustained operational stability is validated over 12-hour battery cycling, while real-time monitoring of a smartphone CPU enables early-warning capabilities against overheating. This promising and customizable approach establishes a pathway for thermal management in advanced engineering systems.

## Abstract (web summary)

A plasmonic grating-based thermometer translates temperature into fluorescence emission angle shifts. This non-contact method achieves 0.01 °C resolution and effectively isolates thermal signals from environmental interferences.

## Introduction

Precise temperature monitoring is essential across diverse fields, including quantum computing, nanoelectronics, aerospace engineering, and advanced biomedical diagnostics^1,2^. Its importance stems from enabling effective thermal management, optimizing system performance, and ensuring device reliability^3^. As microelectronic, electrochemical, and biomedical systems continue toward miniaturization and higher integration densities, localized overheating emerges as a critical factor impacting performance, stability, and operational lifespan. Conventional thermometry techniques, such as thermocouples and infrared sensors, often fail to meet the stringent requirements for resolution, measurement accuracy, and response speed demanded by these advanced applications^4^. Consequently, optical thermometry, which leverages the temperature-dependent optical response of materials, has garnered significant attention due to its potential for high-resolution, rapid, and non-contact sensing.

Among various optical thermometry strategies, fluorescence-based thermometry offers advantages in miniaturization, ease of integration, and superior spatial resolution^1^. This approach relies on changes in key optical parameters of temperature-responsive fluorescent materials to sense temperature. Mainstream fluorescence thermometry mechanisms include fluorescence intensity^5–7^, fluorescence intensity ratio (FIR)^8–11^, fluorescence lifetime^12^, and emission peak position^13,14^. The fluorescence intensity variation method relies on thermal quenching, in which the absolute emission intensity decreases as the temperature rises. This approach is constrained by its direct dependence on signal magnitude, rendering it sensitive to non-thermal perturbations such as excitation-power fluctuations, fluorophore concentration changes, and environmental factors (e.g., pH and viscosity). Unlike genuine thermal responses, these perturbations introduce signal artifacts that are often indistinguishable from temperature variations. To address these limitations, FIR-based methods have been developed. By monitoring the intensity ratio of two emission bands or polarization states from the same probe, self-referencing calibration effectively eliminates errors caused by excitation fluctuations and concentration variations, thereby improving sensing stability. Material engineering strategies such as specific ion co-doping (for example, Pr³⁺/Yb³⁺ co-doped oxides) to optimize energy-level structures or exploiting phase transition behavior in defined temperature windows (for example, LiYO₂:Ln systems) are often employed to enhance the temperature sensitivity of the intensity ratio or to extend its effective range^15,16^. Despite reported sensitivity improvements reaching 11.2% K^−1^ and 26.1% K^−1^ near phase transitions, these methods also face challenges, including complex synthesis and calibration, limited effectiveness within narrow temperature windows, and inherent trade-offs between sensitivity and emission intensity. Another important thermometric mechanism relies on the temperature-dependent fluorescence lifetime. As an intrinsic molecular property, fluorescence lifetime is largely insensitive to variations in fluorophore concentration and excitation intensity, conferring excellent immunity to external perturbations^17^. However, precise lifetime measurements typically require complex and costly instrumentation (e.g., time-correlated single-photon counting systems), and the limited dynamic range of lifetime changes in some materials constrains their applicability in ultra-high-sensitivity contexts. Finally, while monitoring the shift in emission peak position offers a direct reflection of energy-level alterations, this approach typically suffers from low sensitivity and requires high spectral resolution.

In recent years, integrating plasmonic effects from metallic nanostructures to enhance local electromagnetic fields with fluorescence thermometry has emerged as a promising signal-amplification strategy^18,19^. Such near-field enhancement can increase both excitation and emission efficiency of fluorophores, thereby amplifying the overall fluorescence signal. Researchers have demonstrated progress in improving detection sensitivity and accelerating response time by combining fluorescent materials with plasmonic nanostructures such as nanoparticles, nanopore arrays, and gratings. It should be emphasized that existing plasmon-enhanced fluorescence thermometry studies still rely on the traditional sensing parameters of emission intensity, intensity ratio, peak shift, or polarization^20,21^. Plasmonic structures in these cases serve primarily as signal amplifiers without altering the fundamental sensing dimension. Consequently, despite significant advances, simultaneous achievement of high sensitivity, sub-micrometer resolution, stability, and compatibility with complex integrated environments remains challenging, prompting exploration of fundamentally different fluorescence thermometry mechanisms.

To overcome the inherent limitations of conventional fluorescence-based sensors, a temperature-sensing paradigm based on grating-induced fluorescence emission angle modulation is developed in this work. The core mechanism relies on a cascaded amplification chain wherein thermal perturbations are sequentially transduced through molecular thermochromism, resonant refractive index dispersion, and plasmonic momentum matching. This process efficiently converts subtle molecular-level adjustments into substantial, detectable shifts in the fluorescence emission angle. Following this principle, a sensor was designed and optimized, demonstrating an absolute sensitivity of 0.58°/°C and a resolution of 0.01 °C. Beyond high sensitivity, the paradigm is explicitly designed to ensure immunity to non-thermal perturbations, a capability validated through rigorous stability tests. The sensor’s compatibility with femtosecond laser fabrication further allows for flexible customization on complex surfaces^22^, paving the way for future array integration. This work not only offers a powerful tool for micro-scale thermal characterization but also provides a comprehensive physical framework for utilizing molecule-plasmon coupling in precision metrology.

## Results

### *Sensing principle*

**Fig. 1:**
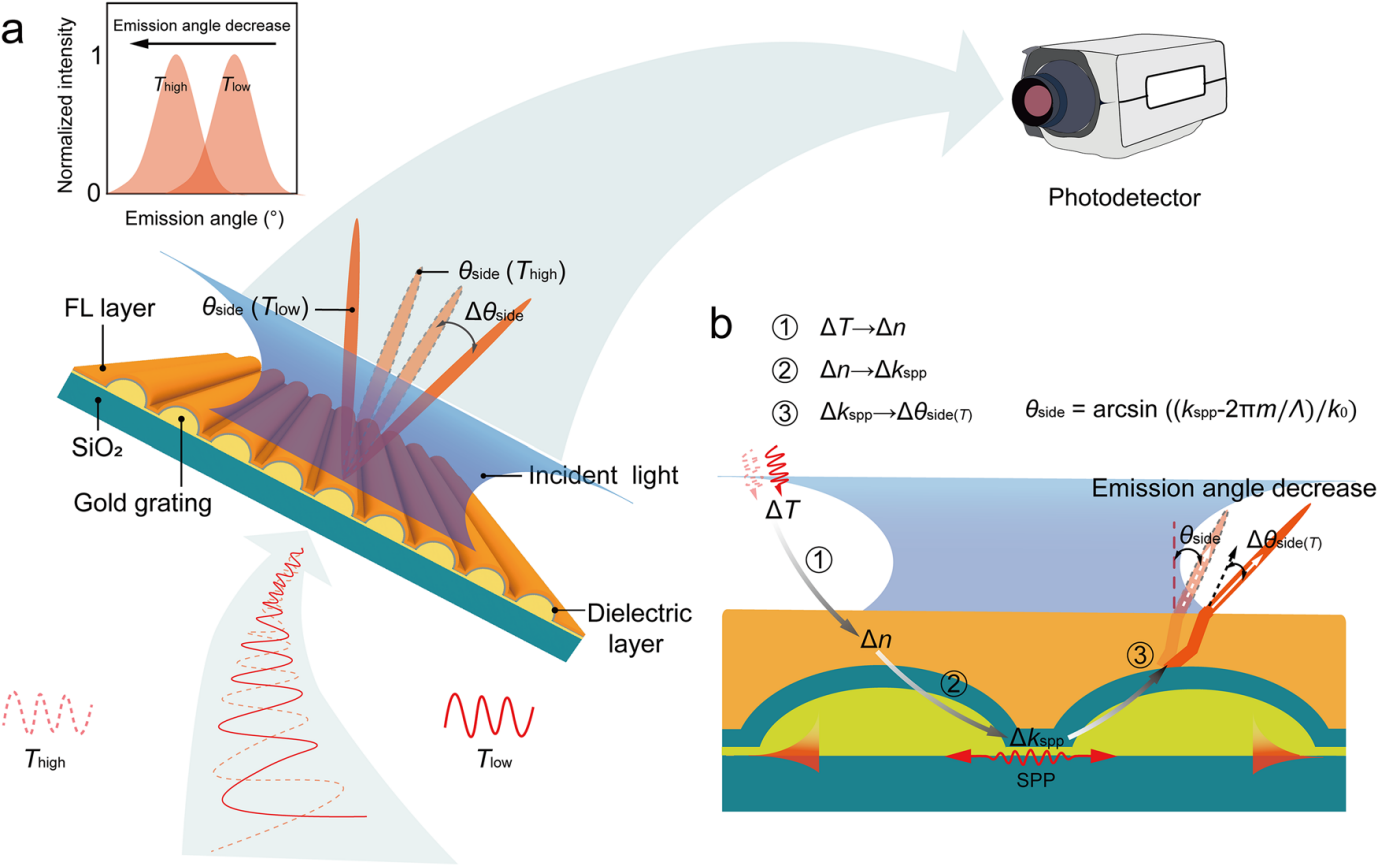
Schematic of the angle-based fluorescence thermometry principle and physical mechanism. **a** Macro-scale illustration of the sensor structure and far-field photodetector demonstrating the core sensing phenomenon where a specific surface plasmon polariton (SPP) coupled secondary emission angle (${\theta }_{{{{\rm{side}}}}}$) shifts from a large angle at low temperature (Low *T*) to a small angle at high temperature (High *T*). The top left inset shows the ideal signal paradigm deriving sensing information from the peak angular position with intensity normalized to unity to demonstrate independence from fluctuations. Bottom left inset illustrates external thermal states, where High *T* acts as the heat source, causing the angular shift relative to Low *T*. **b** Micro-scale mechanism of signal transduction involving a three-step physical chain where ① an input thermal change ($\Delta T$) induces a refractive index change ($\Delta n$) in the fluorescent (FL) layer, ② $\Delta n$ modulates the SPP wavevector ($\Delta {k}_{{{{\rm{SPP}}}}}$) at the metal-dielectric interface, and ③ $\Delta {k}_{{{{\rm{SPP}}}}}$ converts into an observable far-field angular shift ($\Delta {\theta }_{{{{\rm{side}}}}}$) governed by the grating coupling equation.

The temperature sensing paradigm proposed in this work relies on a core physical mechanism that translates temperature changes into a precisely readable shift in the fluorescence emission angle, as illustrated in Fig. 1. This process, detailed in Fig. 1b, involves a clear, three-step physical chain: Δ*T* → Δ*n* → Δ*k*_SPP _→ Δ*θ*_side_. A physical difference underlies this mechanism. Conventional metallic gratings exhibit multiple diffraction orders. The primary diffraction angle (i.e., the first-order Bragg peak) is governed principally by the grating’s physical period (Λ). As the thermal expansion of gold is negligible, this primary peak is effectively temperature-insensitive. In contrast, the secondary emission angle (*θ*_side_) is governed by both the grating period and the surface plasmon polariton (SPP) wavevector (*k*_SPP_). This paradigm utilizes the *T*-sensitive secondary angle as the core sensing signal.

Specifically, an input thermal change (Δ*T*) induces changes in the local structure of the dichlorotris(1,10-phenanthroline)ruthenium(II) hydrate ([Ru(phen)_3_]Cl_2_) molecules or their interactions with the environment. At the macroscopic level, this manifests as a distinct decrease in the effective refractive index (Δ*n*) of the fluorescent thin film (Fig. 1b, step ①). Subsequently, this change in Δ*n* modulates the resonance state of the underlying gold grating. SPPs are coupled oscillations at the metal-dielectric interface whose resonance condition is sensitive to the surrounding refractive index (*n*). According to the SPP dispersion relation, a decrease in *n* directly leads to a decrease in the *k*_SPP_ (Fig. 1b, step ②). Finally, the change in the SPP resonance state is recorded. As the signal corresponds to the secondary diffraction angle, *θ*_side_(*T*), whose position is governed by the grating coupling equation, ${k}_{0}\sin {\theta }_{{{\mathrm{side}}}}=\,{k}_{{{\mathrm{SPP}}}}-\frac{2{{{\rm{\pi }}}}m}{\varLambda }$ (Fig. 1b, formula). According to this relationship, a decrease in the *k*_SPP_ necessitates a corresponding decrease in sin *θ*_side_, causing this specific secondary angle to shift toward a smaller value (Fig. 1b, step ③). This establishes the complete signal transduction chain. Notably, while temperature increases are often accompanied by thermal quenching (a decrease in fluorescence intensity), this remains a co-existing phenomenon. This paradigm is independent of such intensity instabilities. As illustrated in the ideal signal schematic (Fig. 1a, top-left inset), the sensing information is derived from the normalized peak position (angle) rather than the peak amplitude (intensity). This clear physical mechanism, based on the modulation of the secondary diffraction angle, forms the foundation of the high-sensitivity, angle-based thermometry demonstrated in this work.

### *Platform selection and operating principle of angle-based transduction*

**Fig. 2:**
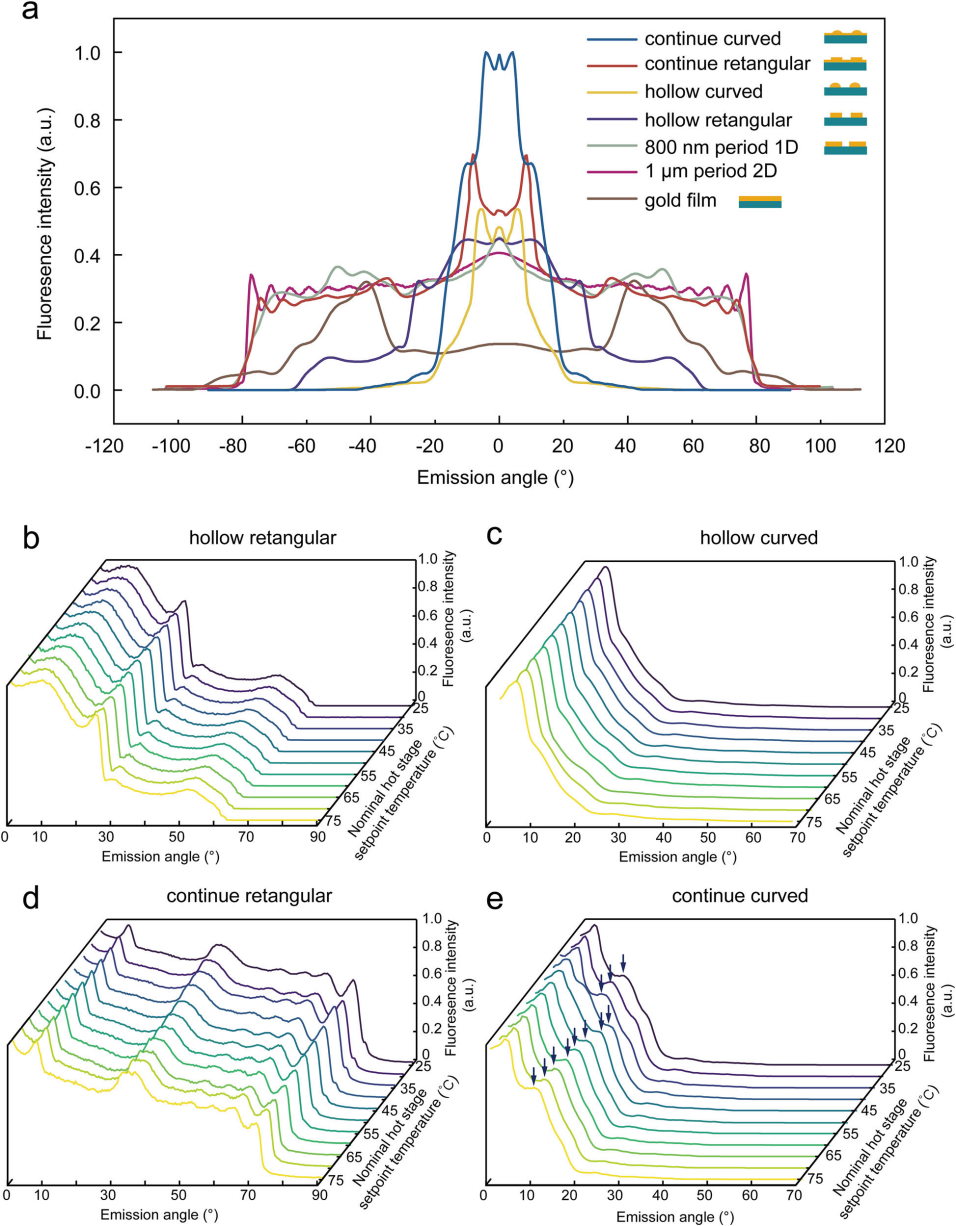
SPP platform selection and physical basis for angle-based transduction. **a** Fluorescence emission angle distributions at room temperature for seven gold morphologies, each with an initial thickness set to 50 nm, showing distinct emission peaks. **b–e** To isolate the angular shift from the inherent thermal quenching of fluorescence intensity, the emission spectra at different temperatures are normalized to unity intensity. **b** Temperature-dependent angular response of hollow rectangular gold gratings showing negligible shift of first- and second-order diffraction peaks. **c** Temperature-dependent angular response of hollow curved gold gratings confirming absence of measurable diffraction-angle shifts. **d** Diffraction intensity profiles for continuous rectangular gold gratings revealing multiple second-order peaks without temperature sensitivity. **e** Temperature response of the continuous curved grating, highlighting a clear shift of the *θ*_side_ with temperature (shift direction indicated by black arrows). Note: All temperatures in this figure represent the nominal hot stage setpoint temperature used for qualitative platform screening.

A step towards an efficient angle-based sensor (specifically for the platform transduction gain) involves selecting an SPP physical platform sensitive to interfacial *n* changes. Therefore, the thermo-responsive optical properties of different grating morphologies were systematically investigated to identify an optimal design (Fig. 2). Evaluation relied on geometric parameters, diffraction patterns, and temperature sensitivity. Three criteria were essential for a reliable platform, including a well-defined emission angle, significant angle-temperature dependence, and a limited number (≤3) of diffraction peaks to minimize detector bias and ensure clear signal discrimination in practical applications.

Figure 2a provides a comparative analysis of seven gold nanostructures at room temperature. These initial structures had a thickness of 50 nm and lacked a dielectric layer. All comparative screening experiments in this section utilize the nominal hot stage setpoint temperature as the control variable to qualitatively identify the most responsive structure. The experimental setup shown in Fig. S1 was used to evaluate the temperature response of various gold nanostructures. The experimental evaluation (Fig. S2) first eliminated unsuitable configurations like gold films and mismatched gratings. A conventional gold film exhibited only a single, primary diffraction peak (~45°) lacking temperature dependence, rendering it unsuitable for angle-based thermometry (Fig. S2a). Similarly, two-dimensional (2D) disordered structures and one-dimensional (1D) gratings with ~800 nm periods failed to produce distinct fluorescence emission angles or observable temperature responses (Fig. S2b, c). Figure S3 shows scanning electron microscopy (SEM) images of 2D structures and ~800 nm period 1D structures. Subsequent simulations indicated an optimal period of approximately 500 nm for 1D gratings, which was adopted for further morphological comparisons (Fig. S4).

Subsequently, four specific grating structures (500 nm period) were evaluated. Hollow gratings, both rectangular (Fig. 2b) and curved (Fig. 2c), did not exhibit measurable temperature-induced angular shifts, indicating that these 50 nm hollow structures lack sufficient temperature responsiveness. To enhance the response, continuous gratings were formed. The continuous rectangular grating (Fig. 2d) produced too many peaks (six in total) and lacked sensitivity. In contrast, 1D continuous curved gold grating (Fig. 2e) displayed three clear diffraction angles. Its ${\theta }_{{{{\rm{side}}}}}$ exhibited a distinct angular shift with increasing temperature. This finding identified the continuous curved gold grating as the most promising base platform for angle-based transduction.

The differential temperature response observed between the primary and secondary diffraction peaks originates from the fundamental distinction in their physical governing mechanisms. The primary (first-order) diffraction peak is governed principally by the standard geometric Bragg condition. Derived from the fundamental grating equation $\varLambda \sin {\theta }_{{{{\rm{p}}}}}=m\lambda$ by introducing the free-space wavevector (${k}_{0}=2{{{\rm{\pi }}}}/\lambda$), this condition is expressed as:

1 $${k}_{0}\sin {\theta }_{{{{\rm{p}}}}}=\frac{2{{{\rm{\pi }}}}m}{\varLambda },\,m=\pm 1,\pm 2,\ldots$$

where *θ*_p_ is the primary diffraction angle, and Λ is the grating period. As temperature variations induce negligible changes in the geometric period Λ due to the low thermal expansion coefficient of gold (∼14×10^−6^ K^−1^), the primary peak position remains effectively constant over typical experimental temperature ranges.

In contrast, the secondary peaks are the radiative signatures of SPPs. The angular position of these SPP-coupled peaks (*θ*_side_) is dictated by the momentum matching condition (Eq. 2):

2 $${k}_{{{{\rm{SPP}}}}}={k}_{0}\sin {\theta }_{{{{\rm{side}}}}}+\frac{2{{{\rm{\pi }}}}m}{\varLambda }$$

Critically, unlike the primary peak, this condition depends explicitly on the *k*_SPP_, which is sensitive to the dielectric environment as described by the dispersion relation (Eq. 3):

3 $${k}_{{{{\rm{SPP}}}}}={k}_{0}\sqrt{\frac{{\varepsilon }_{{{{\rm{m}}}}}\cdot {\varepsilon }_{{{{\rm{d}}}}}}{{\varepsilon }_{{{{\rm{m}}}}}+{\varepsilon }_{{{{\rm{d}}}}}}}$$

Here, *ε*_m_ and *ε*_d_ (=*n*^2^) represent the permittivity of the metal and the surrounding medium, respectively. While temperature fluctuations affect both terms, the variation in *ε*_d_(*T*), driven by the fluorescent layer, is the primary factor altering SPP characteristics. As the temperature rises, the thermally induced decrease in *ε*_d_ directly reduces *k*_SPP_. Consequently, to satisfy the momentum matching condition in Eq. 3 under a constant Λ, the *θ*_side_ (for a specific *m*) must shift dynamically to smaller angles. This physical dichotomy, in which the primary peak is anchored by geometry while the secondary peak is modulated by material properties, provides a robust physical basis for the observed high-sensitivity angle shift.

To elucidate the difference in temperature responsiveness between continuous curved and continuous rectangular gratings, finite-difference time-domain (FDTD) simulations (Fig. S5) reveal that the normalized near-field electric field magnitude associated with the secondary diffraction mode is substantially higher for the curved grating (0.332) compared to the rectangular grating (0.083). Furthermore, the curved grating exhibited a more uniform distribution of surface hotspots with a higher average field magnitude (0.743), whereas hotspots in the rectangular structure were weaker (0.198) and primarily confined to the corners. This enhanced local field coupling efficiency in the curved geometry implies that its SPP resonance is more sensitive to subtle, temperature-induced changes in the dielectric environment (i.e., *n*). Consequently, the intensified local electric field more significantly modulates the interaction between the SPP mode and the fluorescent material. This allows the material’s intrinsic thermo-optic response (i.e., d*ε*_d_/d*T*) to be more effectively amplified and transduced into observable angular signal variations. This theoretical difference is corroborated by the experimental data, where the continuous rectangular structure showed a negligible angular shift (Fig. 2d), while the continuous curved grating achieved a measurable temperature-dependent shift (Fig. 2e, exhibiting shifts potentially up to ~10° across a given temperature range). Evidently, although the continuous curved grating shows promise, its sensitivity in this initial state (50 nm thickness, no dielectric layer) remains low. This clearly indicates that the system requires further optimization to enhance its sensitivity and fully realize its potential as a high-performance sensor.

### *FDTD-guided optimization enhances field coupling and transduction*

**Fig. 3:**
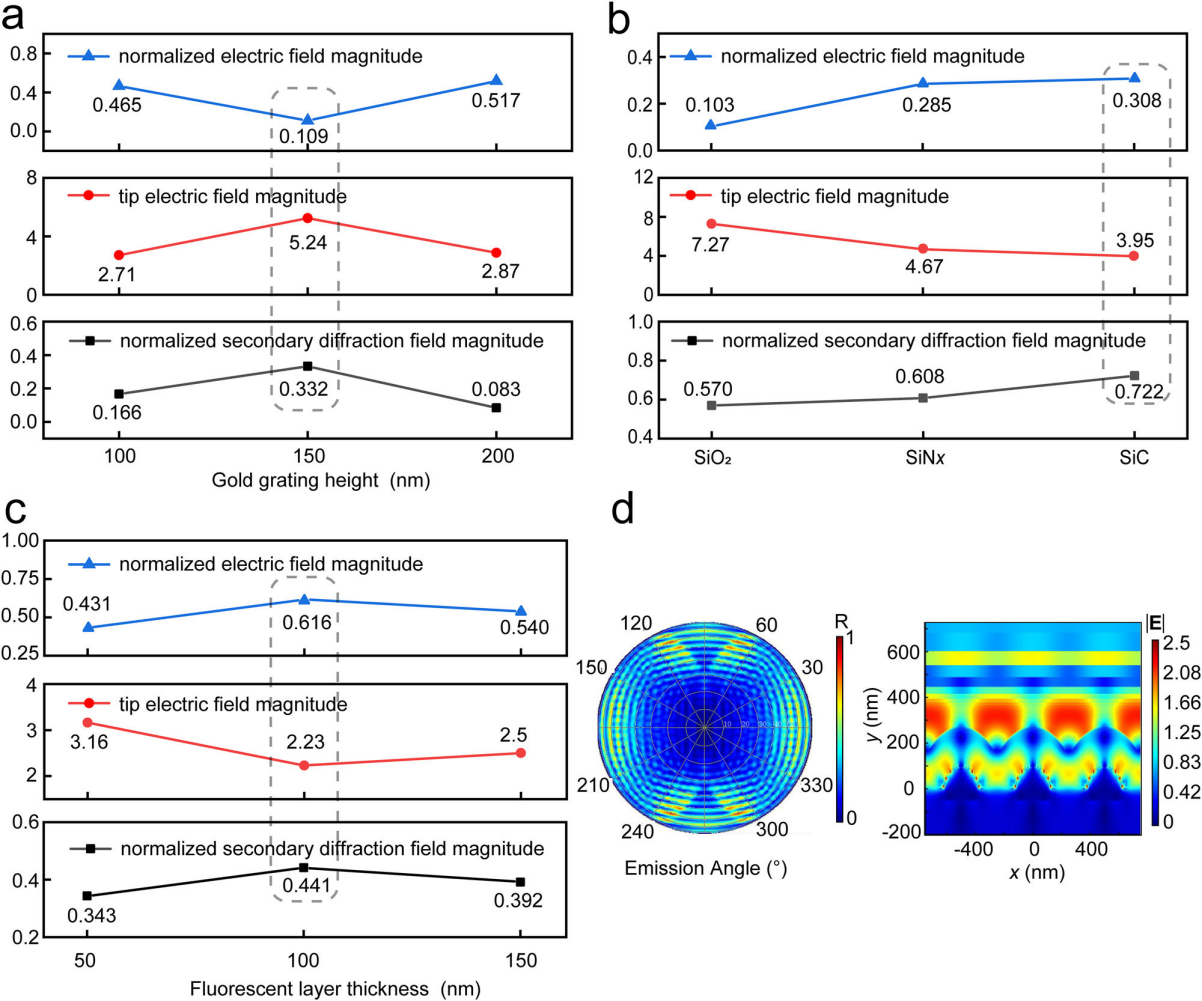
Key optimization steps for the angle-based temperature sensor via finite-difference time-domain (FDTD). **a** Optimizing gold grating thickness. **b** Comparing dielectric coatings. **c** Tuning fluorescent layer thickness. **d** Far-field diffraction angle map (left) and near-field electric field magnitude distribution (right) for the final structure comprising a 150 nm gold grating, 20 nm SiC layer, and 100 nm fluorescent layer. Gray dashed boxes in (**a**), (**b**), and (**c**) highlight the optimal parameter values. Data points represent deterministic values from numerical simulations and therefore do not include error bars. Spatial distributions in the color maps and normalized field magnitudes are normalized to their respective global maxima, yielding a dimensionless scale of 0–1 to visualize field uniformity. Peak values cited in the main text correspond to the raw absolute simulation magnitudes. These values represent the local field enhancement factor relative to a unit-amplitude incident source.

To maximize the efficiency of the plasmonic transduction sequence (specifically the conversion of Δ*n* to Δ*θ*_side_ illustrated in Fig. 1), key parameters were optimized via FDTD simulations (Fig. 3). Precise control over the gold layer thickness, the choice of dielectric material, and the fluorescent layer thickness enables simultaneous adjustment of the diffraction angle and near-field electric field magnitude.

First, the influence of the gold grating thickness on the far-field diffraction angle and near-field electric field magnitude was investigated via FDTD simulations (Figs. 3a and S6). An optimal balance was identified at a thickness of 150 nm. While this configuration yielded a normalized near-field electric field magnitude of 0.109 representing the average distribution, the localized maximum electric field magnitude at the tip increased to 5.24, and the normalized field magnitude within the secondary diffraction angle range (20°−50°) was 0.332. Furthermore, this thickness provided the most uniform surface hotspot distribution; deviations towards thinner or thicker gratings degraded this uniformity. For comparison, reducing the thickness to 100 nm increased the normalized near-field magnitude to 0.465 but decreased the tip electric field magnitude and normalized secondary diffraction angle field magnitude to 2.71 and 0.166, respectively. Conversely, increasing the thickness to 200 nm resulted in a normalized near-field magnitude of 0.517, while the tip field magnitude and normalized secondary diffraction angle field magnitude further decreased to 2.87 and 0.083, respectively.

Subsequently, a 20 nm dielectric layer was introduced on the optimized 150 nm gold grating to mitigate fluorescence quenching resulting from direct contact between the metal and fluorescent molecules. The effects of three dielectric materials, namely SiO₂, SiN*ₓ*, and SiC, on the system’s electric field magnitude and diffraction angle were compared (Figs. 3b and S7). SiN*ₓ* (yielding a ~60° angle) and SiC (~55°) demonstrated significantly larger secondary diffraction angles compared to SiO₂ (~30°). Critically, the SiC-based system exhibited the highest normalized near-field electric field magnitude (0.308) and normalized secondary diffraction angle field magnitude (0.722). Using SiN*ₓ* resulted in slightly lower magnitudes (0.285 and 0.608, respectively), whereas the SiO₂ system showed the weakest performance (0.103 and 0.571, respectively). Based on these findings, SiC was selected as the optimal dielectric material.

Finally, a fluorescent layer was spin-coated onto the SiC-modified structure to enable temperature detection, translating refractive index changes into observable diffraction angle shifts (Figs. 3c and S8). The thickness of this layer was optimized to maximize sensitivity. A thickness of 100 nm was determined to be optimal, maximizing the normalized near-field electric field magnitude (0.616) and positioning the secondary diffraction angle at approximately 35°, considered an ideal window for detection. This window facilitates efficient signal collection while maintaining measurable angular shifts; excessively large angles can hinder collection, while smaller angles may limit the discernible range. This optimal 100 nm thickness also corresponded to the strongest normalized field magnitude within the secondary diffraction angle (0.441). In contrast, fluorescent layer thicknesses of 50 nm and 150 nm resulted in reduced normalized near-field magnitudes (0.431 and 0.540, respectively) and lower normalized secondary diffraction angle field strengths (0.343 and 0.392, respectively). This performance variation is attributed to the spatial mismatch between the fluorescent molecule radiating dipoles and the SPP modes. Following the selection procedure above, Fig. 3d presents the far-field diffraction angle distribution and the near-field electric field magnitude distribution for the system comprising a 150 nm gold grating layer, a 20 nm SiC layer, and a 100 nm fluorescent layer.

### *Sensor calibration and mechanistic deconstruction of sensitivity*

Following optimization, the sensor’s thermal response was quantitatively characterized. In-situ calibration was performed to establish the precise relationship between the ${\theta }_{{{{\rm{side}}}}}$ and the actual sample surface temperature (*T*_actual_). Due to significant thermal resistance in the 1-mm-thick quartz substrate, the hot stage setpoint temperature does not accurately represent the sample temperature. Therefore, the angular response was calibrated directly against a co-located platinum resistance thermometer (Pt RTD, see Supplementary Note 1).

**Fig. 4:**
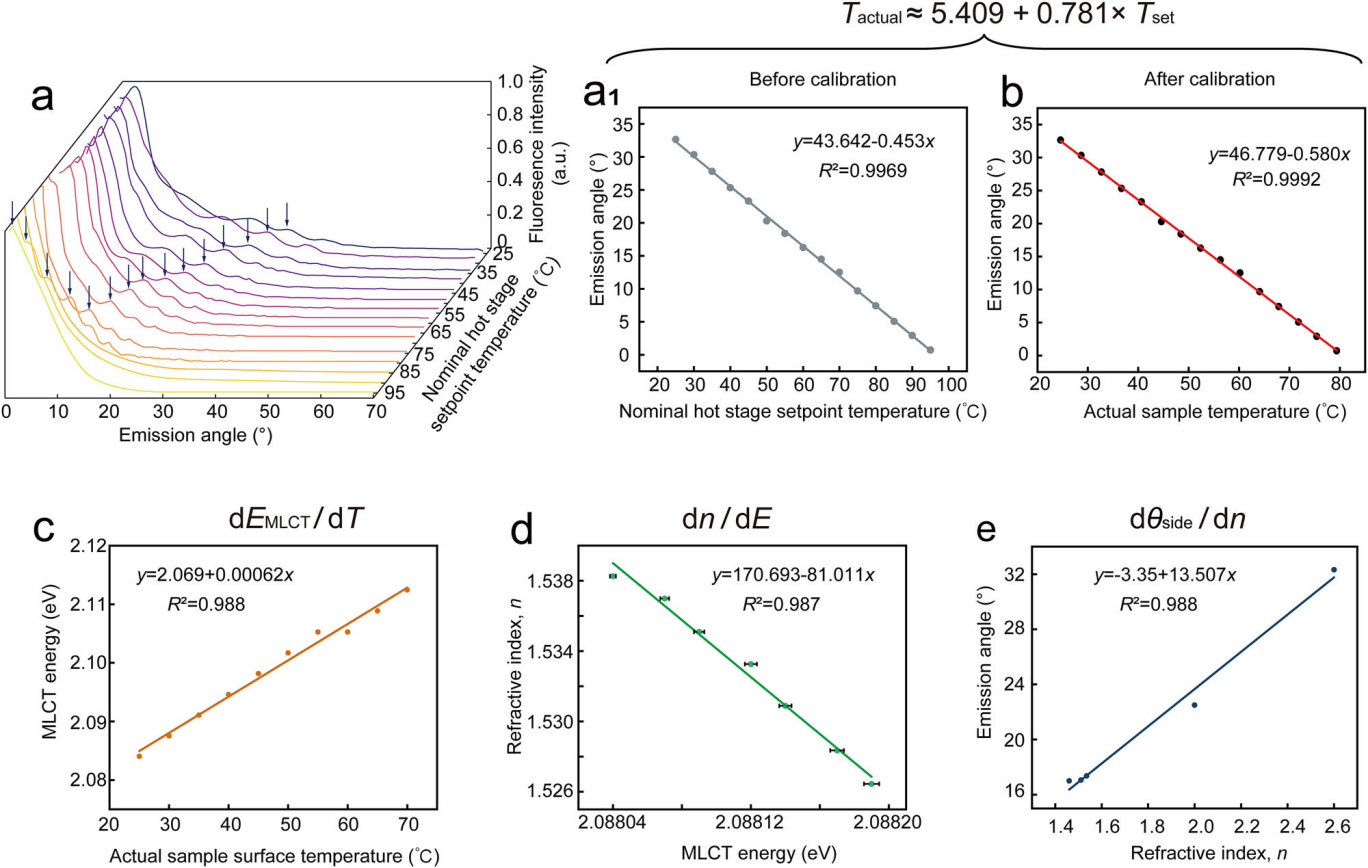
Sensor performance calibration and mechanistic validation via multi-stage deconstruction. **a** Angle-resolved fluorescence spectra recorded at nominal hot stage setpoints (*T*_set_) ranging from 25 °C to 95 °C showing that the *θ*_side_ indicated by black arrows shifts towards smaller angles as the temperature increases. **a₁** Dependence of ${\theta }_{{{{\rm{side}}}}}$ on the nominal setpoint temperature, where the relationship between the actual sample temperature (*T*_actual_) and the setpoint is quantified as *T*_actual_ = 5.409 + 0.781 × *T*_set,_ reflecting the thermal resistance of the substrate. **b** Sensor calibration curve plotting the extracted *θ*_side_ against *T*_actual_ measured in-situ via a calibrated platinum resistance thermometer (Pt RTD). This represents the final calibration derived from the comprehensive thermal characterization detailed in Fig. S9 using averaged heating and cooling measurements to eliminate thermal hysteresis artifacts. Linear fit (*R*² > 0.999) yields an experimental absolute sensitivity (*S*_abs_) of 0.580°/°C. **c–e** Quantitative deconstruction of the sensitivity into three constituent physical mechanisms (*S*_abs_ = d*E*/d*T* × d*n*/d*E* × d*θ*_side_/d*n*). **c** Step 1 (molecular thermochromism) where the metal-to-ligand charge transfer (MLCT) excited-state energy of dichlorotris(1,10-phenanthroline)ruthenium(II) hydrate ([Ru(phen)₃]Cl₂) exhibits a linear blueshift of 0.00062 eV/°C with *T*_actual_. **d** Step 2 (resonant dispersion) showing a linear correlation between the macroscopic refractive index (*n*) a*n*d the molecular energy (*E*_MLCT_), yielding a slope of 81.011 refractive index units (RIU)/eV. **e** St**e**p 3 (plasmonic momentum matching), where the grating structure transduces refractive index (*n*) changes into large angular (*θ*_side_) shifts with a measured gain of 13.507°/RIU. The product of these three components (*S*_calculated_ ≈ 0.678°/°C) quantitatively validates the direct measurement in **b**. Data points in (**a**)–(**c**) represent deterministic peak positions extracted from single continuous temperature-ramping measurements utilizing internal instrumental accumulations. Data points in (**d**) and (**e**) represent the mean of three independent measurements (*n* = 3), with error bars denoting the standard deviation (SD).

Figure 4a provides a qualitative overview of the angle-resolved spectra recorded at nominal setpoints from 25 °C to 95 °C, illustrating the sensing principle where the ${\theta }_{{{{\rm{side}}}}}$ shifts towards smaller angles as temperature increases (indicated by black arrows). The dependence of *θ*_side_ on the nominal setpoint is shown in Fig. 4a₁. The precise calibration curve is established in Fig. 4b (and detailed in Fig. S9). This nominal setpoint correlates to the *T*_actual_ via the linear relationship *T*_actual_ = 5.409 + 0.781 × *T*_set_, quantifying the significant thermal resistance of the substrate. The extracted *θ*_side_ is plotted against the *T*_actual_, demonstrating a linear relationship, *θ*_side_ = −0.58 × *T*_actual_ + 46.779 (*R*² = 0.9992). This corresponds to a high absolute sensitivity (*S*_abs_ = |d*θ*_side_/d*T*_actual_|) of 0.58°/°C. All temperatures reported hereafter refer to this calibrated, actual temperature. To validate this thermal characterization, the internal temperature uniformity within the sensor’s nano-scale functional layers was evaluated via finite-element simulation (Fig. S10). The model reveals a negligible vertical temperature gradient (<1 mK) between the plasmonic interface and the fluorescent layer surface, confirming that despite the macroscopic thermal resistance of the substrate, the sensing volume remains effectively isothermal.

To deconstruct the origin of this high sensitivity and validate the mechanism proposed in Fig. 1, the total angular response was deconstructed into a chain of three governing physical processes. Mathematically, the total sensitivity (*S*_abs_) is expressed via the chain rule as the product of three partial derivatives, each rooted in a fundamental physical interaction:

4 $${S}_{{\rm{abs}}}=\left|\frac{{{\rm{d}}}{\theta }_{{\rm{side}}}}{{\rm{d}}T}\right|={\left|\frac{{{\rm{d}}}{E}_{{{\rm{MLCT}}}}}{{{\rm{d}}}T}\right|}_{{{\mbox{Step}}}\,1} \times {\left|\frac{{{\rm{d}}}n}{{{\rm{d}}}{E}_{{{\rm{MLCT}}}}}\right|}_{{{\mbox{Step}}}\,2} \times {\left|\frac{{{\rm{d}}}{\theta }_{{{\rm{side}}}}}{{{\rm{d}}}n}\right|}_{{{\mbox{Step}}}\,3}$$

Here, the three terms correspond to the molecular thermochromic shift (d*E*_MLCT_/d*T*), the material’s resonant dispersion (d*n*/d*E*_MLCT_), and the platform’s plasmonic transduction gain (d*θ*_side_/d*n*), respectively. A rigorous theoretical derivation of these constituent mechanisms and their mathematical expressions is provided in Supplementary Note 1.

The transduction sequence initiates at the molecular level (Step 1), characterized by the thermodynamic modulation of the fluorophore’s electronic states. As shown in Fig. 4c (with raw spectral data provided in Fig. S11a), temperature-controlled spectroscopy reveals that the Metal-to-Ligand Charge Transfer (MLCT) emission peak of [Ru(phen)₃]Cl₂ exhibits a linear blueshift with temperature. Predicated on the Onsager reaction field theory^23,24^ (see Supplementary Note 1 for the polarity function derivation), this thermochromic shift arises from the thermal expansion of the host matrix, which reduces the local density and dielectric constant, thereby weakening the reaction field stabilization of the dipolar excited state. This process yields a reproducible energy shift (d*E*_MLCT_/d*T*) of 0.00062 eV/°C.

This variation in molecular energy levels dictates the macroscopic optical properties (Step 2), governed by the fundamental causality principle of optics. According to the Kramers–Kronig relations^25,26^ (detailed in Supplementary Note 1), a spectral shift in the absorption or emission band inherently necessitates a modification in the real part of the refractive index in the transparency region. To empirically quantify this link, spectroscopic ellipsometry was first employed to characterize the temperature dependence of the refractive index (Fig. S11b). The data reveals a linear correlation (*n* = 3.063 − 0.051 × *T*) between the refractive index and temperature, confirming that the refractive index is quantitatively modulated by the thermal environment (Δ*T* → Δ*n*). By synthesizing this established Δ*n*–Δ*T* relationship with the Δ*E*_MLCT_–Δ*T* correlation determined in Step 1, the direct connection between the macroscopic refractive index and the molecular energy levels is established. To quantify this connection, the derivative d*n*/d*E*_MLCT_ is introduced to characterize the sensitivity of the local refractive index to molecular energy shifts (Fig. 4d). This analysis yields a high dispersion factor of 81.011 RIU/eV, providing the physical foundation for regulating plasmon resonances at the molecular scale via refractive-index control.

Finally, the refractive index change is transduced into a far-field angular shift (Step 3) via the grating-coupled momentum matching condition: (${k}_{0}\sin {\theta }_{{{{\rm{side}}}}}={k}_{{{{\rm{SPP}}}}}-\frac{2{{{\rm{\pi }}}}m}{\varLambda }$) where the *k*_SPP_ is a function of *n*. The sensitivity of this process depends on the derivative of the SPP dispersion relation with respect to the refractive index. By measuring the angular shift against the local index, the platform’s high transduction efficiency (d*θ*_side_/d*n*) was determined to be 13.507°/RIU (Fig. 4e).

By multiplying these three physically coupled components, the theoretical total sensitivity is calculated: *S*_calculated_ = (0.00062 eV/°C) × (81.011 RIU/eV) × (13.507°/RIU) ≈ 0.678°/°C. This calculated value exhibits strong agreement with the direct experimental measurement (0.580°/°C). This quantitative congruence validates the proposed mechanism, confirming that the sensor’s performance is driven by the precise cascading of molecular thermodynamics, Kramers–Kronig dispersion, and plasmonic momentum matching.

To further evaluate the universality and tunability of the proposed method, comparative assessments were performed on waveguides doped with Rhodamine B (RhB) and temperature-sensitive [Ru(phen)₃]Cl₂ in different polymer matrices (Fig. S12). The results indicate that the sensing mechanism is predominantly governed by the temperature-dependent refractive index of the polymer matrix, ensuring the applicability of the methodology to a wide range of fluorophores. However, specific fluorophores like [Ru(phen)₃]Cl₂, which exhibit temperature-dependent spectral shifts, synergistically enhance the refractive index modulation, thereby offering a tunable pathway to achieve higher sensitivity compared to fluorophores with stable emission wavelengths.

### *Sensor performance characterization and benchmarking*

**Fig. 5:**
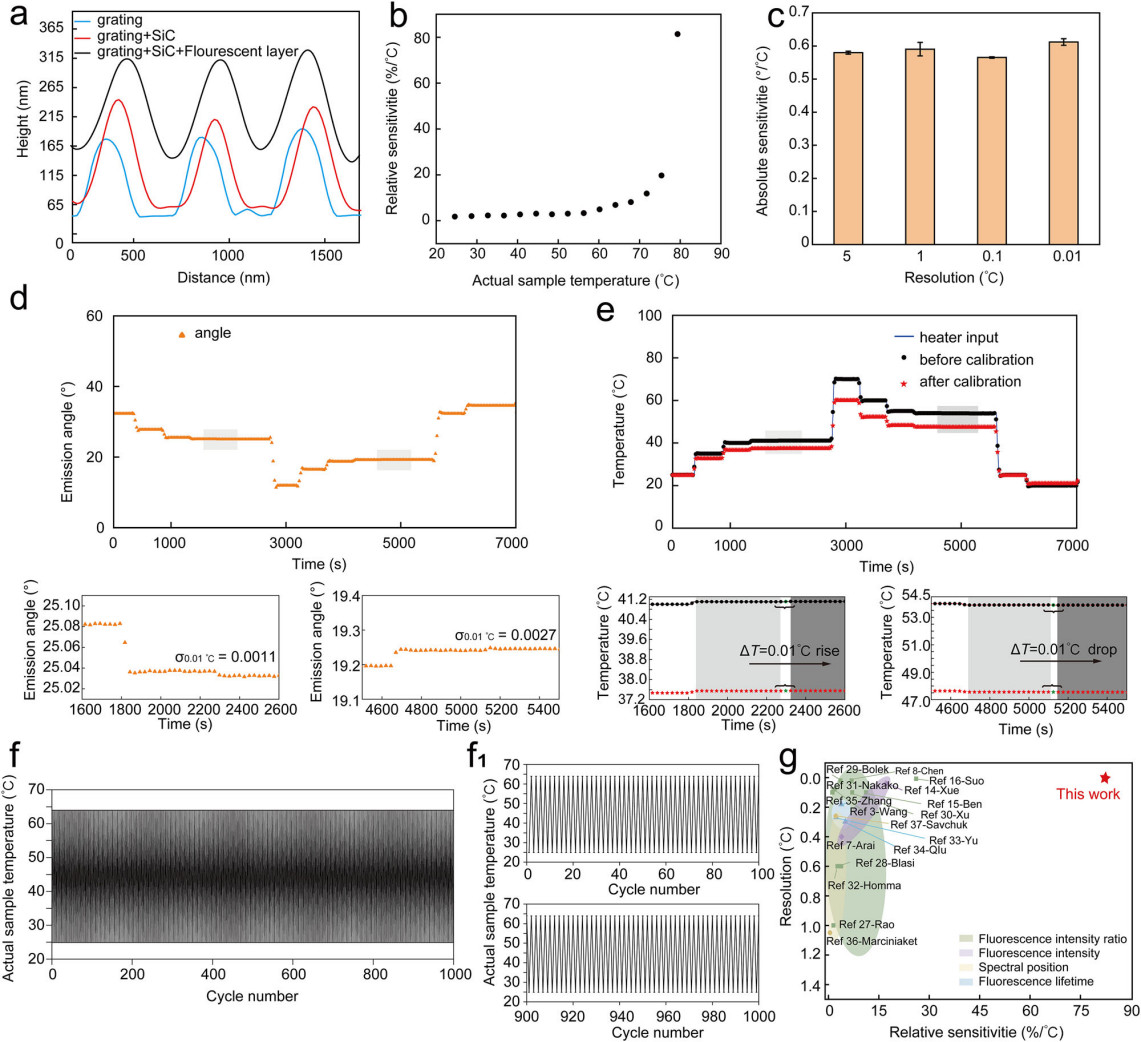
Comprehensive performance, high-resolution capability, and long-term stability. **a** Atomic force microscopy (AFM) cross-sectional profile verifying the fabricated sensor’s designed curved structure and layer thicknesses. **b** Relative sensitivity (*S*_rel_), defined as *S*_rel_ = |*S*_abs_/*θ*_side_|× 100%, plotted as a function of temperature. ${S}_{{{{\rm{rel}}}}}$ shows a pronounced exponential increase, reaching a maximum value of 81.34%/°C at 79.36 °C. **c** *S*_abs_ of the angle-based fluorescent temperature sensor at temperature resolutions of 5 °C, 1 °C, 0.1 °C, and 0.01 °C. Error bars represent the standard error (SE) of the linear fits. **d**, **e** Dynamic high-resolution step-change test demonstrating the practical achievement of 0.01 °C temperature resolution. **d** The temporal response of the raw emission angle and (**e**) the corresponding calibrated actual temperature profile clearly resolve 0.01 °C thermal steps with high signal fidelity. **f** Long-term reliability and repeatability demonstrated via an accelerated 1000-cycle thermal aging test between 24.91 °C and 63.95 °C (calibrated temperatures). Data points represent the mean of *n* = 5 independent consecutive measurements with error bars denoting the standard deviation (SD). Error bars are explicitly plotted but remain visually obscured because the calculated standard deviations are smaller than the data symbols. The full 1000-cycle trace shows stability. **f**_**1**_ An expanded view comparing the initial 100 cycles (top) and final 100 cycles (bottom), confirming no measurable drift or degradation in signal baseline or response amplitude. **g** Comparison of relative sensitivity and resolution performance for the angle-based sensor (this work), FIR-based^3,8,15,16,27–32^, fluorescence lifetime-based^33–35^, spectral position-based^36,37^, and fluorescence intensity-based^7,14^ temperature sensors.

Figure 5 presents a comprehensive characterization of the fabricated fluorescent temperature sensor, examining its morphology, stability, resolution, and detailed sensitivity metrics to validate its high-performance capabilities. First, the successful fabrication and structural integrity of the device were confirmed. In Figs. 5a and S13, schematic diagrams and atomic force microscopy (AFM) cross-sectional profiles verify the sensor’s designed structure, surface morphology, and layer thicknesses. The cross-sectional profile reveals a curved configuration with gold, SiC, and fluorescent layers measuring approximately 148, 23, and 103 nm, respectively.

With the physical structure and fabrication integrity validated, the sensor’s comprehensive thermal performance characteristics were quantitatively analyzed. Beyond the high *S*_abs_ established in Fig. 4, the sensor’s comprehensive performance is defined by its relative sensitivity (*S*_rel_). To facilitate comparison with other thermometry technologies, *S*_rel_ is defined as:

5 $${S}_{{{{\rm{rel}}}}}=\left|\frac{{S}_{{{{\rm{abs}}}}}}{{\theta }_{{{{\rm{side}}}}}}\right|\times 100\%$$

where *θ*_side_ is the reference emission angle at a given temperature. The behavior of *θ*_side_ across the operational temperature range is presented in Fig. 5b. It shows a pronounced exponential increase with rising temperature, reaching a maximum value of 81.34%/°C at 79.36 °C (Table S1).

A crucial validation is whether the sensor’s high absolute sensitivity is maintained when resolving minute temperature changes. Here, the standard sensitivity of 0.580°/°C, rigorously established via broad-range calibration (5 °C steps), serves as the benchmark. As summarized in Fig. 5c, sensitivities derived from fine-scale resolution tests at step increments of 1 °C, 0.1 °C, and 0.01 °C (0.591, 0.565, and 0.612°/°C, respectively) exhibit excellent agreement with this standard value (deviations <5%). This consistency confirms that the sensor maintains its high responsivity and linearity from macro- to micro-thermal regimes. Supported by high coefficients of determination (*R*^2^ > 0.98) even at the finest 0.01 °C scale (Fig. S14c₂), these results validate that the calibration equation established over the broad temperature range is universally applicable for high-resolution thermal monitoring. Building on this verified linearity, the sensor’s capability to achieve this 0.01 °C resolution dynamically is demonstrated in Figs. 5d, e and S13. The system was subjected to various thermal step-changes, with the inset highlighting the response to a step of 0.01 °C. The angular output (Fig. 5d) and the corresponding calibrated temperature (Fig. 5e) clearly resolve each 0.01 °C increment with high fidelity, exhibiting variance during both heating (0.0011) and cooling (0.0027), thereby demonstrating an effective temperature resolution of 0.01 °C.

Having established the sensor’s precision in the thermal domain, we further characterized its physical constraints in the spatial domain (Fig. S15). By employing a focused 532 nm laser to generate a localized heat source (effective diameter ~66 μm), the thermal diffusion profile on the substrate was imaged. The resulting full-width at half maximum of 731 μm characterizes the effective thermal spreading length. This result highlights a distinction inherent to contact thermometry where, although the grating structure supports micro-scale optical readout, the effective spatial resolution for thermal mapping is physically governed by the substrate’s heat diffusion and the establishment of thermal equilibrium.

To address critical concerns regarding long-term reliability and repeatability, the sensor was subjected to an accelerated 1000-cycle thermal aging test. The sensor was cycled continuously, with data acquisition performed under strict thermal equilibrium and averaged over five consecutive scans to ensure signal fidelity. The recorded emission angles were then converted into calibrated sample temperatures using the established relationship (*T*_actual_ = 80.653 − 1.724 × *θ*_side_). As a result, the actual sample temperature fluctuated stably between 24.91 °C and 63.95 °C throughout the test (detailed experimental conditions are described in Supplementary Note 2, and raw angular data are provided in Fig. S16). As shown in the full temperature trace over 1000 cycles (Fig. 5f), the sensor exhibited operational stability. A direct comparison between the initial and final 100 cycles (Fig. 5f₁) confirms that no measurable drift or degradation occurred in either the signal baseline or the response amplitude. This long-term cycling stability is further supported by static tests at fixed actual sample temperatures (24.60 °C, 40.64 °C, and 60.19 °C), which demonstrate minimal signal drift over a 2000-s period (Fig. S17).

Furthermore, the sensor’s robustness against environmental variables was characterized. The influence of humidity was quantified, showing the calibration curve is reliable across a wide operational range of 10%–89% relative humidity (RH), with the physical failure mechanism at saturation (≥95% RH) also identified (Fig. S18). In addition to humidity, the influence of oxygen quenching was evaluated across a pressure range from 3.8 Pa to 100 kPa. While the fluorescence intensity decreased by approximately 10% due to oxygen permeation, the emission angle remained stable with a standard deviation of 0.003°, demonstrating that the angular signal is effectively isolated from oxygen concentration variations (Fig. S19). Critically, the sensor’s immunity to excitation power fluctuations, a fundamental advantage of the angle-based paradigm over intensity-based methods, was rigorously verified through both static and dynamic tests (Fig. S20). Unlike fluorescence intensity, which exhibits a direct dependence on photon flux, the emission angle was confirmed to respond exclusively to the steady-state physical temperature rise induced by laser self-heating. Consequently, it maintained exceptional stability (standard deviation ≤ 0.007°) even under power modulations that induced fluorescence intensity fluctuations (intensity ratio > 3.4).

Finally, the performance of the angle-based fluorescent temperature sensor is benchmarked against representative state-of-the-art thermometry methods based on FIR, fluorescence lifetime, spectral position, and fluorescence intensity (Fig. 5g). This comparison highlights the improvements in both relative sensitivity and temperature resolution achieved by the angle-based paradigm presented in this work.

### *Application in high-fidelity scientific metrology and real-world monitoring*

The high sensitivity (*S*_abs_ = 0.58°/°C) and resolution (0.01 °C) established in Figs. 4 and 5 enable applications in both high-precision scientific metrology and robust real-world monitoring. First, the sensor’s measurement capabilities were validated. The 0.01 °C resolution was physically confirmed by monitoring the solid-liquid phase transition of Gallium. Using this specific calibration and a controlled slow heating rate (0.1 °C/min), the sensor resolved the flat isothermal plateau at the 29.76 °C melting point, a physical phenomenon that is typically obscured by conventional thermometers (Fig. S21). Furthermore, the sensitivity was demonstrated by directly quantifying the micro-scale laser self-heating effect. The sensor resolved the subtle temperature rise induced by laser power increments, confirming its ability to measure low-magnitude thermal phenomena (Fig. S22).

**Fig. 6:**
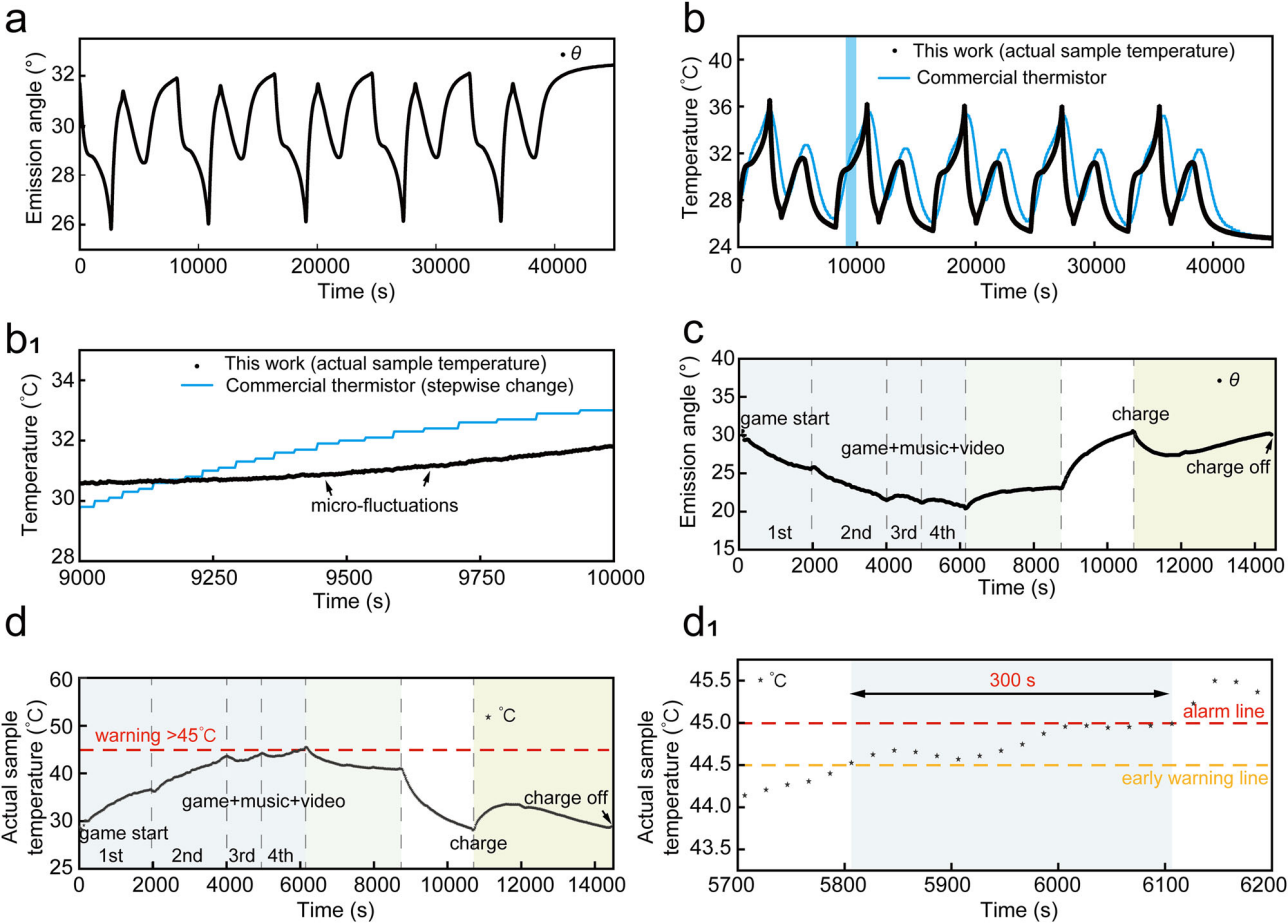
In situ thermal monitoring of battery and smartphone CPU operations. **a** Collected angle signal and **b** corresponding temperature during continuous charge–discharge cycles (6 A) of a battery. **b**_**1**_ Magnified view (from the blue region in b) highlighting subtle temperature fluctuations, where the temperature data from the commercial sensor exhibits a stepwise change over time. **c** Collected angle signal and **d** converted temperature, showing temperature evolution and a warning for a smartphone CPU in multitasking mode. **d**_**1**_ Magnified view from (**d**) demonstrating the sensor’s rapid response and early warning potential.

Building upon these established capabilities, Fig. 6 shows the application of an angle-based fluorescent temperature sensor for real-time surface temperature monitoring of a battery and a smartphone CPU under various operating conditions. Leveraging its 0.01 °C temperature resolution and high sensitivity, the sensor enables continuous monitoring of component thermal behavior. During each cycle, the fluorescence emission angle was continuously recorded and converted to temperature in real time using a pre-established calibration curve (Fig. 6a). Operating at 6 A, the five cycles spanned approximately 45000 s (~12 h). The sensor signal remained stable throughout this period, with measured surface temperatures ranging from 27.70 °C to 37.62 °C. Crucially, the 0.01 °C resolution captured subtle temperature fluctuations within each cycle. These fluctuations, potentially arising from internal electrochemical non-uniformities or early degradation signs, are challenging to resolve with conventional thermocouples, whose 0.1 °C resolution produces a stepwise temperature profile that obscures the true thermal dynamics (Fig. 6b_1_). Such monitoring provides valuable data for battery safety assessment and lifetime prediction. In the smartphone CPU monitoring experiments (Fig. 6c and d), the sensor tracked the rapid temperature rise from 27.93 °C to 44.51 °C during the transition between the first and second gaming sessions, resolving the dynamic details of this change. Notably, during the fourth gaming session, with simultaneous music and video playback, the sensor triggered the 45 °C threshold alert at 6106 s. Furthermore, the resolution enables the system to potentially predict overheating risks earlier than simple threshold triggering by analyzing changes in the temperature slope below 45 °C. For instance, the system detected a potential risk at 44.50 °C, 300 s before the alert threshold was reached (Fig. 6d_1_). This early warning capability provides quantitative data for advanced thermal management strategies, such as dynamic clock frequency adjustment, to extend hardware lifespan and enhance system reliability.

## Discussion

To overcome the inherent limitations of conventional fluorescence thermometry, specifically the susceptibility of intensity-based signals to non-thermal fluctuations, an angle-based sensing paradigm has been developed. This strategy fundamentally shifts the sensing dimension from signal magnitude to angular position. By harnessing the precise modulation of molecular dipole transitions, the sensor achieves a high relative sensitivity of 81.34%/°C and an absolute sensitivity of 0.580°/°C. The origin of this performance was elucidated through a quantitative deconstruction of the cascaded mechanism, validating the precise coupling of molecular thermodynamics, Kramers–Kronig dispersion, and plasmonic momentum matching. Critically, the sensor’s superiority extends beyond sensitivity to robustness. It demonstrated long-term stability during demanding battery cycling and, unlike conventional methods, maintained high fidelity even under drastic excitation power modulations. The high resolution (0.01 °C) enabled a 300 s advance warning against CPU overheating, highlighting its potential for proactive thermal management. Regarding spatial capability, while the optical readout supports micro-scale interrogation, the thermal resolution is physically governed by the establishment of thermal equilibrium and contact interface dynamics rather than the intrinsic optical limit. Moreover, the sensor’s customizability and compatibility with large-area fabrication (Fig. S23) position it as a scalable solution. These results establish the grating-modulated fluorescence emission angle strategy as an approach for probing microscale heat flow, laying a solid foundation for next-generation thermal-sensing technologies.

## Methods

### *Sample preparation and post-treatment*

Initially, a 50 nm thick Ge–Sb–Te (GST) film was deposited onto 1 mm thick JGS1 quartz substrates using magnetron sputtering (KYKY, 500CK-500ZF). A 50 nm thick Au film was then thermally evaporated (VNANO, VZZ-300S) onto the GST film, forming a GST/Au bilayer structure. This bilayer was immersed in a 7 wt% aqueous (NH₄)₂S solution for 30 min to selectively etch the GST layer, yielding a patterned Au film on the quartz substrate. Following this, a dielectric layer (SiO₂, SiN*ₓ*, or SiC; ~20 nm thickness) was sputtered (Kurt J. Lesker PVD) onto the patterned Au film to mitigate potential fluorescence quenching. Subsequently, to finalize the device fabrication and investigate the matrix-dependent sensing characteristics, three distinct polymer-dye composite layers were prepared. For the primary flexible sensor, [Ru(phen)₃]Cl₂ (Sigma-Aldrich) was dissolved in an aqueous PVA (Macklin, *M*_w_ ≈ 190000, >98% hydrolyzed) solution to achieve a 1 wt% concentration. To establish a spectral reference, RhB (Aladdin) was dissolved in a separate aqueous PVA solution under continuous stirring to ensure homogeneity. For the rigid matrix counterpart, a PMMA (Macklin) composite was prepared via a co-solvent strategy, wherein [Ru(phen)₃]Cl₂ was initially dissolved in ethanol under ultrasonication and subsequently blended with a PMMA/acetone solution. Finally, the respective precursor solutions were spin-coated onto the Au/dielectric substrates and dried at 60 °C for 24 h to remove residual solvents and solidify the functional fluorescent layers.

### *Laser machining*

Laser machining was performed using an ultrafast laser system (Light Conversion; 515 nm wavelength, 300 fs pulse width, maximum pulse energy of 140 μJ). The laser beam was first shaped into a 5-mm line profile using a cylindrical lens and then focused onto the sample surface with a 10× objective lens (NA = 0.25). The working distance and scanning trajectory during the machining process were controlled by an XYZ motorized stage (Newport, models XMS100-S and VP-5ZA).

### *Optical simulation*

The optical response of the patterned substrates was modeled using FDTD simulations, conducted with the Lumerical software package. Detailed simulation parameters and settings are provided in the Supplementary Note 2 (see section Optical simulation details).

### *Morphology detection*

Surface morphological analysis of the samples was conducted using atomic force microscopy (AFM; Bruker, Dimension Edge) and scanning electron microscopy (SEM; ZEISS, Merlin Gemini 2). AFM imaging was performed in tapping mode to minimize potential sample damage.

**Fluorescence emission angle collection:** A 473 nm laser (Industries Optoelectronics Technology Co., Ltd., model MBL-III-473) served as the excitation source for the fluorophores, with the excitation light incident perpendicularly onto the sample surface. During fluorescence collection, the sample was maintained at an approximate distance of 10 cm from the collecting lens surface. Emitted signals were collected by spherical lenses and subsequently directed to a spectrometer (Zolix, model Omni-λ 300i) for analysis. Angle-resolved measurements, covering a range from −180° to 180°, were enabled by two rotating stages (Zolix, models TBR200 and TBR60L, with an angular resolution of 0.001°), operating at a rotation speed of 1°/s. The data acquisition rate of the spectrometer was set to be higher than the rotational speed of the stages to ensure sufficient spectral collection at each angle. Precise temperature control of the sample was maintained using a water-cooled thermal stage (INSTEC, model TS62, USA) with a resolution of 0.001 °C. Temperature-dependent fluorescence emission angle data were collected continuously and automatically via computer software.

### *Data acquisition protocols*

To ensure measurement reliability and minimize random noise, standard experimental protocols were implemented. For all steady-state measurements (including calibration, humidity characterization, and thermal aging tests), the sample was maintained at the setpoint for at least 5 min (or until the reading stabilized) to achieve thermodynamic equilibrium. Unless otherwise specified, each reported angular data point is derived from five internal instrumental accumulations to optimize the signal to noise ratio. Where applicable, linear regression analyses were performed to extract sensitivity parameters, with fitting errors expressed as the standard error (SE). For the Gallium phase transition experiment, a slow, controlled heating ramp rate of 0.1 °C/min was employed to resolve the latent heat plateau.

### *Temperature-dependent refractive index characterization*

The temperature-dependent refractive indices of the constituent materials were characterized using a spectroscopic ellipsometer (Horiba, TF-UVISEL) equipped with a thermal stage. To ensure statistical reliability, these specific data were obtained from three independent sample measurements with the final results expressed as mean values accompanied by their standard deviations.

### *Surface temperature monitoring and early warning in critical components*

To demonstrate the early warning capability for overheating, a lithium-ion battery (4422 mAh) was subjected to cycling tests using a battery cycler (YPSDZ, 3010). For comparative analysis, the surface temperature was simultaneously monitored using a commercial dual-channel K-type thermocouple thermometer (UNI-T, UT320D) with a resolution of 0.1 °C. The thermocouple probe was attached to the sample surface adjacent to the fluorescence sensor using heat-resistant polyimide tape to ensure accurate co-location comparison. For the early warning test on a critical component, a smartphone (Xiaomi 11, equipped with a Qualcomm Snapdragon 888 processor) was utilized. To induce a thermal load, the smartphone was remotely operated via a screen-casting function to execute a combination of computationally intensive tasks.

## Supplementary information

Supplementary Information

Transparent Peer Review file

## Source data

Source Data

## Data Availability

The data generated in this study are provided in the Supplementary Information and Source Data file. Specifically, the source data underlying Figs. 1–6 and Supplementary Figs. 1–22 are provided as a Source Data file with this paper. Source data are provided with this paper.

## Code availability

The optical simulations were performed using Lumerical FDTD Solutions (Ansys). The thermal simulations were performed using COMSOL Multiphysics. The data acquisition and analysis were performed using standard software packages (e.g., Origin). No custom algorithms or software were developed for this study.
