# Supplementary material for: Angle-based fluorescence thermometry with high sensitivity and high resolution
Source: Nat Commun. 2026 May 22;17:6723. doi: 10.1038/s41467-026-73430-4 (PMC13385821; doi:10.1038/s41467-026-73430-4)
Supplement: Supplementary file 1 — Supplementary Information [file 41467_2026_73430_MOESM1_ESM.pdf]

Supplementary Information

Angle-based fluorescence thermometry with high sensitivity and resolution

Xuanzheng Zhou [1], Kang Xu [2], Zekai Li [1], Yuxuan Dong [1], Jinghui Chao [1], Yufei Zhai [1], Ying Jin [1], Shaolin Xu [2], Min Wang [1, 3, *]

[1] School of Microelectronics, Southern University of Science and Technology, Shenzhen 518055, PR China

[2] Department of Mechanical and Energy Engineering, Southern University of Science and Technology, Shenzhen 518055, PR China

[3] State Key Laboratory of Quantum Functional Materials, Southern University of Science and Technology, Shenzhen 518055, PR China

*Corresponding authors' e-mail:

wangm@sustech.edu.cn.

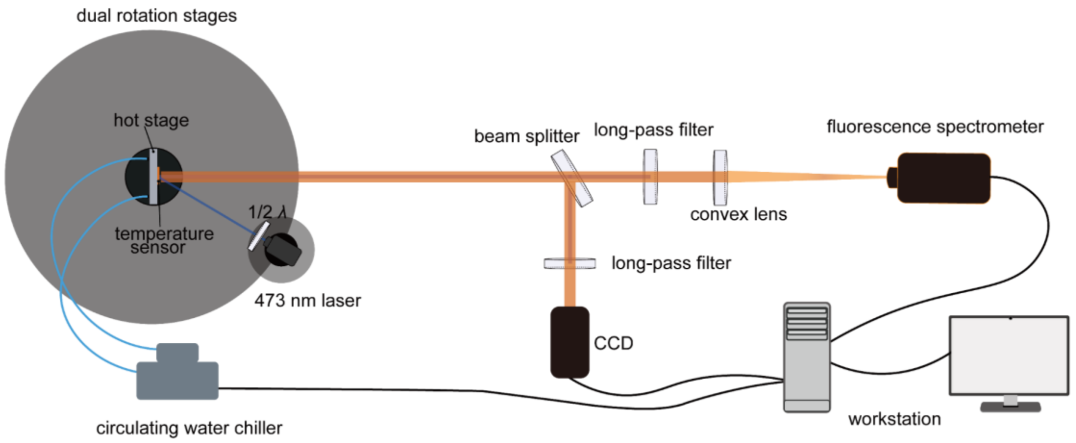

Figure S1. Schematic of the optical measurement setup. Schematic of the angle-resolved fluorescence measurement setup featuring a high-precision water-cooled temperature stage, laser excitation source, dual rotation stages, and spectrometer.

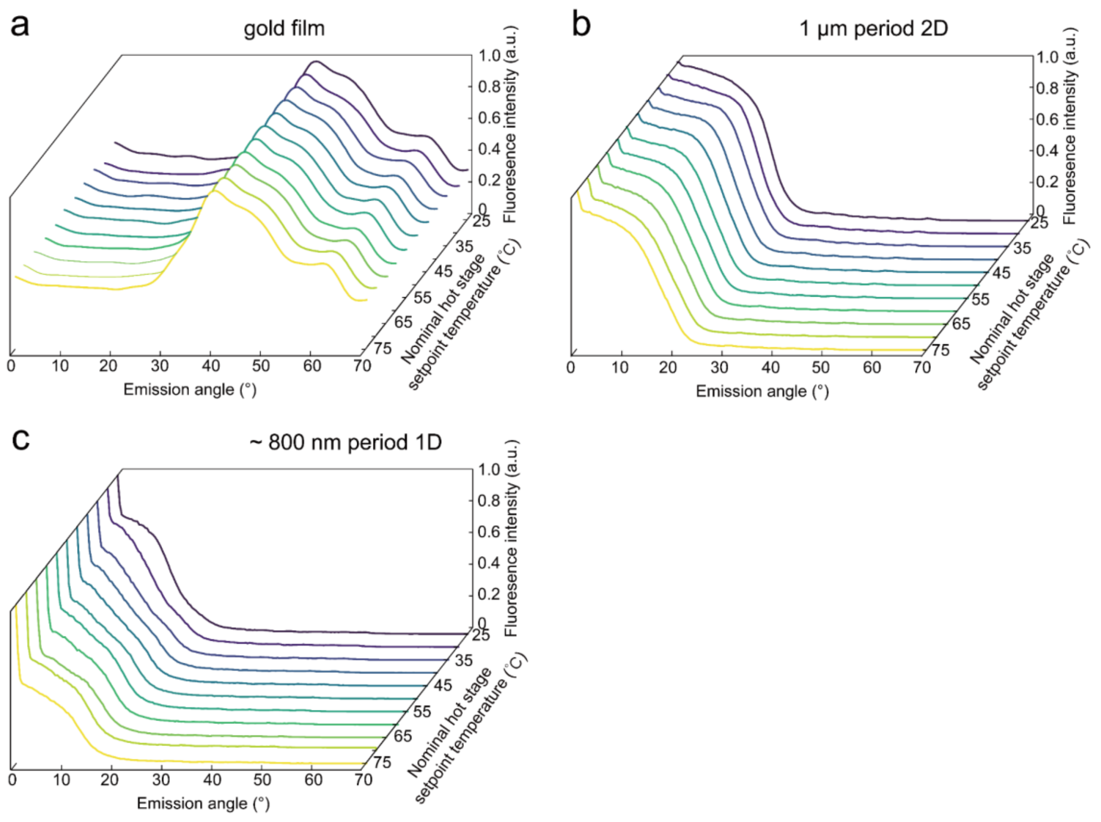

Figure S2. Temperature response of control nanostructures. a) Conventional gold film displays a single primary diffraction peak at approximately 45° that is independent of temperature. b) Two-dimensional (2D) disordered structures produce no distinguishable fluorescence emission angles or temperature response. c) One-dimensional (1D) gold gratings with mismatched periods (～800 nm) exhibit no discernible fluorescence emission angles or temperature-dependent response.

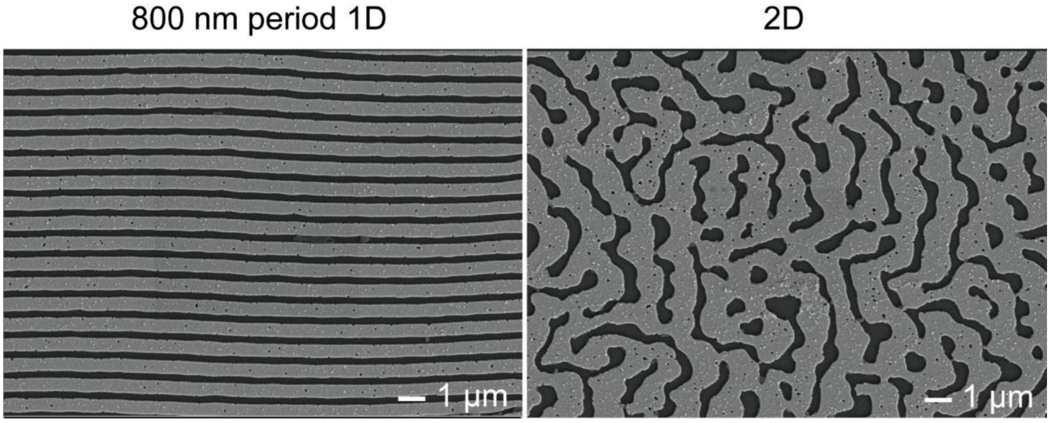

Figure S3. SEM characterization of grating structures. SEM images of the ~800 nm period 1D gold grating and the 1 μm period 2D gold grating.

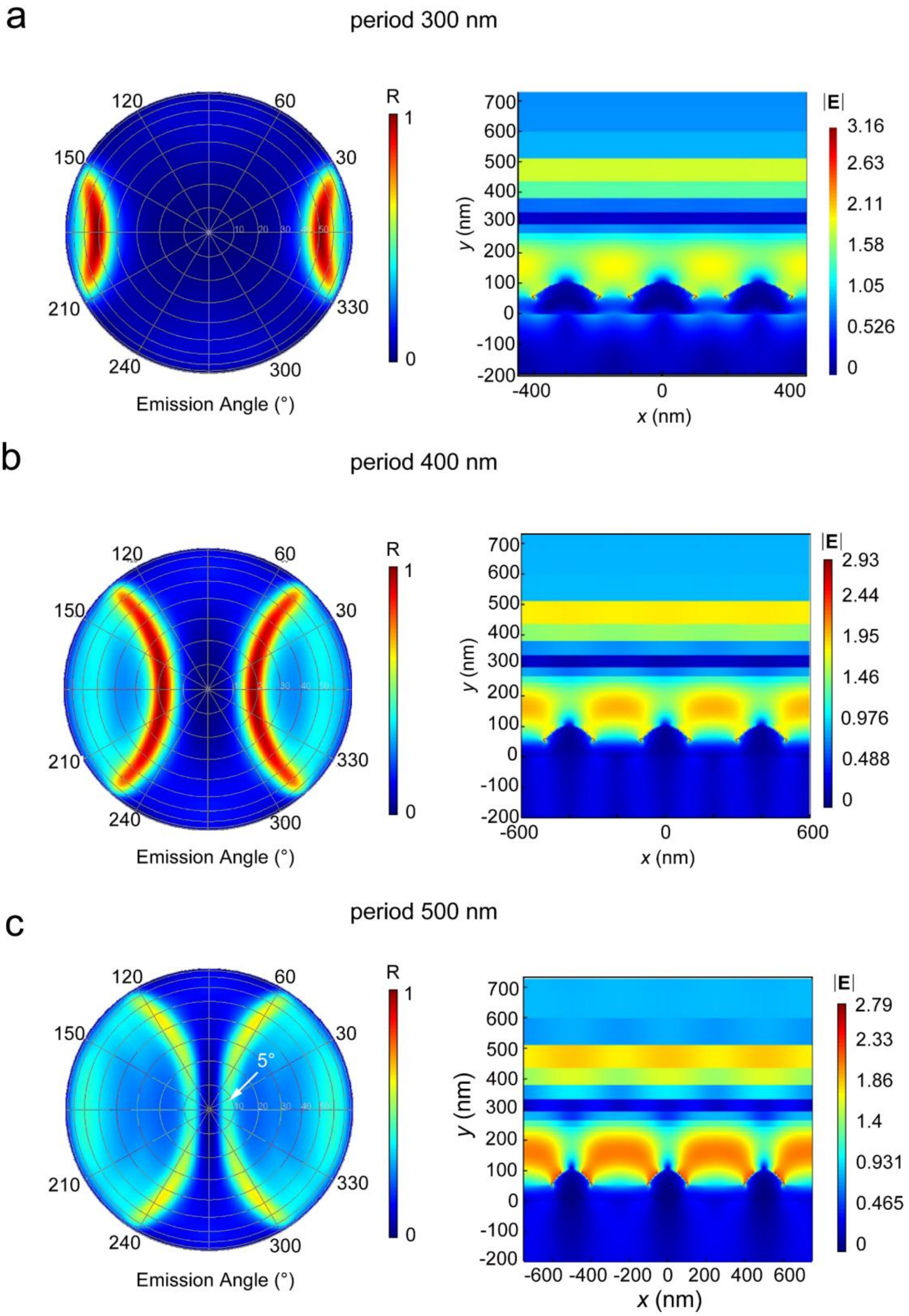
a
period 300 nm
120
60
150
30
210
330
240
300
Emission Angle (°)
R
1
0
|E|
3.16
2.63
2.11
1.58
1.05
0.526
0
700
600
500
400
300
200
100
0
-100
-200
y (nm)
-400
0
400
x (nm)
b
period 400 nm
120
60
150
30
210
330
240
300
Emission Angle (°)
R
1
0
|E|
2.93
2.44
1.95
1.46
0.976
0.488
0
y (nm)
-600
0
600
x (nm)
c
period 500 nm
120
60
150
30
5°
210
330
240
300
Emission Angle (°)
R
1
0
|E|
2.79
2.33
1.86
1.4
0.931
0.465
0
y (nm)
-600 -400 -200 0 200 400 600
x (nm)

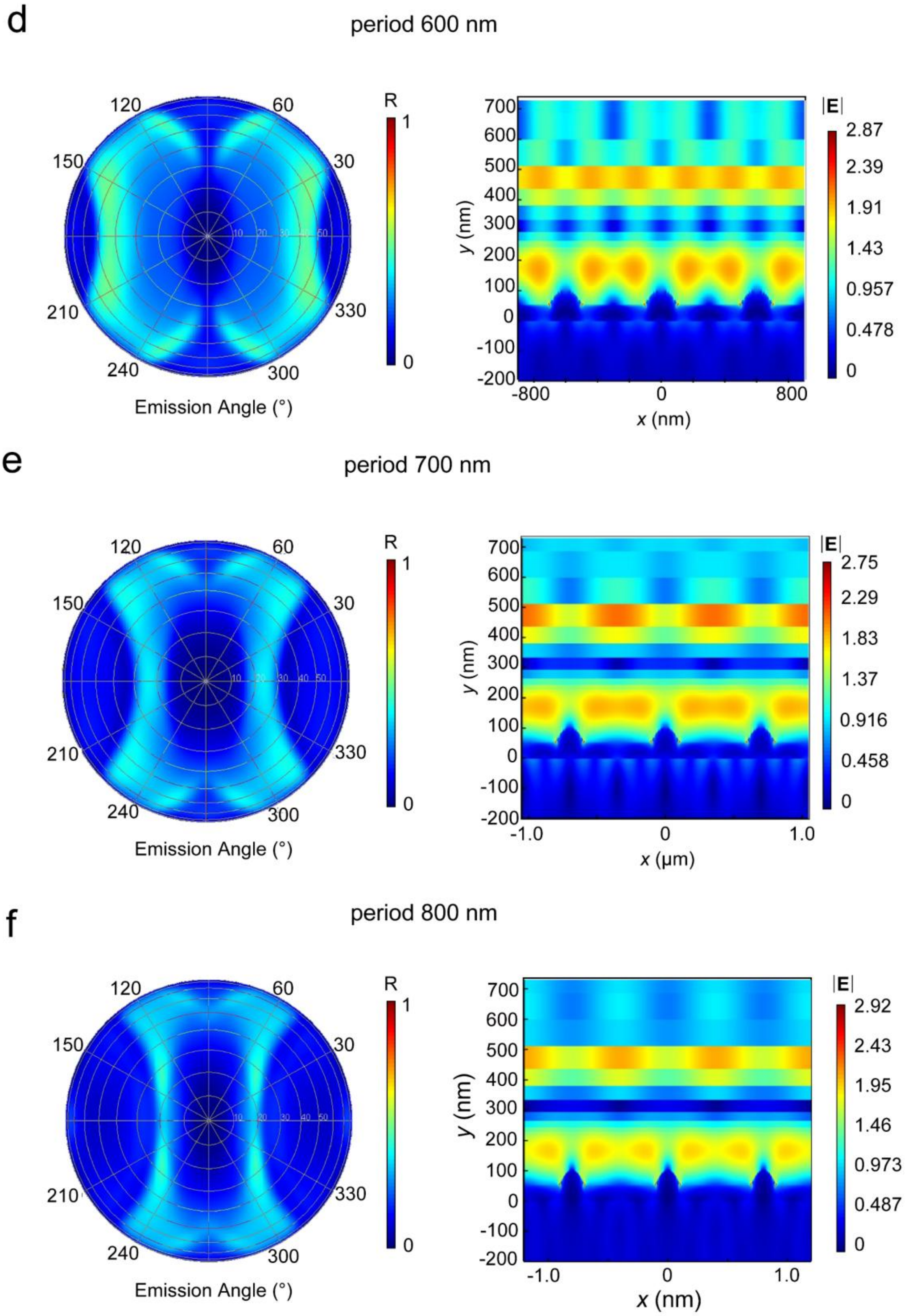
d
period 600 nm
120
60
150
30
210
330
240
300
Emission Angle (°)
R
1
0
|E|
2.87
2.39
1.91
1.43
0.957
0.478
0
y (nm)
x (nm)
-800
0
800
e
period 700 nm
Emission Angle (°)
|E|
2.75
2.29
1.83
1.37
0.916
0.458
0
x (μm)
-1.0
1.0
f
period 800 nm
Emission Angle (°)
|E|
2.92
2.43
1.95
1.46
0.973
0.487
0
x (nm)
-1.0
1.0

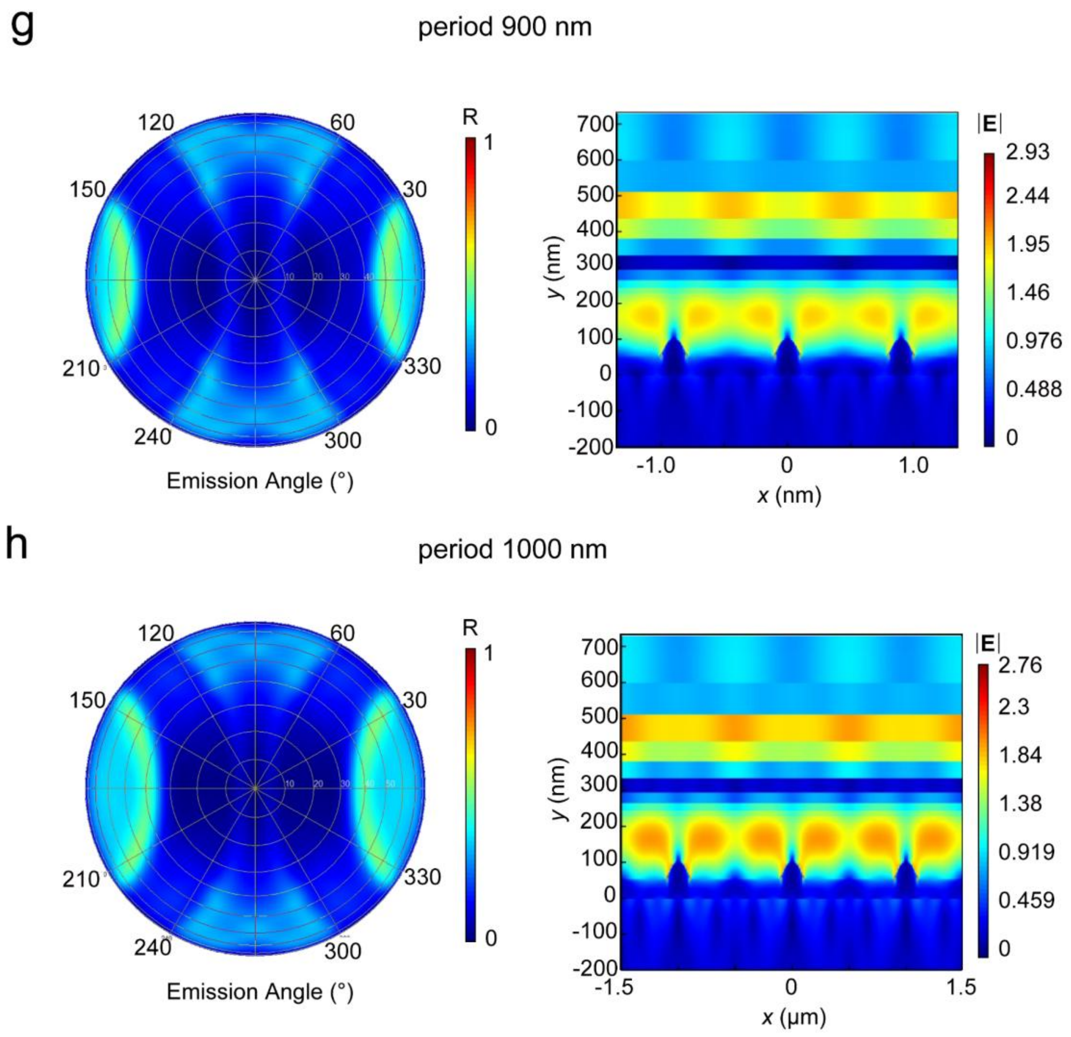

Figure S4. FDTD simulations of gratings with varying periods. a-h) Simulation of far field diffraction angles (left column) and near-field electric field magnitude distribution (right column) for 300-1000 nm periods.

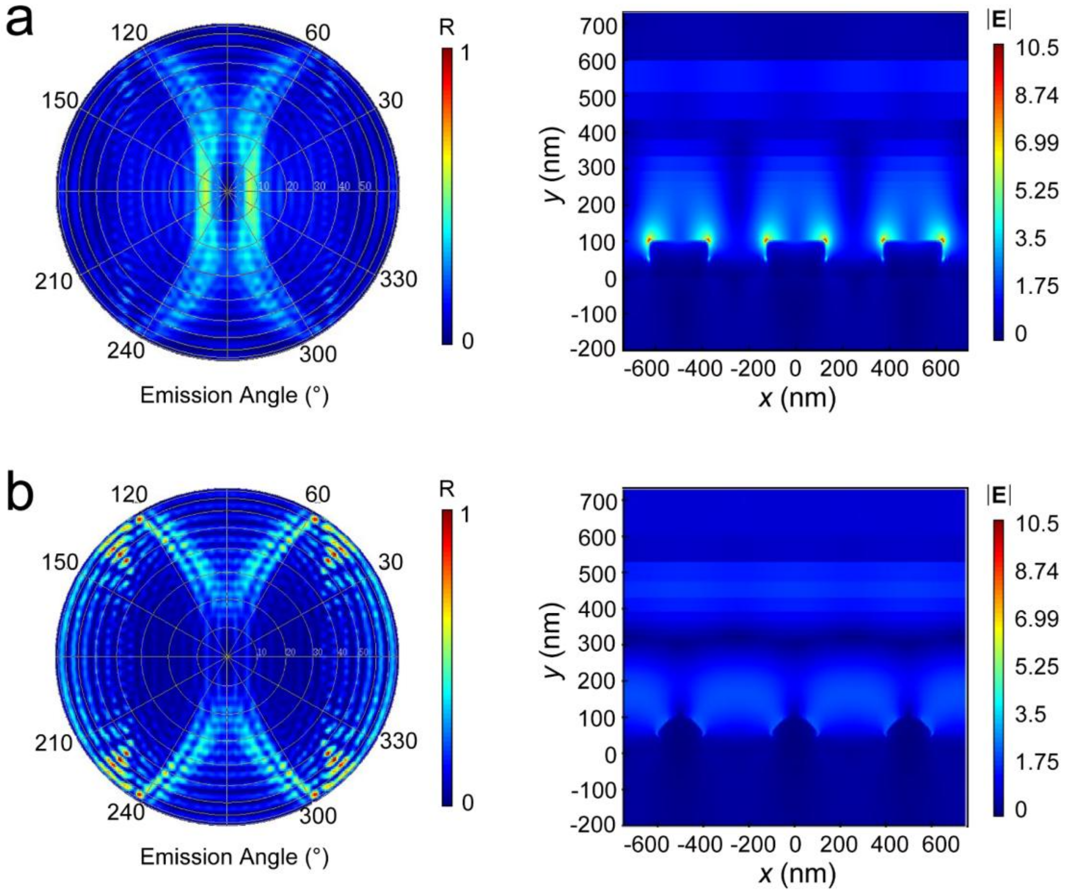

Figure S5. FDTD simulations of continuous grating morphologies. Simulation of the far-field diffraction angle map (left column) and near-field electric field magnitude distribution (right column) of a) continuous rectangular and b) continuous curved gratings.

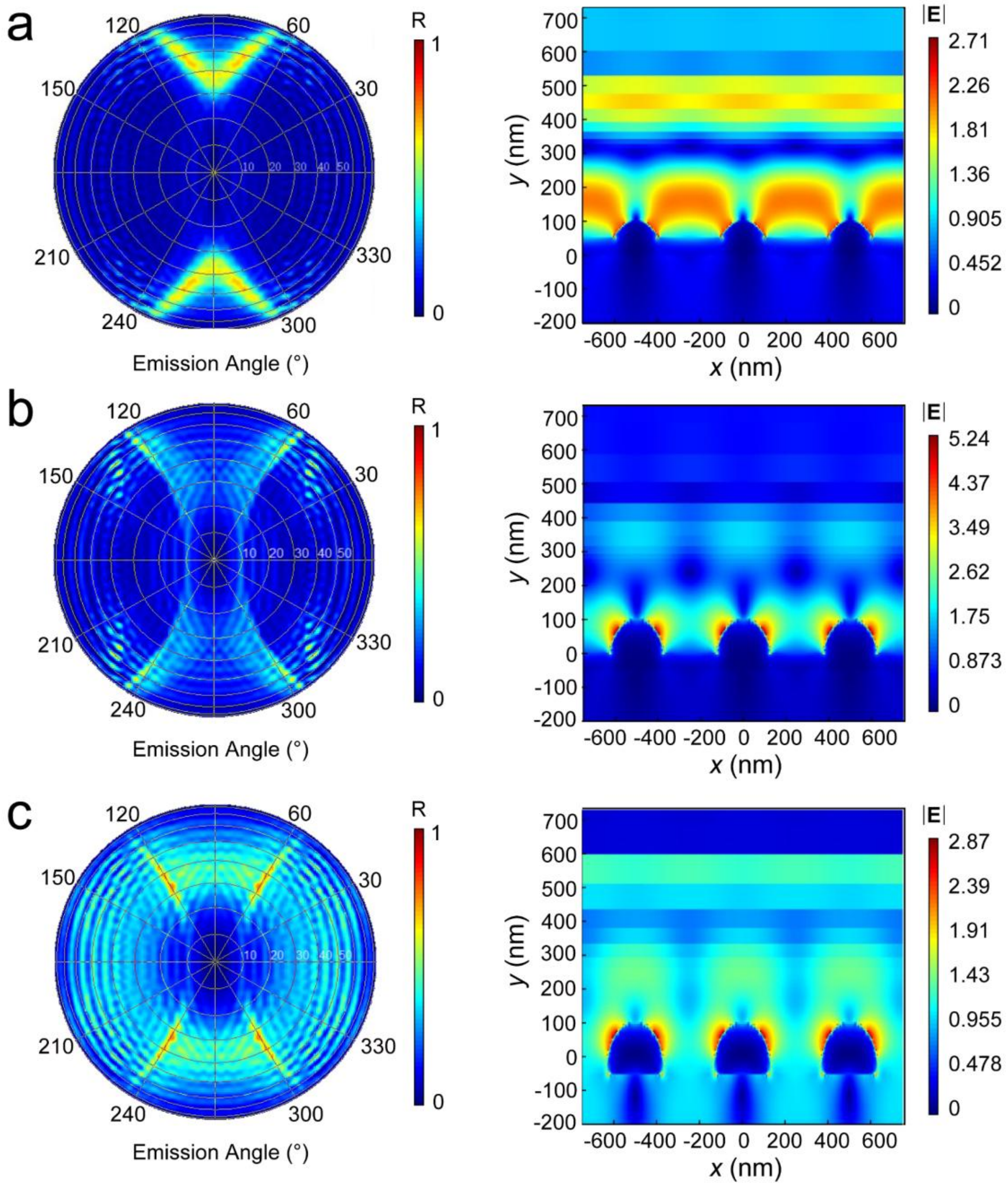

Figure S6. Effect of grating thickness on optical response. Simulation of the far-field diffraction angle map (left column) and near-field electric field magnitude distribution (right column) of a) 100, b) 150 and c) 200 nm thick gratings.

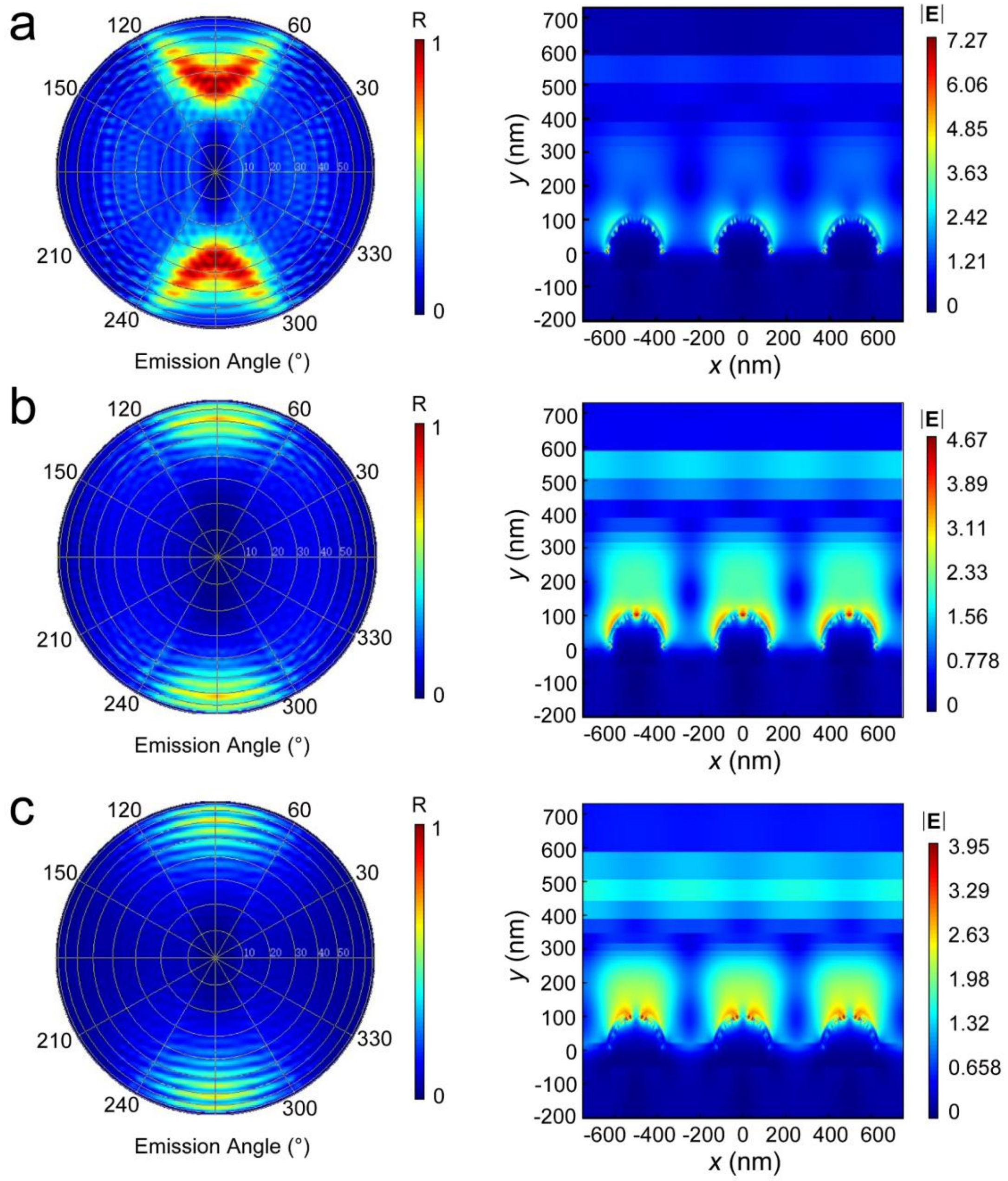

Figure S7. Effect of dielectric coating materials. Simulation of the far-field diffraction angle map (left column) and near-field electric field magnitude distribution (right column) of 150 nm grating coated with 20 nm a) SiO_2, b) SiN*x* and c) SiC.

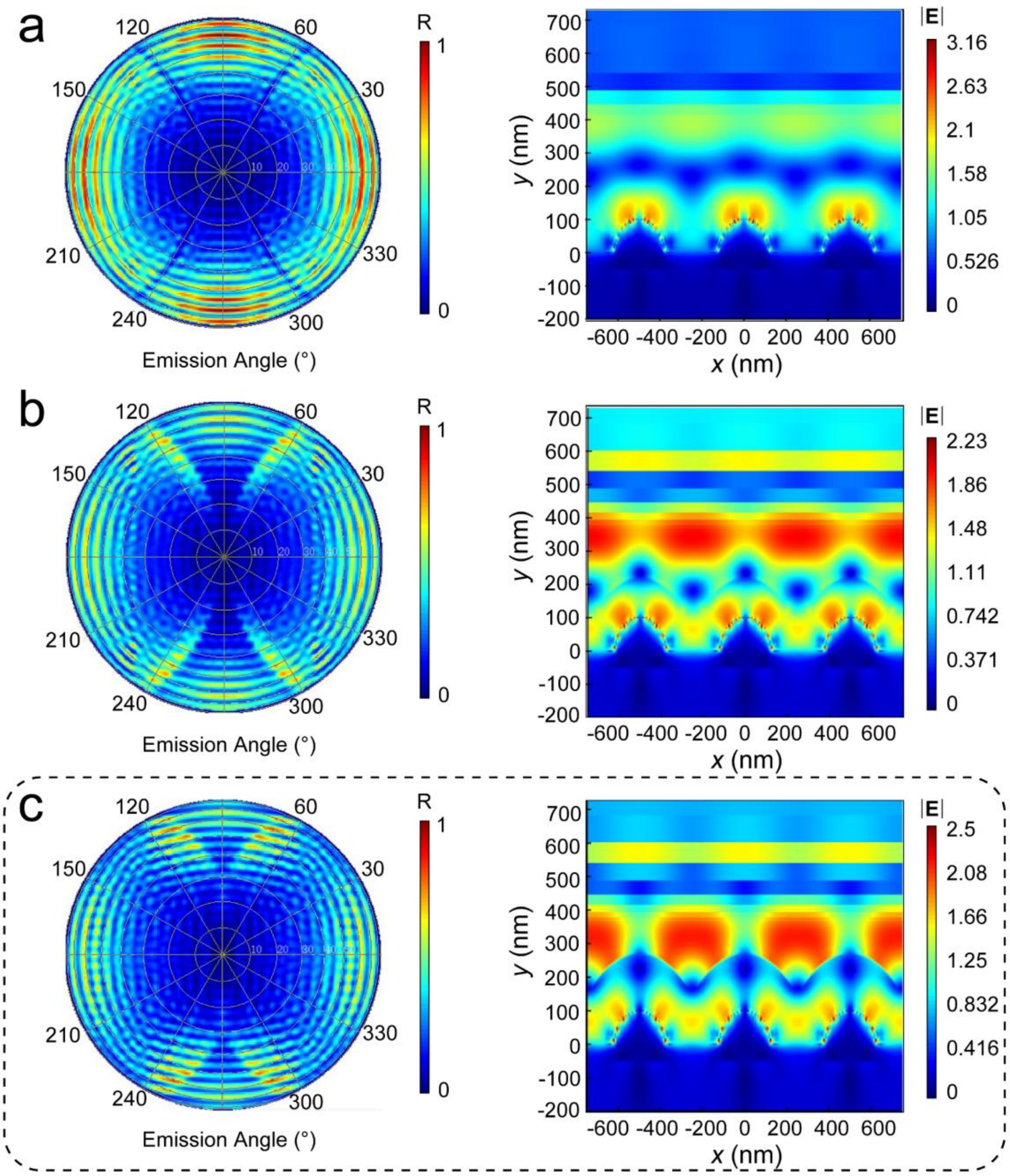

Figure S8. Effect of fluorescent layer thickness. Simulation of the effect of fluorescent-layer thicknesses of a) 50 nm, b) 100 nm and c) 150 nm on the far-field diffraction angle map (left column) and near-field electric field magnitude distribution (right column) for a 150 nm gold grating with 20 nm SiC. Dashed boxes in c) highlight that these distributions correspond to the optimized parameters presented in Fig. 3d of the main text.

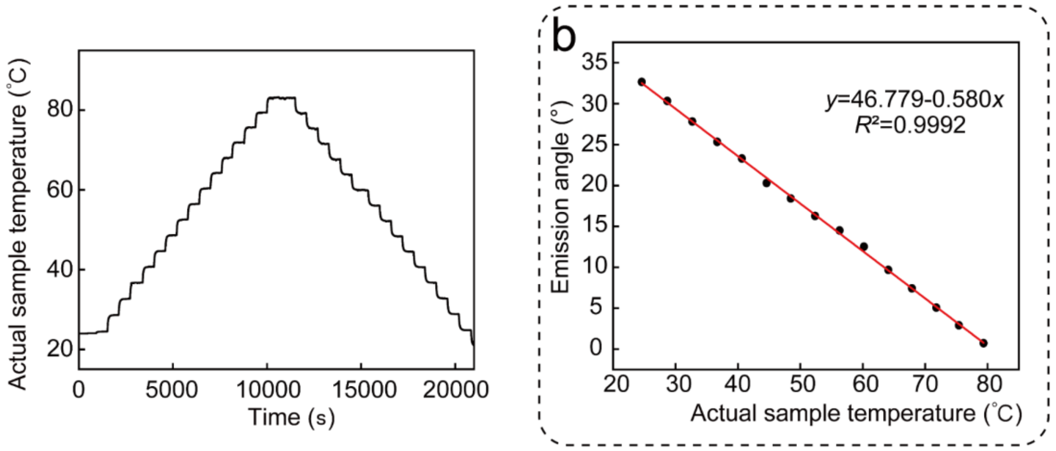

Figure S9. In-situ temperature calibration and characterization of thermal resistance. a) Temporal profile of the actual sample surface temperature (T_{actual}) measured by a Pt RTD attached directly to the top surface of the sensor chip (adjacent to the sensing area) during a stepped heating and cooling cycle (setpoints: 25 °C to 100 °C). The data reveals the thermal lag and the magnitude of the temperature drop across the substrate. b) The final calibration curve relating the sensor's emission angle (θ_{side}) to the measured actual temperature (T_{actual}). To compensate for the thermal hysteresis observed in panel a, each data point plotted here represents the arithmetic mean of the temperatures measured during the heating and cooling phases. The data shown in this panel (highlighted by the dashed box) is identical to the calibration curve presented in the main text Fig. 4b. The response is linear (R^2= 0.9992, sensitivity = 0.580 °/°C), confirming the sensor's accuracy after correcting for the thermal resistance of the experimental setup.

The in-situ calibration data reveals a significant and temperature-dependent discrepancy between the hot stage setpoint (T_{set}) and the actual sample surface

temperature (T_{actual}). As illustrated in Figure S9a, T_{actual} is lower than T_{set}, with the deviation increasing at higher temperatures (e.g., reaching approximately 17 °C at a setpoint of 100 °C). This substantial thermal drop confirms the significant thermal resistance introduced by the 1-mm-thick quartz substrate, underscoring the necessity of direct surface temperature measurement to eliminate potential errors. Furthermore, a minor thermal hysteresis (< 0.5°C) was observed between the heating and cooling phases, attributable to the thermal mass of the system. To mitigate this artifact, the calibration standard was derived from the average T_{actual} of the heating and cooling cycles. Figure S9b presents the final calibration curve, plotting the fluorescence emission angle (θ_{side}) against the calibrated T_{actual}. The sensor exhibits a linear response ($R^2 > 0.999$) described by the equation $\theta_{\text{side}} = -0.580 \times T_{\text{actual}} + 46.779$. This high linearity, achieved after correcting for substrate thermal resistance, validates the intrinsic reliability of the sensing mechanism. Consequently, all temperature data reported in this work have been corrected using this in-situ calibration.

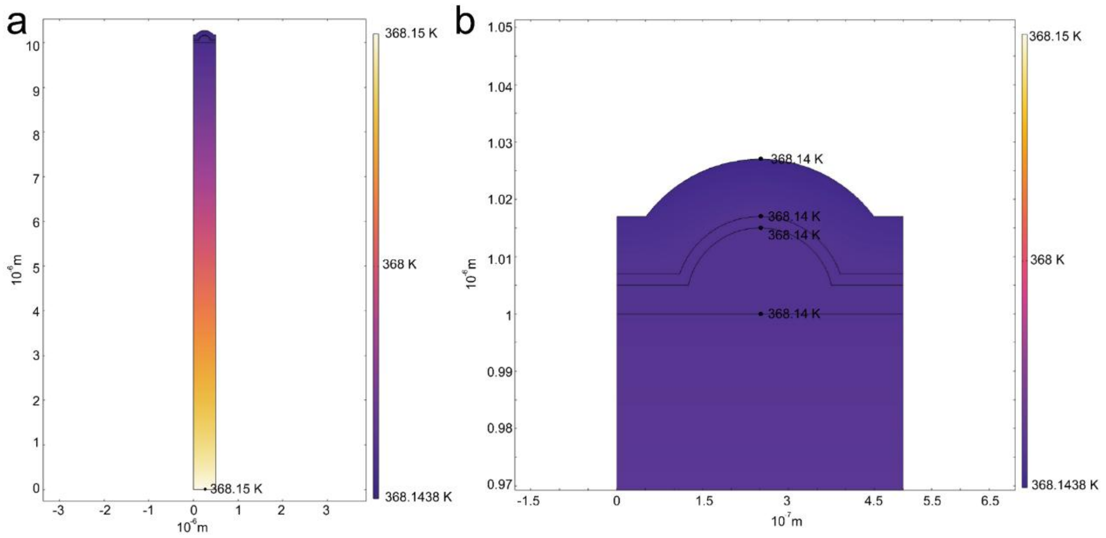

Figure S10. Finite element simulation of the internal thermal distribution within the sensor structure. a) The steady-state temperature field of the full sensor stack modeled using COMSOL Multiphysics under bottom heating at 95 °C. The simulation domain includes the 1-mm-thick quartz substrate and the top functional layers. b) Magnified view of the top functional region from a, illustrating the temperature distribution across the gold grating (150 nm), SiC dielectric (20 nm), and PVA fluorescent layer (100 nm). Quantitative analysis of the vertical temperature profile along the central axis reveals a negligible temperature difference ($\Delta T_{\text{micro}} < 1$ mK) between the top surface of the fluorescence layer and the metal-dielectric interface (368.14 K), confirming that the sensing volume remains thermally uniform and validating the effective temperature assumption used in the sensing principle.

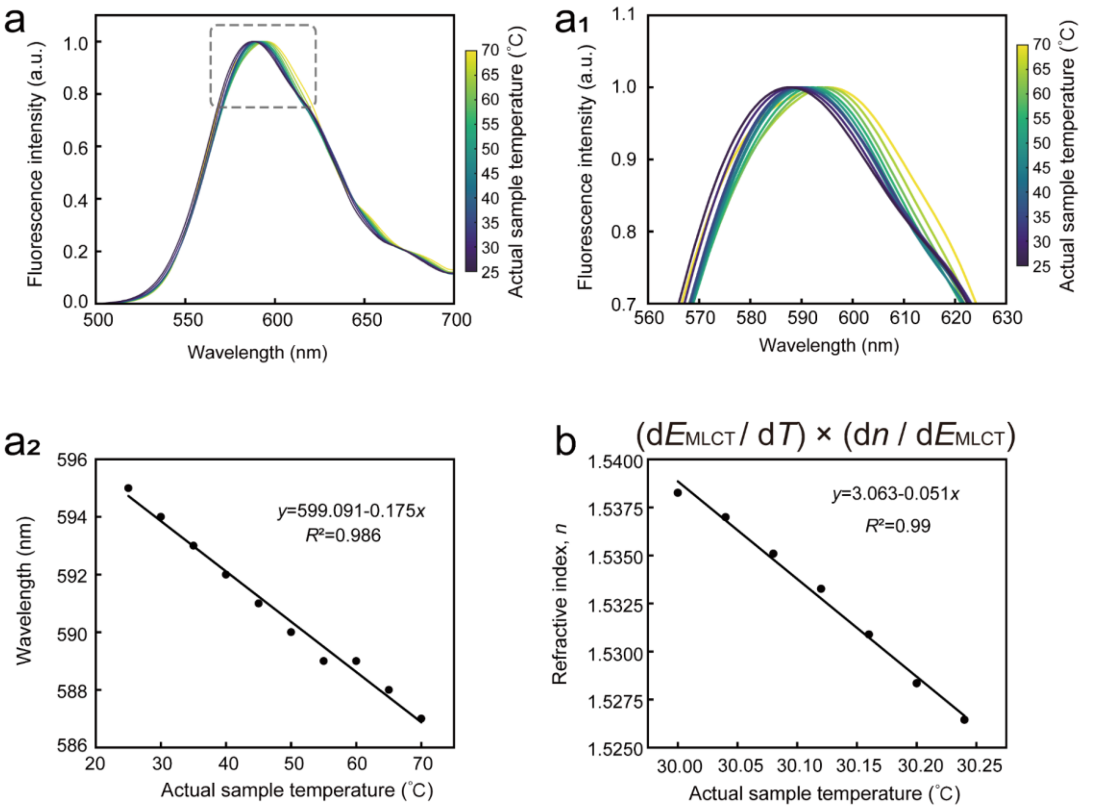

Figure S11. Characterization of molecular thermochromic shift and refractive index dispersion. a) Temperature-controlled fluorescence spectra of $[Ru(phen)_3]Cl_2$ showing a linear blue shift of the MLCT emission peak with increasing sample temperature. a_1) Magnified view of the spectral region in a highlighting the peak shift. a_2) Linear fitting of the emission peak wavelength versus temperature, yielding a slope of 0.175 nm/°C ($R^2 = 0.986$). b) Temperature-dependent refractive index of $[Ru(phen)_3]Cl_2$ measured via spectroscopic ellipsometry, revealing a linear decrease described by the relation $y = 3.063 - 0.051x$. Data points represent the mean ± s.d. of *n*=3 independent technical replicates, and error bars are smaller than the symbol size.

Supplementary Note 1

In-situ temperature calibration

To precisely characterize the sensor's thermal response, the actual sample surface temperature (T_{actual}) was measured directly using a calibrated platinum resistance thermometer (Pt RTD, 2-mm-diameter probe, Taizhou Nuowei Mechanical and Electrical Equipment Co., Ltd., China; accuracy 0.03 °C). The Pt RTD was mounted adjacent to the sensor on the heating stage using high-thermal-conductivity silicone grease to eliminate interfacial air gaps and minimize contact thermal resistance, thereby ensuring strict thermal equilibrium. A high-precision source meter (Keithley DMM 6500) recorded the RTD resistance via a four-wire configuration. The system was subjected to a controlled thermal cycle: heating from 25 °C to 95 °C and subsequently cooling to 25 °C. At each step, both the hot stage setpoint (T_{set}) and T_{actual} were continuously monitored and recorded synchronously with the sensor's angular output (θ_{side}). To account for minor thermal hysteresis inherent to the setup, the final reported T_{actual} for each calibration point was derived by calculating the arithmetic mean of the temperatures measured during the heating and cooling phases.

Theoretical derivation and experimental characterization of the cascaded transduction mechanism

To validate the sensitivity deconstruction presented in Fig. 4, a theoretical derivation of the constituent physical processes governing the sensor's response is provided below, supported by experimental characterization. The total sensitivity is

mathematically expressed via the chain rule as the product of three partial derivatives: $S_{\mathrm{abs}} = \mathrm{d}\theta_{\mathrm{side}}/\mathrm{d}T = (\mathrm{d}E_{\mathrm{MLCT}}/\mathrm{d}T) \times (\mathrm{d}n/\mathrm{d}E_{\mathrm{MLCT}}) \times (\mathrm{d}\theta_{\mathrm{side}}/\mathrm{d}n)$.

The initial transduction phase, molecular thermochromism ($\mathrm{d}E/\mathrm{d}T$), originates from the temperature-dependent modulation of the local dielectric environment. Predicated on Onsager reaction field theory[1,2], the stabilization energy of a polar excited state is a function of the host matrix's dielectric constant (ε) and refractive index (n). As temperature (T) increases, thermal expansion reduces the matrix density (ρ), leading to a decrease in the local dielectric constant ($\partial\varepsilon/\partial T < 0$). This reduction weakens the reaction field stabilization of the excited state, increasing the emission energy (E_{em}). This dependence is approximated by the temperature derivative of the Lippert-Mataga polarity function:

$$\frac{dE_{em}}{dT} = \frac{\partial E}{\partial \varepsilon} \cdot \frac{\partial \varepsilon}{\partial T} \tag{S1}$$

Since $\partial\varepsilon/\partial T$ is negative and stabilization energy scales with ε, the net result is a positive energy shift (blueshift). Experimentally, this temperature sensitivity is substantiated by temperature-controlled fluorescence spectroscopy (Fig. S9a). Temperature-controlled fluorescence spectroscopy reveals that the MLCT emission peak exhibits a linear blue shift of 0.175 nm/°C ($R^2 = 0.986$). This shift is primarily attributed to the alteration of the local dielectric environment upon heating, which expands the energy gap between the excited and ground states. By converting the emission wavelength to photon energy ($E = hc/\lambda$), this process yields the measured energy shift coefficient: $\mathrm{d}E/\mathrm{d}T = 0.00062$ eV/°C.

Subsequently, the causal link between the molecular energy shift (ΔE_{MLCT}) and the macroscopic refractive index change (Δn), which is quantified by the term ($\mathrm{d}n/\mathrm{d}E$) is governed by the Kramers-Kronig (K-K) relations[3,4]. The real part of the refractive index, $n(\omega)$, is determined by the integral of the imaginary part (extinction coefficient, κ) over all frequencies:

$$\Delta n(\omega) = \frac{1}{\pi}\mathcal{P}\int_{-\infty}^{\infty} \frac{\Delta\kappa\left(\omega^{'}\right)}{\omega^{'} - \omega} d\omega^{'} \tag{S2}$$

where $\mathcal{P}$ denotes the Cauchy principal value and ω represents the integration frequency variable. A thermally induced shift in the absorption/emission band (Step 1) alters the spectral distribution of $\kappa\left(\omega^{'}\right)$, which inherently necessitates a change in $n(\omega)$ in the transparency region. This can be analytically approximated using the Lorentz oscillator model near a resonance energy E_0: $n^2(E) = 1 + \frac{AE_0^2}{E_0^2 - E^2}$, where A denotes the dimensionless oscillator strength parameter associated with the transition. Differentiating n with respect to the resonance center E_0 yields the dispersion sensitivity:

$$\frac{dn}{dE_0} \approx \frac{A \cdot E \cdot E_0}{(E_0^2 - E^2)^2} \tag{S3}$$

This theoretical derivation provides the physical basis for the experimental correlation established in the main text, where the dispersion factor was empirically derived by synthesizing temperature-dependent ellipsometry data ($\mathrm{d}n/\mathrm{d}T$) with the spectroscopic shift ($\mathrm{d}E/\mathrm{d}T$). The model reveals that the high magnitude of this derived factor (81.011 RIU/eV) arises because the denominator term $(E_0^2 - E^2)^2$ approaches zero as the photon energy E nears the resonance E_0, causing the derivative $\mathrm{d}n/\mathrm{d}E_0$ to

diverge. Consequently, the high sensitivity observed in Fig. 4d is fundamentally attributed to the sensor operating within the regime of resonant dispersion enhancement near the material's absorption edge.

The final transduction step is governed by the momentum matching condition for surface plasmon polariton (SPP) coupling ($d\theta_{\mathrm{side}}/dn$): $k_0 \sin\theta_{\mathrm{side}} = k_{\mathrm{SPP}} - m\frac{2\pi}{\Lambda}$, where k_0 is the free-space wavevector, Λ is the grating period, and m is the diffraction order. The SPP wavevector is given by $k_{\mathrm{SPP}} = k_0\sqrt{\frac{\varepsilon_m n^2}{\varepsilon_m + n^2}}$, where ε_m represents the permittivity of the metal. The sensitivity is the derivative of the emission angle with respect to the refractive index: $\frac{\mathrm{d}\theta_{\mathrm{side}}}{\mathrm{d}n} = \frac{1}{k_0 \cos\theta_{\mathrm{side}}} \frac{\partial k_{\mathrm{SPP}}}{\partial n}$. The FDTD-optimized grating structure maximizes the field confinement, thereby maximizing the effective index sensitivity $\partial k_{\mathrm{SPP}}/\partial n$. This results in the measured platform gain of 13.507 °/RIU.

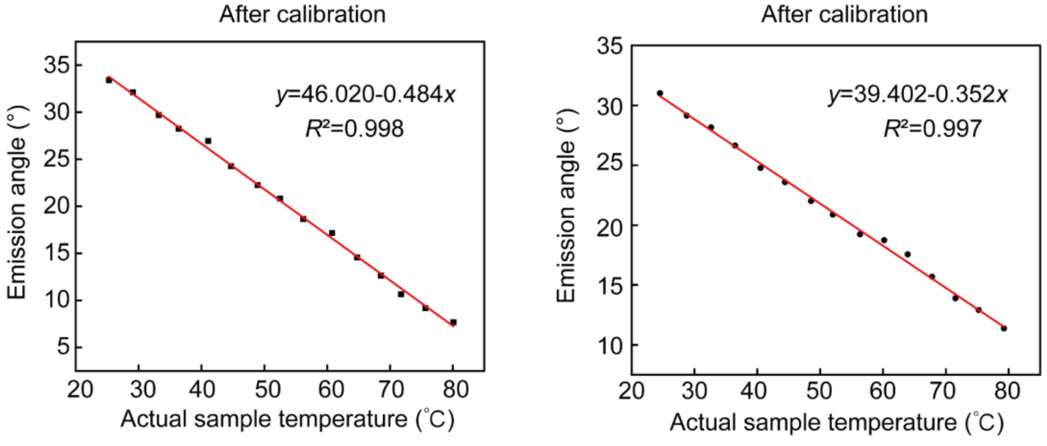

Figure S12. Comparative thermal sensitivity analysis using alternative fluorophore and matrix configurations. a) Temperature dependence of the fluorescence emission angle for the PVA matrix doped with Rhodamine B. The measured sensitivity is 0.48 °/°C. b) Temperature dependence of the emission angle for the PMMA matrix doped with $[Ru(phen)_3]Cl_2$. The measured sensitivity is 0.35 °/°C.

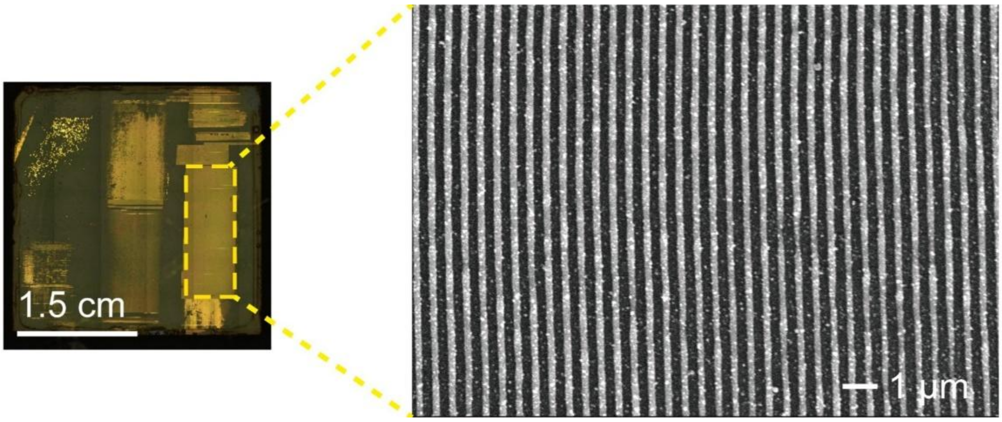

Figure S13. Morphology and microscopic characterization of the fluorescent temperature sensor. Photograph of the angle-based fluorescent temperature sensor sample and its SEM images.

Table S1. Fluorescence emission angle and corresponding relative sensitivity (S_{rel}) from 25 °C to 95 °C.

Temperature before calibration (°C)	Temperature after calibration (°C)	Angle (°)	S_{rel} (%)
25	24.60	32.65	1.78
30	28.70	30.34	1.97
35	32.71	27.81	2.27
40	36.67	25.31	2.24
45	40.64	23.31	2.72
50	44.59	20.29	3.09
55	48.47	18.40	2.81
60	52.37	16.26	3.07
65	56.28	14.50	3.30
70	60.19	12.52	4.93
75	64.08	9.69	6.85
80	67.87	7.42	8.12
85	71.76	5.06	11.87
90	75.40	2.90	19.72
95	79.36	0.71	81.34

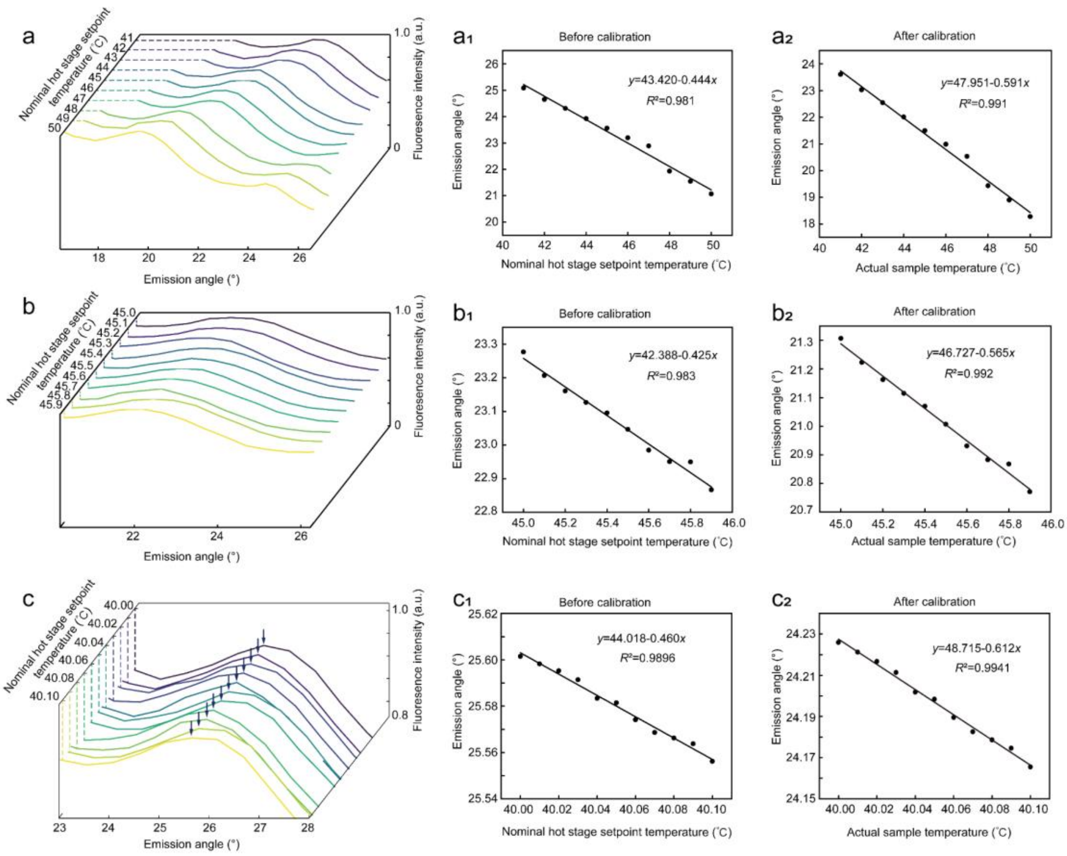

Figure S14. Verification of sensor linearity and resolution limits across varying thermal increments. a–c) Normalized angle-resolved fluorescence spectra recorded with temperature step sizes of a)1 °C, b) 0.1 °C, and c) 0.01 °C. Spectra are normalized to unity to highlight the θ_{side} independent of intensity variations. a_1-c_1) Linear fitting of the extracted peak emission angle as a function of the T_{set}. a_2-c_2) Corresponding calibration curves plotted against the calibrated actual sample temperature (T_{actual}). The high coefficient of determination ($R^2 > 0.98$) across all datasets confirms a robust linear response and a thermal resolution limit of 0.01 °C.

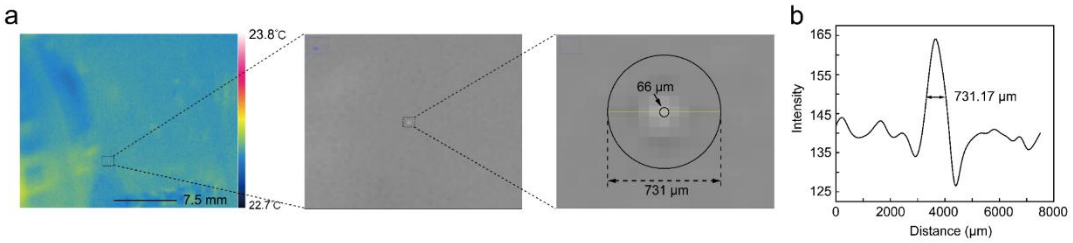

Figure S15. Quantification of the spatial resolution limit via thermal point spread function (PSF). a) Infrared thermal imaging of the sensor surface under localized heating. A separate focused 532 nm continuous-wave laser (20 × objective, NA = 0.8) was employed as a point-like heat source. The beam was controlled to generate a measured effective excitation spot diameter of 66 μm on the gold grating surface. b) Cross-sectional thermal intensity profile extracted from the laser-induced hot spot in Fig. S15a (yellow cross-section). The spatial resolution of the sensor platform is defined as the full width at half maximum (FWHM) of this thermal distribution. The measured FWHM of 731 μm confirms that the effective spatial resolution is governed by the lateral thermal diffusion length within the 1-mm-thick quartz substrate, rather than the intrinsic optical limit of the sensing method.

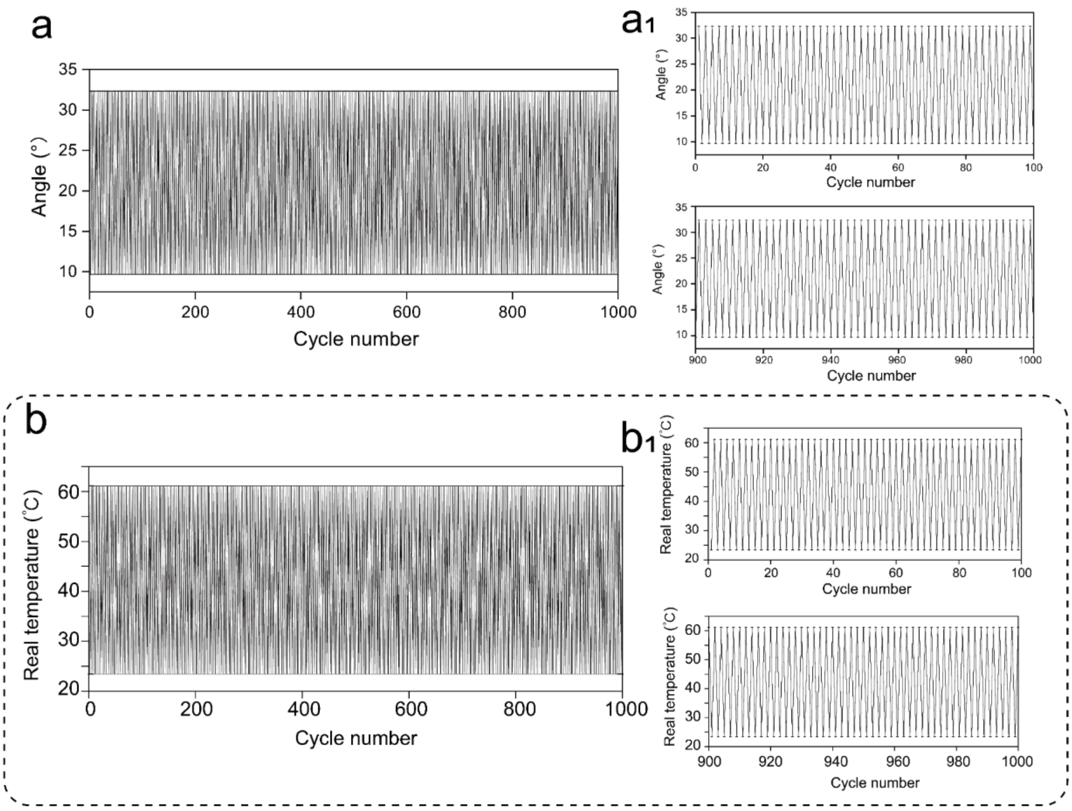

Figure S16. Long-term reliability and repeatability demonstrated via an accelerated 1000-cycle thermal aging test. a) Periodic variations of the fluorescence emission angle over 1000 accelerated thermal aging cycles. To ensure measurement precision and eliminate random noise, the sample was allowed to reach thermal equilibrium at each step, and each plotted point represents the arithmetic mean of five consecutive angular scans. a_1) An expanded view comparing the initial 100 cycles (top) and final 100 cycles (bottom), confirming no measurable drift or degradation in signal baseline or response amplitude. b) The corresponding calibrated actual sample temperature profile derived from the angular data (T_{actual} range: 24.91 °C to 63.95 °C). b_1) Expanded views of the temperature trace for the first 100 cycles (top) and the last 100 cycles (bottom), confirming the sensor's stability and lack of drift over the extended

testing period. Note that the temperature data presented in Figure 5f and 5f$_1$ in the main text are reproduced from the dashed box sections of this figure (panels b and b$_1$, respectively). For all panels, data points represent the mean of n = 5 independent consecutive measurements with error bars denoting the standard deviation (SD). Error bars are explicitly plotted but remain visually obscured because the calculated standard deviations are smaller than the data symbols.

To evaluate long-term reliability, the sensor was subjected to 1000 consecutive thermal cycles in a standard laboratory environment (60% RH). The temperature of the stage was modulated using a high-precision controller between setpoints of 25 °C and 75 °C, with holding periods at extremes to ensure equilibrium. During each cycle, the angular signal was derived from the average of five repetitive acquisitions to ensure data robustness.

The raw θ_{side} was continuously monitored over the entire duration. As shown in Fig. S16a, the emission angle oscillated between approximately 32.33° and 9.69°. No measurable drift in the signal baseline or degradation in the response amplitude was observed even after 1000 cycles (Fig. S16a$_1$). Subsequently, these raw angular data were converted into T_{actual} using the established calibration equation ($T_{\text{actual}} = 80.653 - 1.724 \times \theta_{\text{side}}$). The resulting temperature profile (Fig. S16b) confirms that the sensor reliably tracked the thermal cycling, with the actual sample temperature oscillating between 24.91 °C and 63.95 °C. A direct comparison between the initial 100 cycles and the final 100 cycles (Fig. S16b$_1$) reveals almost identical thermal profiles. These results, which are summarized as Fig. 5f and 5f$_1$ in the main text, verify that the

sensor maintains its calibration integrity and performance even after extensive thermal stress, effectively addressing concerns regarding durability for long-term applications.

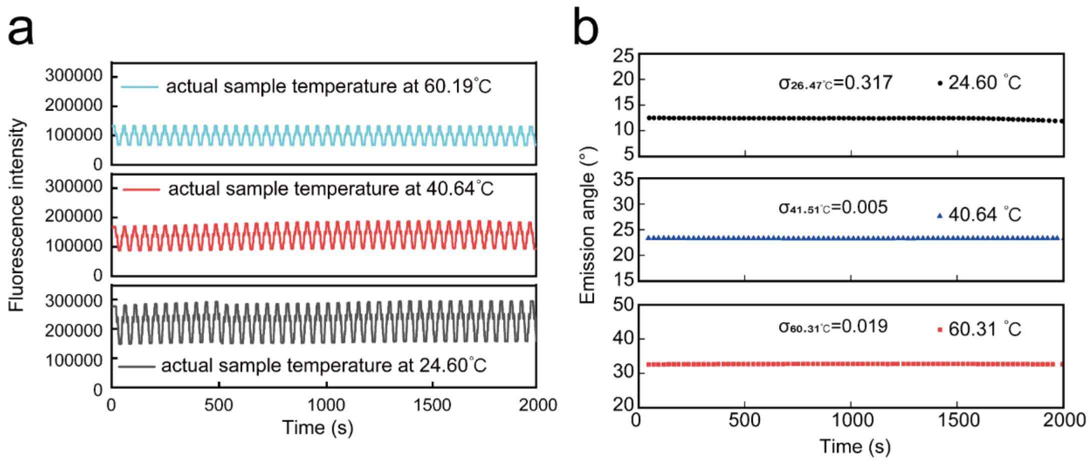

Figure S17. Dynamic stability of the fluorescent temperature sensor. a) Real-time raw fluorescence signals of the angle-based fluorescent temperature sensor measured at 24.60 °C, 40.64°C and 60.19 °C over a duration of 0–2000 s. b) Fluorescence emission angle of the angle-based fluorescent temperature sensor measured at 24.60 °C, 40.64 °C and 60.19 °C over a duration of 0–2000 s.

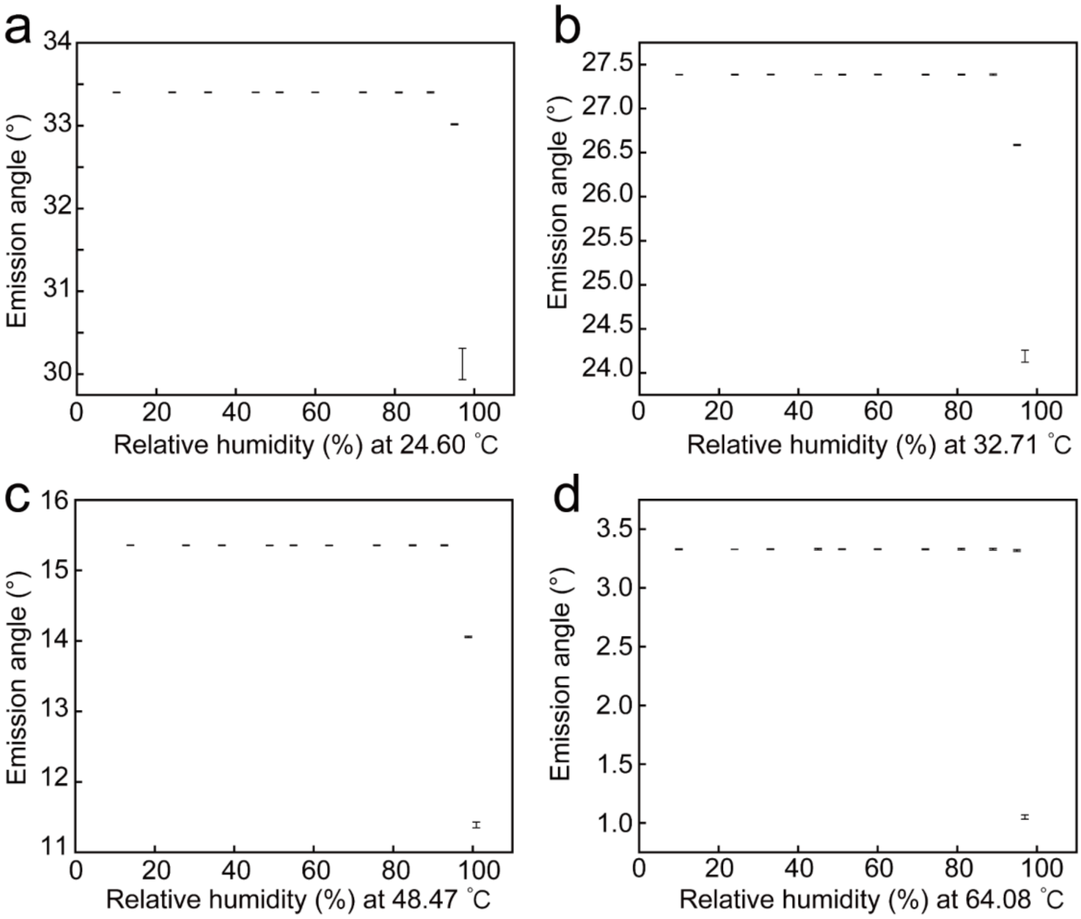

Figure S18. Quantification of environmental humidity influence on sensor performance. a-d) Fluorescence emission angle recorded as a function of relative humidity (RH) ranging from 10% to 97% at four constant, calibrated temperatures: a) 24.60 °C, b) 32.71 °C, c) 48.47 °C, and d) 64.08 °C. To ensure the hygroscopic equilibrium of the PVA sensing layer, the sensor was maintained at each humidity step for 5 minutes prior to data acquisition. The sensor demonstrates stability with negligible angular drift across the broad operational range of 10% – 89% RH. A distinct failure mode is observed only at near-saturation levels (≥ 95% RH), where a sharp decrease in emission angle occurs. This drop is attributed to the hygroscopic swelling of the PVA matrix, which simultaneously reduces the effective refractive index and increases the

grating period, physically altering the resonance condition. All data points represent the mean ± SD of 3 independent measurements.

The experimental results (Fig. S18) demonstrate stability across a broad humidity range. Between 10% and 89% RH, the fluorescence emission angle remained virtually constant at all tested temperatures. For instance, at 48.47 °C, the angle fluctuated negligibly between 15.358° and 15.356° as humidity increased from 10% to 89%, a variation comparable to the system's noise floor. This confirms that within this wide operational window, encompassing most laboratory and industrial environments, the sensor's thermal calibration remains robust and effectively independent of humidity. However, the study also clearly defines the sensor's physical limitations. At near-saturation levels (≥ 95% RH), a significant non-thermal angular shift was observed. At 97% RH, the emission angle decreased sharply by 3.28°, 3.20°, 3.97° and 2.2° at 24.60 °C, 32.71 °C, 48.47 °C, and 64.08 °C respectively. This behavior is attributed to the hygroscopic nature of the Polyvinyl Alcohol (PVA) matrix. At extreme humidity, the physical swelling of PVA triggers synergistic effects characterized by a decrease in the effective refractive index (n) due to water absorption ($n_{\mathrm{water}} \approx 1.33 < n_{\mathrm{PVA}}$), and a simultaneous increase in the grating period (Λ) resulting from physical expansion. According to the grating coupling equation ($k_0 \sin\theta_{\mathrm{side}} = k_{\mathrm{SPP}} - 2\pi m/\Lambda$), these combined factors contribute to a reduction in $\sin\theta_{\mathrm{side}}$, thereby explaining the observed angular drop. In conclusion, the sensor is proven to be reliable within the 10–89% RH range, with a clearly identified failure mode only occurring at saturation levels.

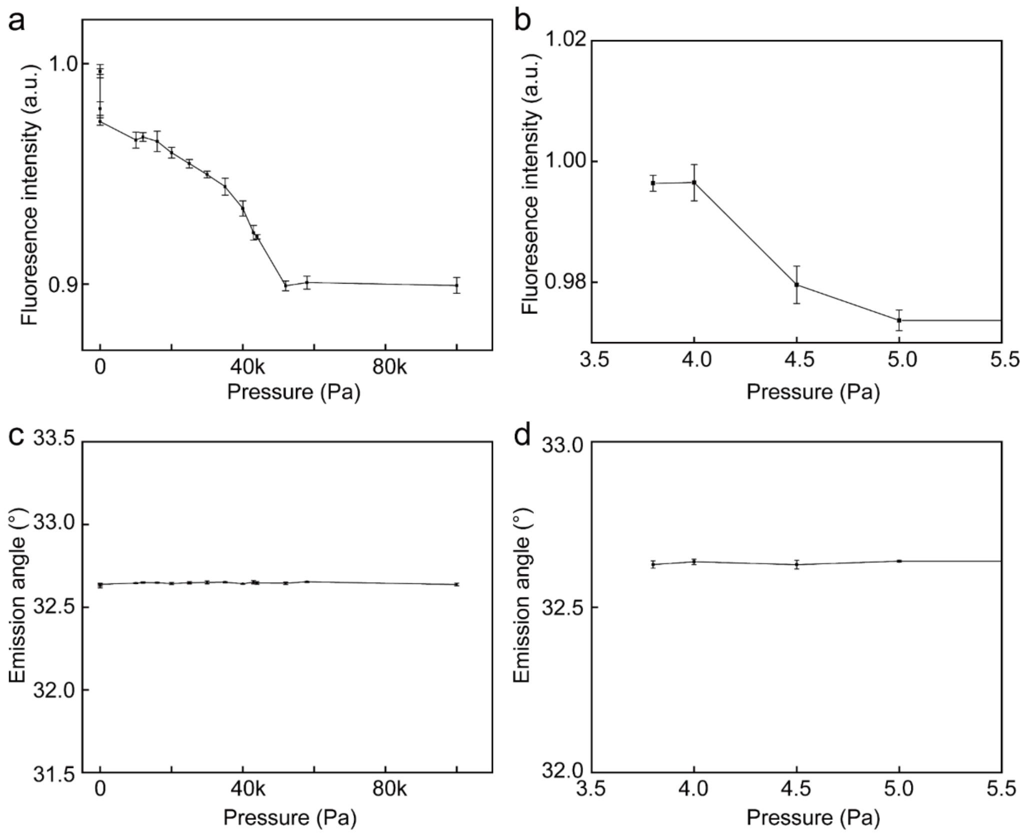

Figure S19. Fluorescence intensity and emission angle response to environmental pressure variations. a, b) Normalized fluorescence intensity of the $[Ru(phen)_3]Cl_2$ sensing layer recorded as a function of environmental air pressure at a constant temperature of 25 °C. a) Displays the full pressure range spanning from high vacuum (3.8 Pa) to ambient atmospheric pressure (100 kPa), while b) provides a magnified view of the low-pressure region between 3.5 Pa and 5.5 Pa. The fluorescence intensity exhibits a segmented attenuation of approximately 10% as the pressure increases to atmospheric levels, attributed to oxygen quenching. c, d) Corresponding fluorescence emission angle measured simultaneously across the identical pressure range, with d) highlighting the magnified low-pressure regime. The emission angle varies between 32.62° and 32.65° throughout the pressure loading process, yielding a standard deviation of 0.003°. Data are presented as mean SD (n = 3 independent measurements).

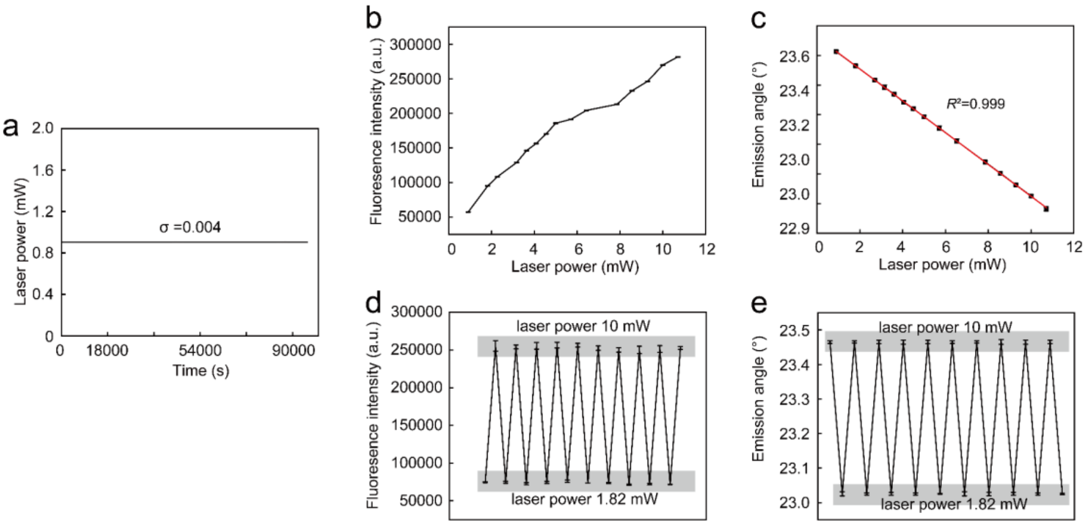

Figure S20. Verification of sensor insensitivity to excitation power fluctuations and decoupling of thermal signals. a) Long-term power stability test of the 473 nm excitation laser over 25 hours (90000 s), showing a minimal variation of 0.470% (σ = 0.004 mW) at the operating current. Static response of b) fluorescence intensity and c) emission angle as the laser power increases from 0.91 mW to 10.71 mW. Fluorescence intensity exhibits a nonlinear, direct dependence on photon flux, whereas the emission angle shows a linear decrease ($R^2 > 0.999$), confirming that the angular signal tracks the laser-induced physical temperature rise (ΔT) rather than the optical power itself. d, e) Dynamic response under periodic power switching between 1.82 mW and 10 mW. d) Fluorescence intensity fluctuates between approximately 74000 and 255000 a.u. (intensity ratio > 3.4), accompanied by a large standard deviation (reaching ~6980 a.u.). e) In contrast, the emission angle transitions stably between two distinct thermal equilibrium states with a standard deviation ≤ 0.007°, demonstrating robust decoupling from source power instability. Error bars in b), d) and e) indicate the standard deviation (SD) of 3 independent measurements.

The experimental results demonstrate a fundamental physical distinction: fluorescence intensity exhibits a direct, nonlinear dependence on photon flux, whereas the emission angle responds exclusively to the steady-state thermal rise (ΔT) induced by laser self-heating on the 1-mm-thick quartz substrate. This distinction was verified through both static and dynamic characterization.

To validate the sensor's insensitivity to non-thermal power variations, we quantitatively isolated the dependencies of the intensity and angular signals on excitation power through comprehensive stability, static, and dynamic characterization. The investigation began with an assessment of the intrinsic source stability. A long-term monitoring test of the 473 nm excitation laser over a 25-hour duration (Fig. S20a) confirmed a minimal power variation of 0.470% (σ = 0.004 mW) at the constant laser power of 0.9 mW. Based on the photothermal coefficient determined in subsequent static tests ($k \approx 0.093$ °C/mW), the temperature error induced by this intrinsic power fluctuation ($\Delta P \approx \pm 0.0042$) is calculated to be approximately 0.0004 °C. This error magnitude is approximately 25 times lower than the sensor's reported resolution of 0.01 °C, establishing a robust baseline for high-precision measurements.

Building on this stability baseline, the fundamental physical distinction between intensity and angular responses was verified under static power modulation. As the laser power was incrementally increased from 0.91 mW to 10.71 mW, the fluorescence intensity (Fig. S20b) displayed a nonlinear dependence on photon flux. In contrast, the emission angle (Fig. S20c) exhibited a linear decrease ($R^2 > 0.999$). Using the established temperature calibration curve, this angular shift corresponds to a linear

physical temperature rise of approximately 0.92 °C (from 24.65 °C to 25.57 °C), confirming that the angular signal transduces the laser-induced thermal effect rather than tracking the optical power directly.

Finally, dynamic switching tests between 1.82 mW and 10 mW revealed a divergence in signal stability under power modulation. The intensity signal (Fig. S20d) exhibited severe oscillations between approximately 74000 and 255000 a.u. (amplitude ratio > 3.4), accompanied by a large standard deviation (reaching ~6980 a.u.). Conversely, the angular signal (Fig. S20e) transitioned cleanly between two stable equilibrium states corresponding to the distinct thermal setpoints, maintaining a standard deviation ≤ 0.007°. These findings collectively confirm that the angle-based paradigm effectively isolates thermal measurement from optical power fluctuations. Unlike intensity-based methods, the angular signal responds linearly to physical temperature changes, and errors induced by source instability are negligible within the sensor's operational resolution.

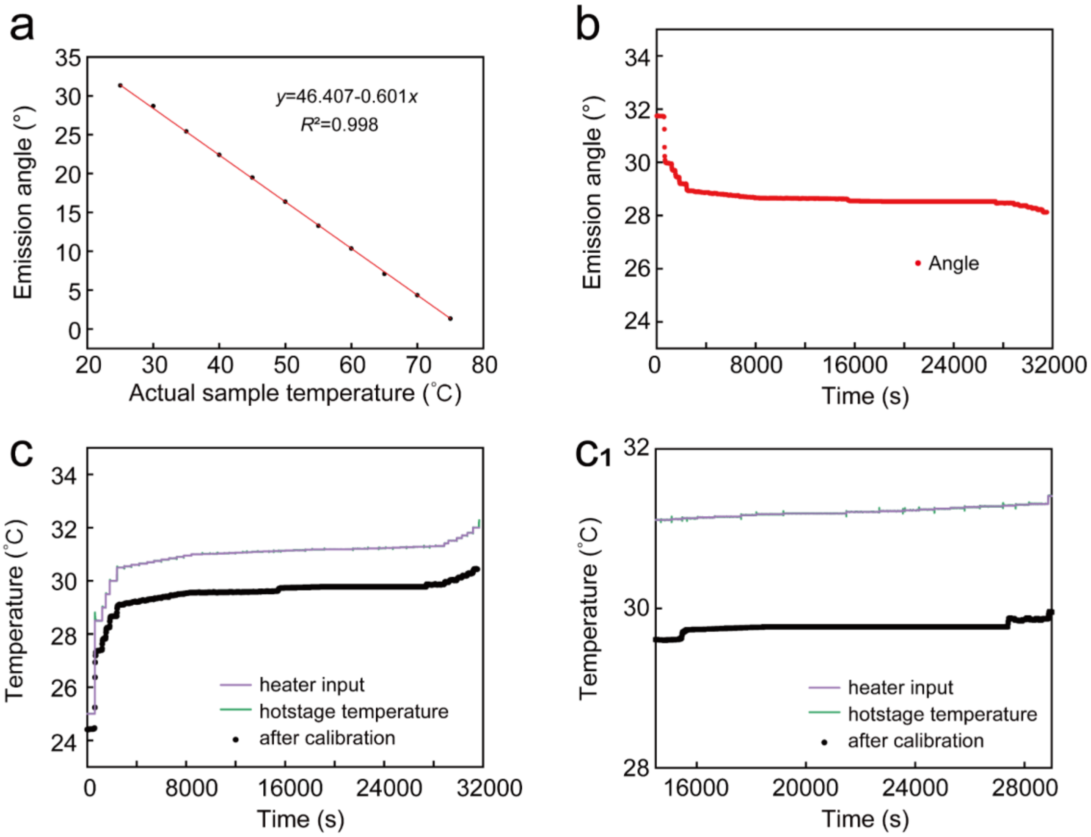

Figure S21. High-fidelity monitoring of the gallium phase transition using the micro-spot angle-based sensor. a) Calibration curve for the 66 μm excitation spot configuration (20× objective), showing a linear response ($R^2 = 0.998$) with a sensitivity derived for micro-area measurements. b) Temporal evolution of the raw fluorescence emission angle recorded during the controlled heating process of the gallium sample. c) Comparison of the thermal profiles over time. The grey line represents the linear temperature ramp of the external heating stage, while the black points represent the actual sample temperature measured by the sensor. c_1) Magnified view of the phase transition region from c). The external heating stage was programmed with a slow, controlled ramp rate of 0.1 °C/min to prevent thermal overshoot and accurately resolve the latent heat plateau. The sensor resolves a distinct isothermal

plateau spanning from 15679 s to 27387 s, with the measured temperature stabilized between 29.73 °C and 29.77 °C, precisely matching the theoretical melting point of gallium (29.76 °C) despite the continuous increase in external heating.

The sensor successfully resolved the thermodynamic signature of the phase transition with high fidelity. As shown in the raw angular data (Fig. S21b), the emission angle exhibits a distinct stabilization period corresponding to the melting process. The converted temperature profile (Fig. S21c) reveals a critical physical phenomenon: while the external heat input (hot stage setpoint, grey line) continued to rise linearly, the local sample temperature (black points) was thermodynamically clamped at the melting point due to the latent heat of fusion. The magnified view (Fig. S21c_1) highlights this isothermal plateau, spanning from t = 15679 s to 27387 s. During this ~3.2-hour phase transition window, the measured temperature remained stable between 29.73 °C and 29.77 °C. This measured range is in agreement with the theoretical melting point of gallium (29.76 °C), confirming the sensor's capability to perform non-contact, micro-scale thermal analysis with an absolute accuracy better than 0.04 °C and a resolution sufficient to resolve the latent heat plateau.

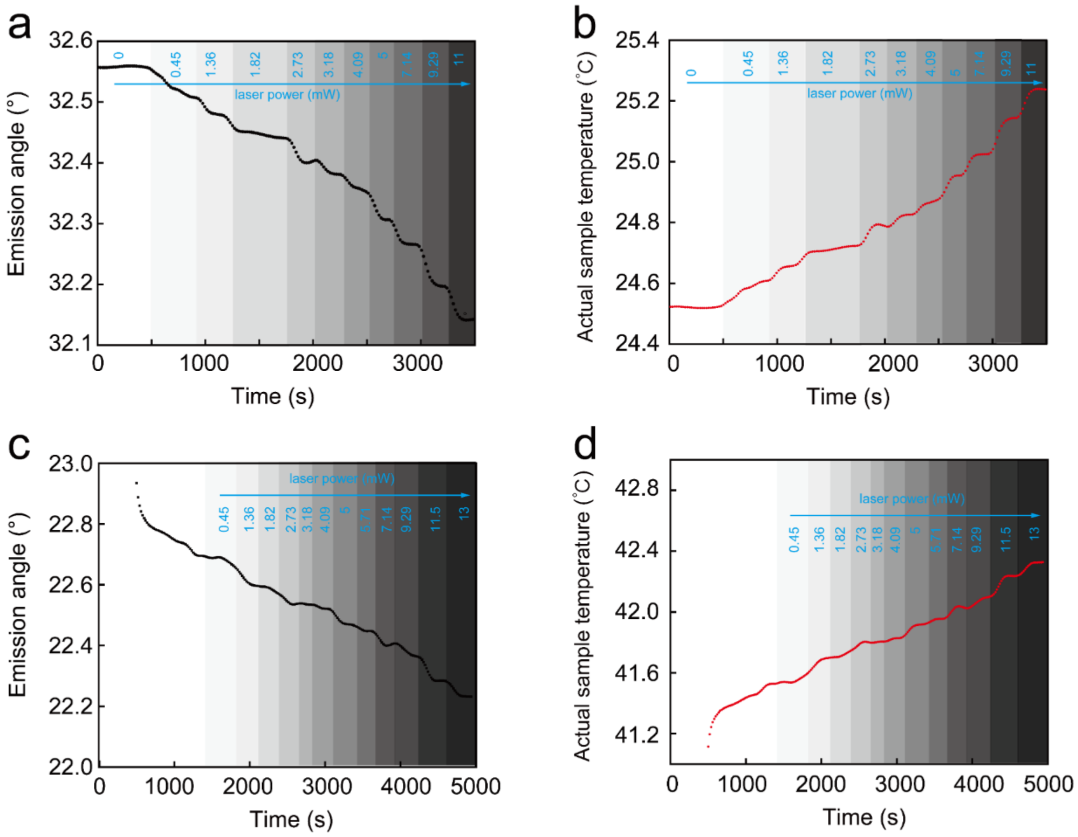

Figure S22. Quantitative characterization of laser-induced self-heating effects. a, b) Photothermal response under ambient conditions (heating stage deactivated). a) Stepwise decrease in fluorescence emission angle and b) corresponding rise in calibrated actual sample temperature as the excitation laser power is incrementally increased from 0 to 11 mW. The background shading gradient (white to dark grey) visually represents the increasing laser power density. c, d) Photothermal response under active thermal control (heating stage setpoint maintained at 50 °C). c) Emission angle and d) calibrated actual temperature evolution as laser power is ramped from 0.45 mW to 13 mW. In both regimes, the sensor resolves a linear temperature rise (~0.07–0.08 °C/mW) induced by the optical energy injection, demonstrating its capability for high-precision micro-calorimetry.

To quantitatively characterize the micro-scale photothermal effect induced by the excitation source itself, a variable-power laser heating experiment was conducted. The sensor was mounted on a temperature-controlled stage, and a 473 nm excitation laser beam (~2 mm diameter) was employed simultaneously as the fluorescence excitation source and the heat injection source. The laser output power was precisely modulated using a variable neutral density filter and monitored by a power meter. To evaluate the system under different regimes, two distinct thermal boundary conditions were investigated, comprising a passive ambient condition where the heating stage was deactivated and an active thermal equilibrium condition with the stage setpoint maintained at 50 °C. In both regimes, the laser power was incrementally increased in a stepwise manner (from ~0 mW to >10 mW). At each power step, the system was allowed to reach a steady-state thermal equilibrium, after which the θ_{side} was recorded. The corresponding T_{actual} was then derived using the pre-established calibration equation ($\theta_{\mathrm{side}} = -0.580 \times T_{\mathrm{actual}} + 46.779$).

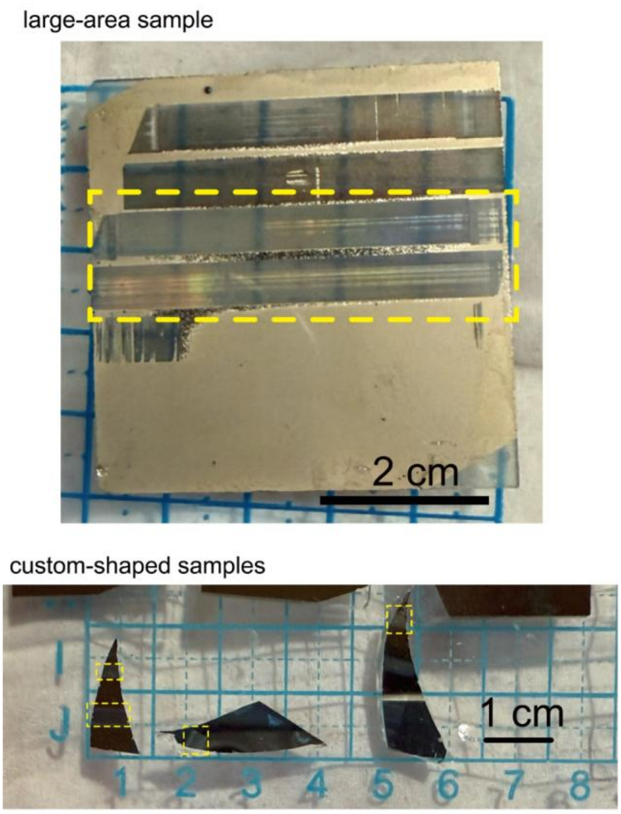

Figure S23. Macroscopic morphology of the sensor samples. Femtosecond laser-fabricated large-area sample (top) and various custom-shaped samples (bottom). Sensing regions are highlighted in yellow.

Supplementary Note 2

Optical simulation details

Three-dimensional Finite-Difference Time-Domain (3D-FDTD) simulations (Lumerical) were performed to model the optical response of the patterned substrates. A multi-step modeling strategy was employed to ensure systematic and reproducible results. First, a parameterized base model was established, which then served as the foundation for more complex derivative structures.

Base model construction

The structural geometry was generated using script-based parameterization to ensure systematic control over the topographical details. The fundamental device architecture comprises a silicon dioxide substrate coated with a 50-nm-thick gold film. The surface topography is defined by analytical functions governing variables such as the central radius, periodicity, duty cycle, and structural height. Specifically, the CCG model was established within a 16 × 16 μm^2 physical area, characterized by symmetry along the *X* and *Y* axes. This configuration features a curvilinear grating profile formed on the gold layer, which is geometrically approximated as a semi-cylinder. The effective height of this feature is modulated by adjusting the intersection depth between the semi-cylindrical element and the underlying gold base. The primary CCG unit is defined by a 500 nm period, a 0.5 duty cycle, and a vertical height of 50 nm.

Simulation domain and boundary conditions

Computational efficiency was optimized by exploiting the inherent structural symmetry, which allowed the simulation volume to be reduced to one-quarter of the physical system. The resulting computational domain spanned 12 × 12 × 6 μm^3. The excitation source consisted of a *Z*-oriented electric dipole positioned at the structural origin, producing electromagnetic fields symmetric relative to the x=0 and y=0 planes. Consequently, symmetric boundary conditions were imposed on these internal planes. To simulate an infinite free-space environment and suppress non-physical reflections, Perfectly Matched Layers (PML) were applied at all external boundaries (*x*-max, *y*-max, *z*-min, and *z*-max). The emission center wavelength was fixed at 580 nm to align with the experimental fluorophore spectral characteristics. Spatial discretization was

managed through an adaptive non-uniform meshing strategy. To guarantee convergence, a refined mesh override with a minimum step size of 5 nm was assigned to critical regions exhibiting high field gradients, specifically around the metallic interfaces and grating edges.

Definition of key optical metrics

Optical performance was quantified through the analysis of specific near-field and far-field indicators. Near-field characterization focused on the electric field magnitude, denoted as $|E|$. To evaluate spatial uniformity, the normalized electric field magnitude (a.u.) was employed, where field distributions within designated 2D cross-sections were scaled to a range of 0 to 1 relative to the global maximum within the plane. Concurrently, to assess the local field concentration capability, the maximum electric field magnitude (a.u.) was extracted from the nanostructure tip. Since the simulation source amplitude is unitary ($|E_{\mathrm{inc}}| = 1$), this raw simulation metric effectively quantifies the local field enhancement factor ($|E|/|E_{\mathrm{inc}}|$), serving as a direct indicator of the localized field strength relative to the incident excitation. Far-field properties were derived using near-to-far-field transformation (NFFT) algorithms. The far-field diffraction power describes the total energy radiated into a specific solid angle. Finally, the primary emission angle was determined by identifying the polar angle corresponding to the maximum power intensity within the angular distribution of the scattered field.

Additional Characterization

Internal thermal gradient simulation

A steady-state heat transfer model was constructed using COMSOL Multiphysics software. The model geometry precisely replicated the sensor's multilayer stack: a 1-mm-thick quartz substrate ($\kappa \approx 1.4$ W/(m·K)), a 150 nm gold grating layer, a 20 nm SiC dielectric layer, and a 100 nm PVA fluorescent layer. A 2D axisymmetric domain with a width of 250 nm (half-period) was employed. The bottom boundary was set to a fixed temperature of 95 °C to simulate the heating stage, while a convective heat flux boundary condition was applied to the top PVA surface to simulate natural cooling at room temperature (25 °C). Periodic boundary conditions were applied to the lateral sides. To ensure computational accuracy, the mesh was locally refined, utilizing an "extremely fine" free triangular mesh for the nano-layers with a maximum element size of 2 nm at interfaces.

Characterization of power fluctuation immunity

To quantitatively characterize the decoupling of the angular sensing signal from excitation power fluctuations, a comprehensive series of stability and power-dependence assessments were conducted using the 473 nm excitation source. First, the intrinsic stability of the laser output was evaluated by continuously monitoring the power variation over a 25-hour period (90000 s) at a constant operating current of approximately 0.9 mW. Following this, the static dependence of the sensor response on incident photon flux was investigated by incrementally increasing the laser power from 0.9 mW to 10.71 mW. At each power step, both the fluorescence intensity and the

emission angle were recorded to correlate the signal response with the laser-induced physical temperature rise. To further assess signal robustness under abrupt power variations, a dynamic switching protocol was implemented wherein the excitation power was periodically modulated between 1.82 mW and 10 mW. During these modulation cycles, the fluorescence intensity and emission angle were continuously tracked to compare the signal variance and equilibrium stability of the two detection modalities.

Micro-scale phase transition monitoring

A high-purity (99.99%) gallium micro-droplet (~1.5 mm diameter) was deposited onto the sensor surface. To achieve high spatial resolution and minimize thermal intrusion, the excitation beam (473 nm) was focused by a $20\times$ objective (NA=0.8) to a diameter of approximately 66 μm, probing the sensor region directly adjacent to the Ga interface. The system was subjected to a precise dual-rate heating protocol: a fast scan (0.05 °C steps) followed by a fine scan (0.01 °C steps) near the melting point. The angular signal collected from the 66 μm micro-spot was converted to absolute temperature using a spot-specific calibration curve established in-situ ($T_{\text{actual 66 μm laser}} = 77.2556 - 1.6650 \cdot \theta_{\text{side}}$).

Quantification of laser-induced self-heating effect

To quantitatively characterize the micro-scale photothermal effect induced by the excitation source itself, a variable-power laser heating experiment was conducted. The sensor was mounted on a temperature-controlled stage, and a 473 nm excitation laser

beam (~2 mm diameter) was employed simultaneously as the fluorescence excitation source and the heat injection source. The laser output power was precisely modulated using a variable neutral density filter and monitored by a power meter. To evaluate the system under different regimes, two distinct thermal boundary conditions were investigated, comprising a passive ambient condition where the heating stage was deactivated and an active thermal equilibrium condition with the stage setpoint maintained at 50 °C. In both regimes, the laser power was incrementally increased in a stepwise manner (from ~0 mW to >10 mW). At each power step, the system was allowed to reach a steady-state thermal equilibrium, after which the θ_{side} was recorded. The corresponding T_{actual} was then derived using the pre-established calibration equation ($\theta_{\mathrm{side}} = -0.6015 \times T_{\mathrm{actual}} + 48.438$).

Environmental humidity characterization

To quantitatively assess the influence of environmental relative humidity (RH) on the sensor's performance, the device was housed in a custom-designed, hermetically sealed chamber equipped with internal air circulation to ensure environmental homogeneity. Real-time RH monitoring was performed using a calibrated digital hygrometer. The humidity control protocol was implemented in two sequential phases. Initially, the chamber was desiccated to a baseline of approximately 12% RH using a saturated Lithium Chloride (LiCl) solution. Subsequently, an atomizing device was employed to inject controlled moisture pulses, incrementally elevating the RH to specific setpoints of 10%, 24%, 33%, 45%, 51%, 60%, 72%, 81%, 89%, 95%, and 97%. Measurements were conducted at four constant, calibrated temperatures (T_{actual} = 24.60 °C, 32.71 °C,

48.47 °C, and 64.08 °C). At each humidity plateau, the system was allowed to stabilize prior to recording the fluorescence emission angle (averaged over five measurements). Data at 100% RH were excluded to prevent measurement artifacts arising from surface condensation.

Pressure-dependent characterization

To evaluate the sensor's stability against oxygen quenching, pressure-dependent fluorescence measurements were conducted in a vacuum chamber equipped with optical windows. The sample was placed within the chamber, and the internal pressure was precisely regulated from a high-vacuum state (3.8 Pa) to ambient atmospheric pressure (100 kPa) using a vacuum pump system. At each pressure node, the system was maintained at 25 °C for at least 5 minutes to ensure thermodynamic equilibrium and gas diffusion stability before spectral acquisition. The optical collection setup remained identical to that used for the angle-resolved measurements.

Accelerated thermal cycling

The sensor was subjected to 1000 consecutive thermal cycles in a standard laboratory environment (60% RH). The temperature was modulated using a high-precision stage between setpoints of 25 °C and 75 °C. During each cycle, the system was held at the thermal extremes to ensure equilibrium. The θ_{side} was continuously monitored and subsequently converted into the T_{actual} using the established calibration equation ($T_{\text{actual}} = 80.653 - 1.724 \times \theta_{\text{side}}$). To ensure data fidelity and eliminate random

noise, thermal equilibrium was maintained at each temperature setpoint prior to acquisition. Each reported angular data point represents the arithmetic mean of 5 consecutive spectral measurements.

Characterization of spatial resolution limit

To quantify the physical spatial resolution limit of the sensor platform (based on a 1-mm-thick JGS1 quartz substrate), a thermal point spread function (PSF) characterization was performed using a laser-induced micro-heating method. Distinct from the 473 nm fluorescence excitation source, a separate 532 nm continuous-wave laser was employed specifically as a localized heating probe. This beam was focused onto the gold grating sensing region via a $20\times$ microscope objective (NA = 0.8). To ensure interaction with a sufficient number of grating periods for effective coupling while maintaining a point-like heat source relative to the substrate diffusion, the beam was slightly defocused to generate an effective excitation spot diameter of approximately 66 μm. The resulting thermal diffusion profile across the quartz substrate was directly imaged using a high-precision cooled infrared thermal imager (MAG-F7H). The physical spatial resolution is defined as the full width at half maximum (FWHM) of the measured thermal spot.

Reference

1. Juris, A. *et al.* Ru(II) polypyridine complexes: photophysics, photochemistry, electrochemistry, and chemiluminescence. *Coord. Chem. Rev.* **84**, 85–277 (1988).

2. Phan Huu, D. K. A. *et al.* Thermally activated delayed fluorescence: polarity, rigidity, and disorder in condensed phases. *J. Am. Chem. Soc.* **144**, 15211–15222 (2022).

3. Khurgin, J. B. How to deal with the loss in plasmonics and metamaterials. *Nat. Nanotechnol.* **10**, 2–6 (2015).

4. Lucarini, V., Saarinen, J. J., Peiponen, K.-E. & Vartiainen, E. M. Kramers-Kronig relations in optical materials research. *Springer Ser. Opt. Sci.* **110**, 1–161 (2005).
