# Supplementary material for: Angle-based fluorescence thermometry with high sensitivity and high resolution
Source: Nat Commun. 2026 May 22;17:6723. doi: 10.1038/s41467-026-73430-4 (PMC13385821; doi:10.1038/s41467-026-73430-4)
Supplement: Supplementary file 2 — Transparent Peer Review file [file 41467_2026_73430_MOESM2_ESM.pdf]

nature portfolio

Peer Review File

Angle-based fluorescence thermometry with high sensitivity and high resolution

Corresponding Author: Professor Min Wang

This file contains all reviewer reports in order by version, followed by all author rebuttals in order by version.

Version 0:

Reviewer comments:

Reviewer #1

(Remarks to the Author)
This work titled “Angle-based fluorescence thermometry with high sensitivity and high resolution” is a demonstration with major flaws. The design and characterization show some feasibility of a plasmonic quenching-based temperature response; however, the preliminary data are far from demonstrating a working thermometer and have obviously overlooked key thermometry design strategies and operating principles. Therefore, this reviewer recommends a rejection of this work. Some major concerns are listed below:
1. Design and working principle of thermometer. In the introduction section Page 2 line 45, the authors described that “the fluorescence intensity variation method … is, however, highly sensitive to non-thermal perturbations such as excitation-power fluctuations, fluorophore-concentration changes and environmental factors…” However, this work also introduced an intensity-based method, which is still vulnerable to excitation-power fluctuations during angle movement. To make things worse, from Figure 1 and Figure S1, the measurement needs a carefully aligned optical path and rotating of the “sensor”. The measurement of non-movable objects seems impossible, otherwise a very complex optical path would need to be designed. In addition, the operation of this sensor requires an unobstructed excitation and collection path, which further reduces the applicable scenarios.
2. Repeatability and reliability of the thermometer. The temperature dependence of the fluorescence angle originates from “alterations in the local structure of Ru(phen)3Cl2 or its interactions with the environment upon heating” (Page 15 Line 309). Because the authors evaluated the sensor only in air of unknown humidity, whether environmental conditions affect the calibration curve remains unclear. Figure 4b further shows noticeable decrease in all the temperature curves, which might cause several degrees of measurement error. The authors also fail to demonstrate any cycles at known temperatures.
3. Spatial resolution of this thermometer. The spatial resolution of this thermometer is not demonstrated and highly questionable. From Page 19, Line 405, the authors made a small sensor of 2.5 mm*2.5 mm, which is bigger than a thermocouple tip. Presumably, at least several periods of grating need to be present for a reasonable response (also not discussed in the manuscript). Additionally, the thermal conductivity of SiO_2 is not good.
In summary, although the idea of angle-resolved thermometry combing plasmonic structure and temperature response seems interesting, neither the science nor the feasibility was solidly demonstrated in this work.

Reviewer #2

(Remarks to the Author)
In this work, the authors report a novel approach to thermal sensing using a fluorescence signal. The proposed idea involves combining a gold nanograting with a fluorescent material that is coupled to the grating as a covering film. Throughout the manuscript, the authors claim the existence of a cascade effect that results in a strong temperature dependence of the diffraction angle (superimposed on the emission spectra), which can be used to monitor the temperature of the grating+dye structure.

The field of fluorescence sensing is developing rapidly, especially in applications targeting thermal sensing. Typically, the approach involves the use of a luminescent nanostructure, and only a few alternative methods are reported. This work is novel in that it proposes an alternative way of measuring temperature. In this sense, I believe the paper could be of interest to the scientific community. Nevertheless, I find that the paper presents some issues and drawbacks:

1. After reading several times, the explanation of the "cascade" effect leading to a diffraction angle strongly dependent on temperature remains unclear to me. I believe the authors should find a clearer way to explain this phenomenon. In the end, it seems that the dominant effect is the change in the refractive index of the fluorescent layer with temperature, which affects the resonance condition, right? There are some repeated sentences in the manuscript, and some terms, such as SPP, are not defined (if I am not mistaken).

2. It is not clear how the temperature is determined. Is it necessary to measure the entire intensity vs. angle curve for each measurement? How is this done in real experiments? What is the time required for these measurements? Although the authors report very high sensitivities, in order to compare with other systems, the measurement time must be provided.

3. If I am not wrong, the diffraction angle depends on both the temperature of the gold pattern and the luminescent layer. However, these materials have very different thermal conductivities. Is the temperature homogeneous across the structure? If not, how accurate is the thermal output?

4. What spatial resolution can be achieved with this approach?

5. How relevant are external conditions? This question relates not only to the existence of cross-sensitivity (which should be evaluated, for instance, by checking calibration under different environmental conditions such as humidity or the presence of dust) but also to the role played by ambient temperature. What temperature is actually being measured in their applications? Is it the surface temperature of, for example, batteries, or the temperature of the air in contact with the battery? Note that the latter could be strongly dependent on the thickness of the sensor.

6. Have the authors checked for self-heating effects due to light absorption by gold?

Version 1:

Reviewer comments:

Reviewer #1

(Remarks to the Author)
This reviewer acknowledges the substantial expansion of the demonstration of the sensor's reliability, which arises from the comments of both reviewers. The working principle has been solidified by the authors, especially by introducing the Fourier plane imaging capability. It seems solid in theory and might improve the feasibility of the methodology; however, this capability might still remain unexplored at this stage.
Further questions regarding the working molecules: 1. currently, Ru(phen)3Cl2 molecules are used. It is known for oxygen quenching. A PVA polymer film might be sufficient for resistance to oxygen quenching, but further experiments might need to be carried out to confirm this. 2. Does this methodology apply to all MLCT fluorophores, or all fluorophores that are stable as long as they are in the designed wavelength (and coupled to a certain wavelength of the plasmon structure)? If the fluorophore coating is modified, or another material is applied (which has a similar structure or luminescence), how would the response curve be changed?
The methodology involving moving parts: It is still confusing regarding the mention of rotating turbine blades, high-speed hard disk platters, or centrifuge components. How is this going to work? How is the system mechanically stable if a grating is attached? From Figure S17, the current emission probe decomposed at around 100 degrees Celsius, which suggests that the authors should consider the temperature range as well.
Regarding the spatial resolution (Comment 3), the current demonstration could be potentially misleading. In thermometry, the physical dimension of the sensor is critical because a thermal equilibrium must be established between the measured object and the thermometer. This is precisely why nanothermometers are generally preferred for high-accuracy measurements at small scales, as they minimize thermal perturbation. We acknowledge that the authors carried out rigorous in-situ temperature calibration (Figure S9) to account for the thermal resistance of the materials used. However, a fundamental limitation remains: if the target object has a smaller heat capacity than the micro-sized grating sensor, the sensor will act as a heat sink. Consequently, the measured value will represent the equilibrium temperature between the object and the sensor, rather than the intrinsic temperature of the object. Moreover, establishing thermal equilibrium also requires time, which will inevitably impact the response time of the thermometer. This limitation should be explicitly stated in the maintext to prevent any misinterpretation of the achieved spatial resolution.

Reviewer #2

(Remarks to the Author)
Dear Editor
In my understanding authors have considered seriously all my prevous comments and I think they have answered them properly. From my side the paper is ready for publication. I ave to say that the practical applications of this method could be limited but I think the idea behing is novel and could encourage and motivate the researchers in the field of the hot topic of remote thermometry.

Version 2:

Reviewer comments:

Reviewer #1

(Remarks to the Author)
The authors have addressed my comments well with their further demonstrations and revisions. I now recommend this manuscript for publication. Notably, testing fluorescence stability under various pressures and using Rhodamine B as the fluorophore, combined with the expanded discussion, add solid value to the work.

Open Access This Peer Review File is licensed under a Creative Commons Attribution 4.0 International License, which permits use, sharing, adaptation, distribution and reproduction in any medium or format, as long as you give appropriate credit to the original author(s) and the source, provide a link to the Creative Commons license, and indicate if changes were made.
In cases where reviewers are anonymous, credit should be given to 'Anonymous Referee' and the source.
The images or other third party material in this Peer Review File are included in the article's Creative Commons license, unless indicated otherwise in a credit line to the material. If material is not included in the article's Creative Commons license and your intended use is not permitted by statutory regulation or exceeds the permitted use, you will need to obtain permission directly from the copyright holder.
To view a copy of this license, visit https://creativecommons.org/licenses/by/4.0/

Point-by-Point Response to Editor and Reviewers' Comments

(*Black italic: Reviewer's remarks;* Blue type: Our response)

(Green background: Revised descriptions in main text)

Reviewer #1:

This work titled "Angle-based fluorescence thermometry with high sensitivity and high resolution" is a demonstration with major flaws. The design and characterization show some feasibility of a plasmonic quenching-based temperature response; however, the preliminary data are far from demonstrating a working thermometer and have obviously overlooked key thermometry design strategies and operating principles. Therefore, this reviewer recommends a rejection of this work.

Reply to the Reviewer: We thank the reviewer for their critical and insightful assessment of our work. We have taken the comments regarding the "major flaws" and the preliminary nature of the data with the utmost seriousness. These constructive criticisms prompted us to conduct a comprehensive overhaul of our experimental validation and theoretical analysis to strictly define the sensor's capabilities and limitations. In this revised manuscript, we have rigorously quantified the sensor's reliability through extensive aging tests and systematic humidity characterization. Furthermore, we have solidified the working principle by clarifying the physical mechanism and theoretically validating the Fourier plane imaging capability to address the concerns regarding stationary object measurement. To demonstrate high-fidelity resolution, we performed micro-scale phase transition monitoring of Gallium and rigorous Point Spread Function (PSF) characterization. Finally, we eliminated measurement errors by implementing a rigorous in-situ calibration protocol using a co-located Pt RTD, which effectively decouples the intrinsic sensor response from environmental thermal gradients. We believe these extensive new data and analyses solidly demonstrate the science and feasibility of the proposed thermometer.

Some major concerns are listed below:

1. Design and working principle of thermometer. In the introduction section Page 2

line 45, the authors described that "the fluorescence intensity variation method ... is, however, highly sensitive to non-thermal perturbations such as excitation-power fluctuations, fluorophore-concentration changes and environmental factors..." However, this work also introduced an intensity-based method, which is still vulnerable to excitation-power fluctuations during angle movement.

Reply to the Reviewer: We are grateful for the reviewer's detailed scrutiny regarding the sensing principle and application scenarios. We have addressed the concerns regarding power fluctuations, signal decoupling, and measurement configurations through the following comprehensive analysis.

To verify the sensor's immunity to excitation power variations, which is a critical advantage over intensity-based methods, we conducted both static and dynamic power modulation tests. As illustrated in **Figure S18**, while the fluorescence intensity fluctuated drastically (exceeding 340%) under power modulation between 1.82 mW and 10 mW, the emission angle remained exceptionally stable with a standard deviation below 0.007°. It is important to note that the angular data presented in this context corresponds to the physical temperature rise induced by laser self-heating, and the conversion to temperature values was verified using the rigorous calibration curve detailed in our subsequent response to **Comment 2**. Regarding the intensity data presented in the original manuscript, we clarify that it was included solely to contrast its inherent instability with the robustness of the angular signal. To avoid any ambiguity, the revised manuscript now presents normalized intensity data in **Figures 2b-d, 4a, S2, S11a and S11a$_1$**, explicitly stating that intensity is not the sensing parameter in our paradigm.

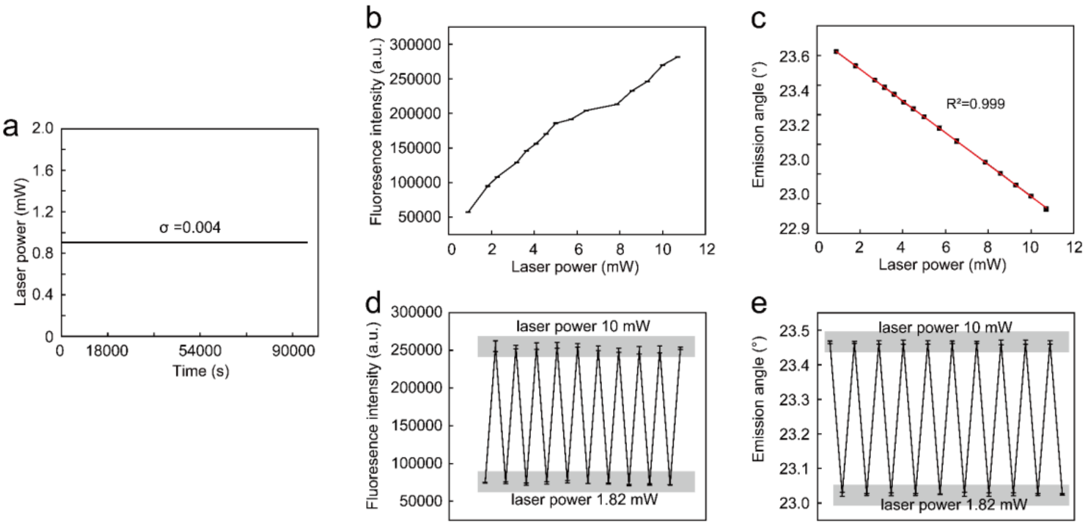

Figure S18. Verification of sensor immunity to excitation power fluctuations and decoupling of thermal signals. a) Long-term power stability test of the 473 nm excitation laser over 25 hours (90000 s), showing a minimal variation of 0.470% ($\sigma =$ 0.004 mW) at the operating current. Static response of b) fluorescence intensity and c) emission angle as the laser power increases from 0.9 mW to 10.71 mW. Fluorescence intensity exhibits a nonlinear, direct dependence on photon flux, whereas the emission angle shows a highly linear decrease ($R^2 =$ 0.999), confirming that the angular signal faithfully tracks the laser-induced physical temperature rise (ΔT) rather than the optical power itself. d, e) Dynamic response under periodic power switching between 1.82 mW and 10 mW. d) Fluorescence intensity fluctuates drastically between approximately 74000 and 255000 a.u. (intensity ratio > 3.4), accompanied by a large standard deviation (reaching ~6980 a.u.). e) In contrast, the emission angle transitions stably between two distinct thermal equilibrium states with a standard deviation $\leq 0.007°$, demonstrating robust decoupling from source power instability.

To make things worse, from Figure 1 and Figure S1, the measurement needs a carefully aligned optical path and rotating of the "sensor". The measurement of non-movable objects seems impossible, otherwise a very complex optical path would need to be designed.

Reply to the Reviewer: The reviewer's observation regarding the optical alignment requirements and the reliance on mechanical rotation in the current experimental setup

is accurate. It is acknowledged that the goniometric configuration used for characterization is less conducive to measuring stationary objects without complex beam steering. However, this limitation is specific to the characterization apparatus employed for high-spectral-resolution validation and does not represent an intrinsic constraint of the angle-based sensing paradigm itself. To address this and demonstrate the broader applicability of the method, two complementary detection schemes are proposed and analyzed.

First, regarding the measurement of stationary objects, the angle-based sensing mechanism is fully compatible with static, high-speed detection via Fourier plane (back focal plane, BFP) imaging. In this optical configuration, the angle-resolved emission is mapped spatially onto a 2D detector array (CCD or CMOS) located at the back focal plane of a collection objective. According to the Abbe sine condition, the diffraction angle (θ_{side}) corresponds directly to a specific radial distance (r) on the detector ($r = f \cdot \mathrm{NA} \cdot \sin\theta_{side}$), effectively eliminating the need for any mechanical motion. This approach, often referred to as Leakage Radiation Microscopy (LRM) or Momentum-space imaging, is well-established for characterizing the angular emission of plasmonic structures and has been successfully implemented in high-precision sensing contexts (Drezet et al. for the foundational principle[1]; Ohad et al. for refractive index sensing applications[2]; Genco et al. for advanced *k*-space hyperspectral imaging[3]). By replacing the single-point detector and rotation stage with a Fourier lens system, the sensor can monitor static targets with high temporal resolution limited only by the detector's frame rate (potentially kHz range), far exceeding the speed of mechanical scanning.

Second, the current rotational scanning configuration offers distinct advantages for specific industrial scenarios where the target object itself undergoes macroscopic motion. In applications such as the thermal monitoring of rotating turbine blades, high-speed hard disk platters, or centrifuge components, the inherent rotation of the object can be leveraged to perform the angular scan naturally. In such cases, a fixed detector can sample the emission as the sensor sweeps through the acceptance angle, simplifying the instrumentation by removing the need for beam steering optics. Consequently, the dynamic rotational scheme and the static Fourier imaging scheme should be viewed as

complementary engineering implementations of the same underlying physical principle, each tailored to distinct measurement constraints—one utilizing object motion for efficient scanning and the other employing optical mapping for static, high-speed monitoring. This versatility underscores the broad potential of the angle-based paradigm across diverse operational environments.

In addition, the operation of this sensor requires an unobstructed excitation and collection path, which further reduces the applicable scenarios.

Reply to the Reviewer: While we acknowledge that the requirement for an unobstructed optical path defines specific use cases, this configuration uniquely positions the sensor as a high-fidelity instrument for non-contact, micro-scale physical characterization. We demonstrated this versatility through two distinct experiments. First, to demonstrate high spatial resolution, we monitored the solid-liquid phase transition of Gallium using an excitation beam focused to a 66 μm micro-spot. Given the change in optical configuration from the standard macro-spot to a micro-spot, a specific in-situ calibration was performed for this setup to ensure maximum fidelity (**Figure S19a**). Using this specific calibration, the sensor successfully resolved the latent heat plateau of Gallium at 29.76 °C with 0.01 °C resolution (**Figure S19**). Second, to quantify the laser-induced self-heating effect, we utilized the standard macro-spot configuration. By applying the standard calibration curve derived in Response to **Comment 2**, we leveraged the sensor's high sensitivity to function as a micro-calorimeter, linearly resolving the minute temperature rise induced by optical absorption (**Figure S20**).

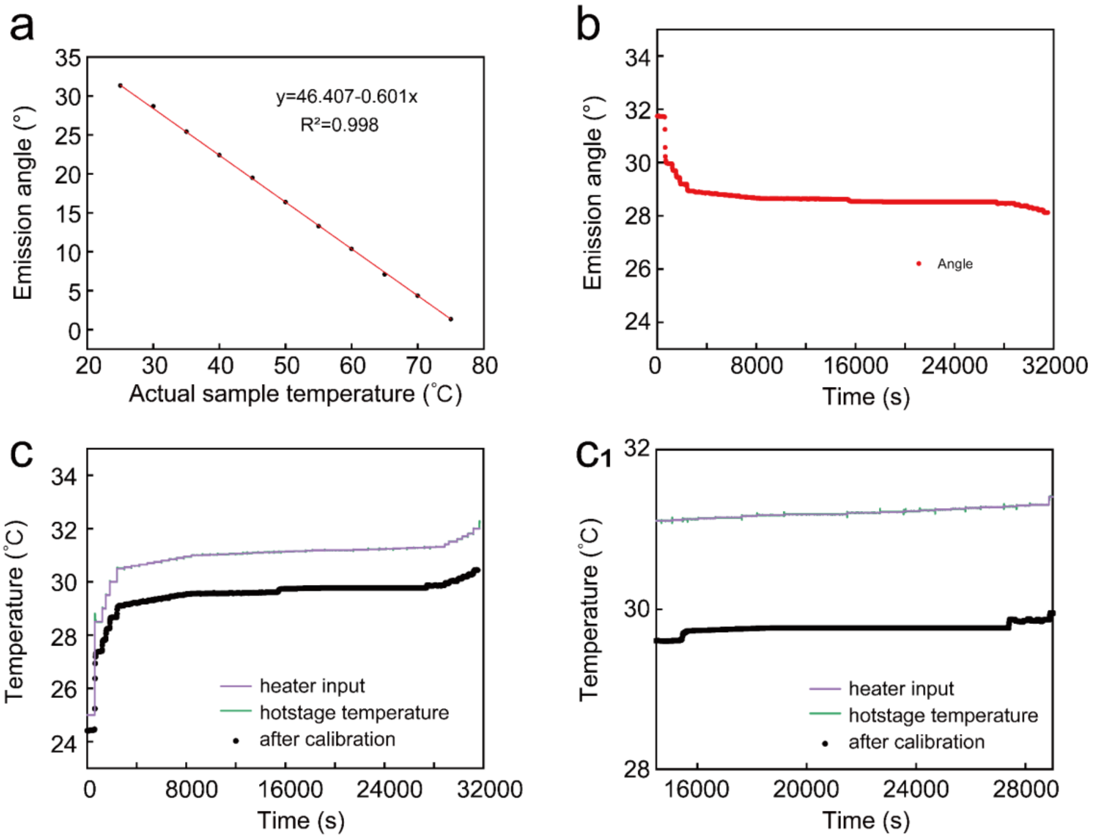

Figure S19. High-fidelity monitoring of the Gallium phase transition using the micro-spot angle-based sensor. a) Calibration curve for the 66 μm excitation spot configuration (20× objective), showing a linear response ($R^2 = 0.998$) with a sensitivity derived for micro-area measurements. b) Temporal evolution of the raw fluorescence emission angle recorded during the controlled heating process of the Gallium sample. c) Comparison of the thermal profiles over time. The grey line represents the linear temperature ramp of the external heating stage, while the black points represent the actual sample temperature measured by the sensor. c_1) Magnified view of the phase transition region from c). The external heating stage was programmed with a slow, controlled ramp rate of 0.1 °C/min to prevent thermal overshoot and accurately resolve the latent heat plateau. The sensor resolves a distinct isothermal plateau spanning from 15679 s to 27387 s, with the measured temperature stabilized between 29.73 °C and 29.77 °C, precisely matching the theoretical melting point of Gallium (29.76 °C) despite the continuous increase in external heating.

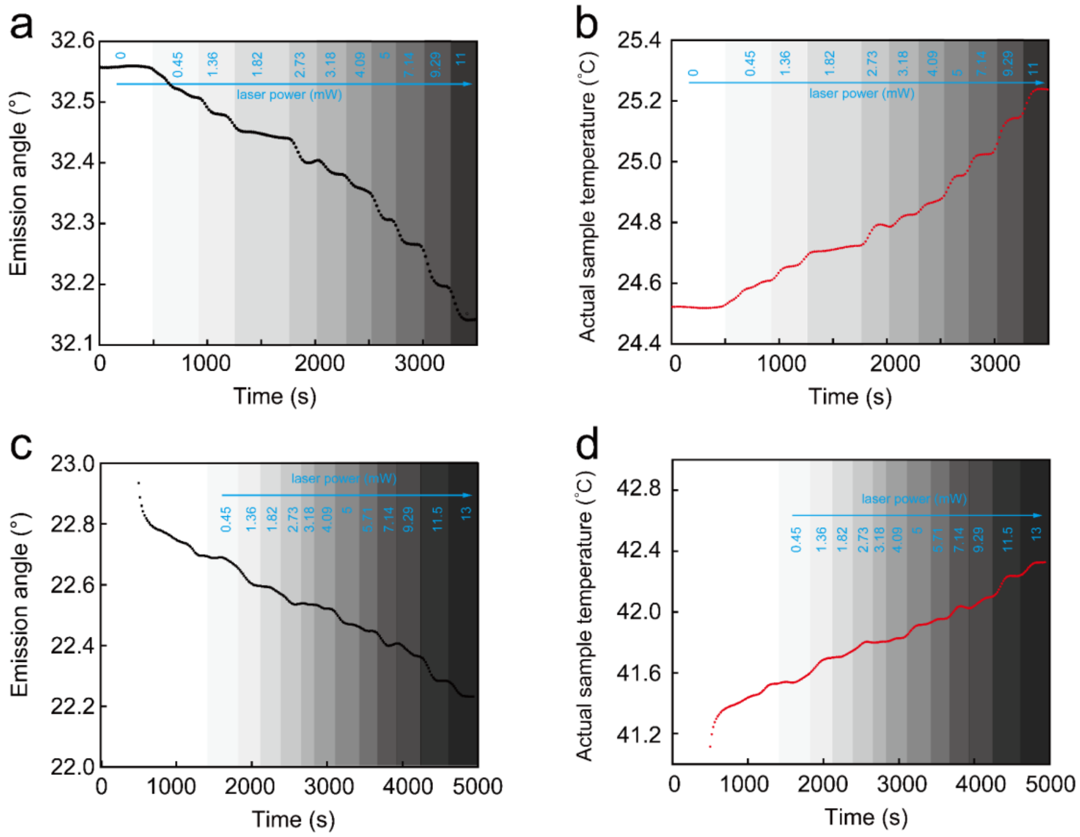

Figure S20. Quantitative characterization of laser-induced self-heating effects. a, b) Photothermal response under ambient conditions (heating stage deactivated). a) Stepwise decrease in fluorescence emission angle and b) corresponding rise in calibrated actual sample temperature as the excitation laser power is incrementally increased from 0 mW to 11 mW. The background shading gradient (white to dark grey) visually represents the increasing laser power density. c, d) Photothermal response under active thermal control (heating stage setpoint maintained at 50 °C). c) Emission angle and d) calibrated actual temperature evolution as laser power is ramped from 0.45 mW to 13 mW. In both regimes, the sensor resolves a linear temperature rise (~0.07-0.08 °C/mW) induced by the optical energy injection, demonstrating its capability for high-precision micro-calorimetry.

2. Repeatability and reliability of the thermometer. The temperature dependence of the fluorescence angle originates from "alterations in the local structure of Ru(phen)$_3$Cl$_2$ or its interactions with the environment upon heating" (Page 15 Line 309).

Reply to the Reviewer: The reviewer's concern regarding the reliability of the temperature calibration and the potential influence of environmental factors is well-founded. We confirm that the "decrease in temperature curves" noted in the original manuscript was indeed attributable to thermal resistance and lag inherent to the experimental setup rather than sensor instability. To address this and eliminate measurement errors, we implemented a rigorous in-situ calibration protocol.

As referenced in our response to the previous comment, we utilized a co-located platinum resistance thermometer (Pt RTD) to measure the actual sample surface temperature (T_{actual}) synchronously with the sensor's angular response. To rigorously account for the minor thermal hysteresis inherent to the heating stage mechanism, the final reported T_{actual} for each calibration point was derived from the arithmetic mean of the temperatures measured during both the heating and cooling phases. As shown in **Figure S9**, obtaining T_{actual} effectively eliminated the thermal lag artifacts observed in the setpoint data. The resulting final calibration curve exhibits a high linearity (R^2 >0.999) and serves as the standard for all macro-scale measurements reported in this work.

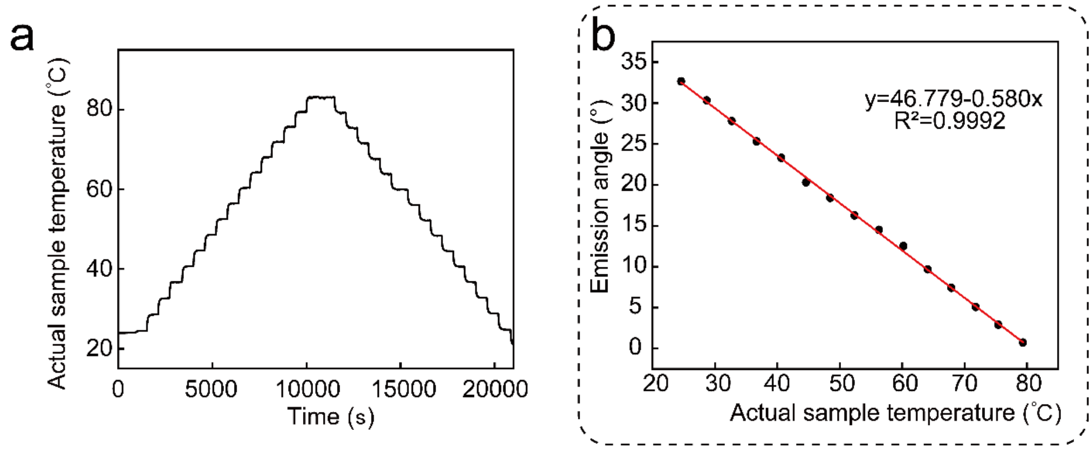

Figure S9. Rigorous in-situ temperature calibration and characterization of thermal resistance. a) Temporal profile of the actual sample surface temperature (T_{actual}) measured by a co-located Pt RTD during a stepped heating and cooling cycle (setpoints: 25 °C to 100 °C). The data reveals the thermal lag and the magnitude of the temperature drop across the substrate. b) The final calibration curve relating the sensor's emission

angle (θ) to the measured actual temperature (T_{actual}). To compensate for the thermal hysteresis observed in panel a, each data point plotted here represents the arithmetic mean of the temperatures measured during the heating and cooling phases. The data shown in this panel (highlighted by the dashed box) is identical to the calibration curve presented in the main text Fig. 4b. The response is highly linear (R^2= 0.9992, sensitivity = 0.580 °/°C), confirming the sensor's accuracy after correcting for the thermal resistance of the experimental setup.

Because the authors evaluated the sensor only in air of unknown humidity, whether environmental conditions affect the calibration curve remains unclear.

Reply to the Reviewer: We acknowledge that the original evaluation lacked specific humidity controls. To quantitatively assess cross-sensitivity and define operational boundaries, we conducted a systematic characterization across a wide relative humidity (RH) range from 10% to 97% at four distinct constant temperatures. As illustrated in **Figure S17**, the sensor demonstrates exceptional stability within the broad range of 10% to 89% RH, where the fluorescence emission angle remains virtually constant. For instance, at 48.47 °C, the angular variation was negligible (comparable to the noise floor) despite humidity increasing from 10% to 89%. This confirms the sensor’s robustness for typical laboratory and industrial environments. We also identified a specific physical failure mode at near-saturation levels (≥ 95% RH), where a sharp decrease in emission angle occurs due to the hygroscopic swelling of the PVA matrix, which alters the refractive index and grating period. This characterization explicitly defines the reliable operational window of the device.

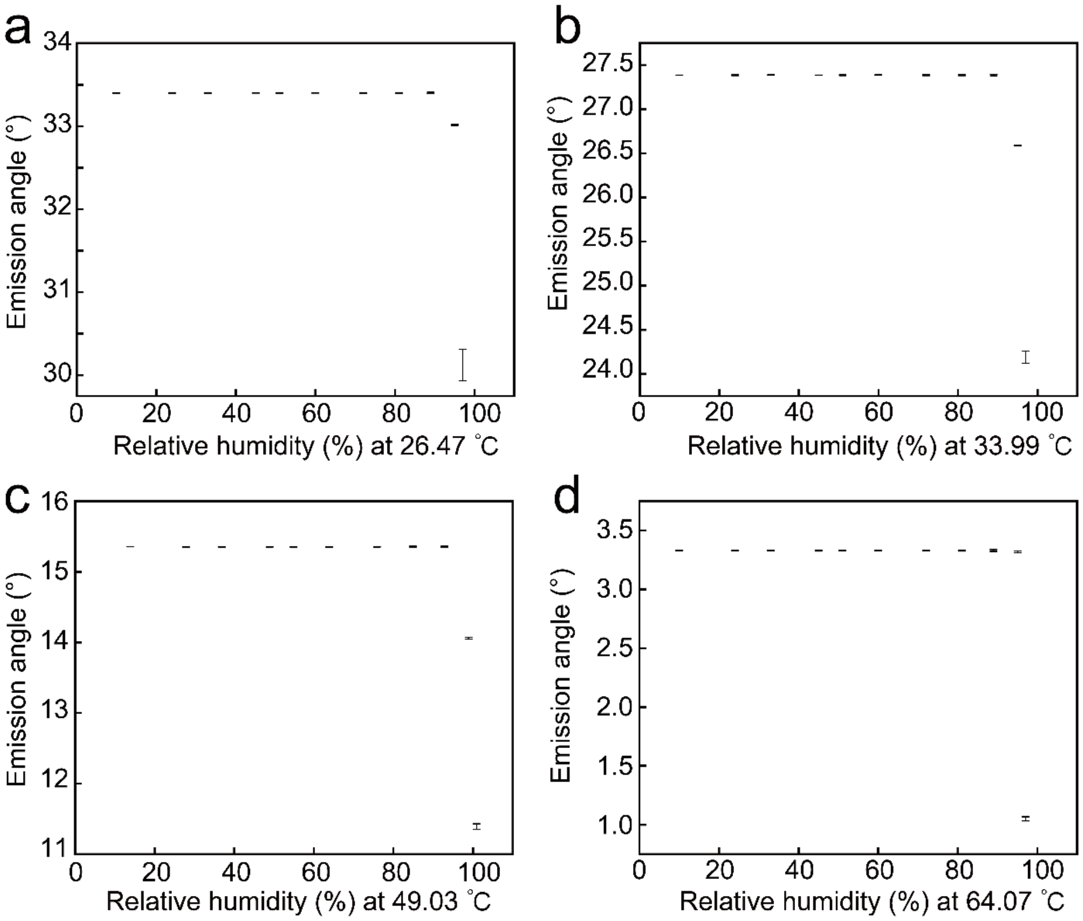

Figure S17. Quantification of environmental humidity influence on sensor performance. a-d) Fluorescence emission angle recorded as a function of relative humidity (RH) ranging from 10% to 97% at four constant, calibrated temperatures: a) 24.60 °C, b) 32.71 °C, c) 48.47 °C, and d) 64.08 °C. To ensure the hygroscopic equilibrium of the PVA sensing layer, the sensor was maintained at each humidity step for 5 minutes prior to data acquisition. The sensor demonstrates exceptional stability with negligible angular drift across the broad operational range of 10%–89% RH. A distinct failure mode is observed only at near-saturation levels (≥ 95% RH), where a sharp decrease in emission angle occurs. This drop is attributed to the hygroscopic swelling of the PVA matrix, which simultaneously reduces the effective refractive index and increases the grating period, physically altering the resonance condition.

Figure 4b further shows noticeable decrease in all the temperature curves, which might cause several degrees of measurement error.

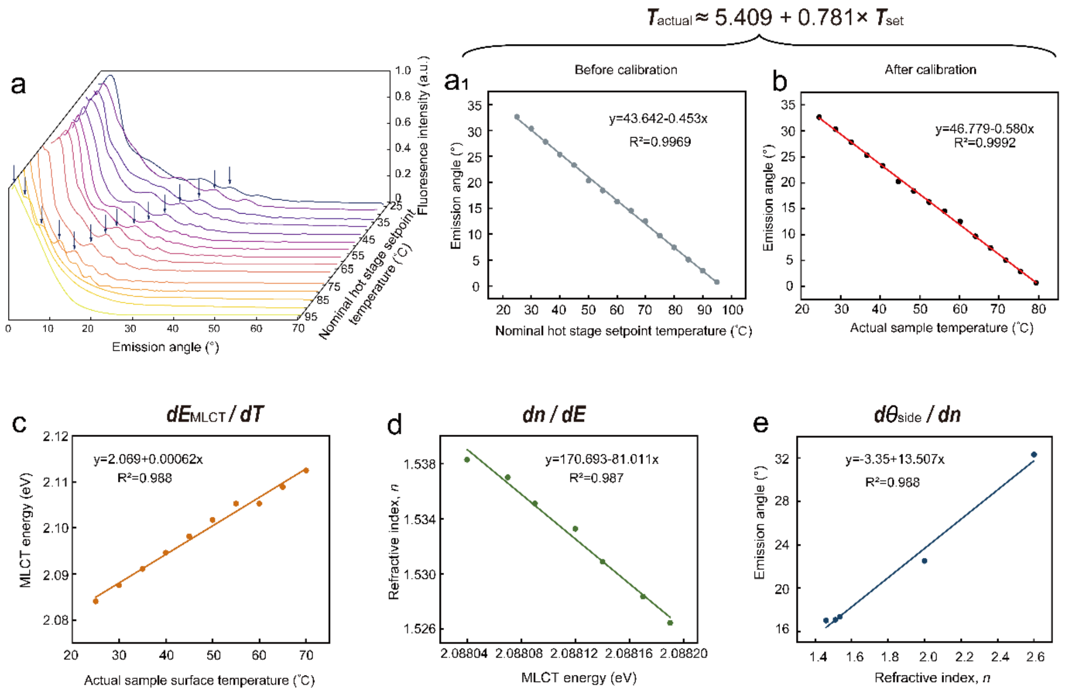

Fig. 4 Sensor performance calibration and mechanistic validation via multi-stage deconstruction. **a** Angle-resolved fluorescence spectra recorded at nominal hot stage setpoints (T_{set}) ranging from 25 °C to 95 °C. The data qualitatively demonstrates the sensing principle: as the temperature increases, the secondary emission angle (θ_{side}, indicated by black arrows) shifts towards smaller angles. **a₁** The dependence of θ_{side} on the nominal setpoint temperature. The relationship between the actual sample temperature (T_{actual}) and the setpoint is quantified as $T_{\text{actual}} = 5.409 + 0.781 \times T_{set}$, reflecting the thermal resistance of the substrate. **b** Sensor calibration curve. The extracted θ_{side} is plotted against the T_{actual}, measured in-situ via a calibrated Pt RTD. This plot represents the final calibration derived from the comprehensive thermal characterization detailed in Fig. S9. The highly linear fit ($R^2 > 0.999$) yields a direct experimental absolute sensitivity (S_{abs}) of 0.580 °/°C. **c-e** Quantitative deconstruction of the sensitivity into three constituent physical mechanisms ($S_{\text{abs}} = dE/dT \times dn/dE \times d\theta_{\text{side}}/dn$).**c** Characterization of Step 1 (Molecular Thermochromism). The MLCT excited-state energy of $Ru(phen)_3Cl_2$ exhibits a linear blueshift of 0.00062

eV/°C with T_{actual} due to thermal modulation of the local dielectric environment. **d** Characterization of Step 2 (Resonant Dispersion). Consistent with Kramers-Kronig causality, spectroscopic ellipsometry confirms a linear correlation between the macroscopic refractive index (n) and the molecular energy (E_{MLCT}), yielding a slope of 81.011 RIU/eV. **e** Characterization of Step 3 (Plasmonic Momentum Matching). The grating structure transduces refractive index (n) changes into large angular (θ_{side}) shifts with a measured gain of 13.507 °/RIU. The product of these three components ($S_{\mathrm{calculated}}$ ≈0.678 °/°C) quantitatively validates the direct measurement in **b**.

Reply to the Reviewer: The reviewer correctly identified that the temperature discrepancies in the original data could lead to measurement errors. We confirm that this phenomenon was attributable to the thermal resistance of the 1-mm-thick quartz substrate and the thermal lag inherent to the previous experimental setup, rather than intrinsic sensor instability. To eliminate these artifacts, we implemented a rigorous in-situ calibration protocol. As detailed in **Figure S9**, we utilized a co-located platinum resistance thermometer (Pt RTD) to measure the actual sample surface temperature (T_{actual}) directly. The calibration data reveals that T_{actual} is consistently lower than the setpoint, quantifying the thermal drop across the substrate. By correlating the emission angle (θ) directly with this calibrated T_{actual}, we established a highly linear response ($R^2 > 0.999$, **Figure 4b**), effectively decoupling the sensor's response from environmental thermal gradients and ensuring measurement accuracy.

The authors also fail to demonstrate any cycles at known temperatures.

Reply to the Reviewer: To rigorously evaluate long-term reliability and fatigue resistance, we conducted an accelerated thermal aging test comprising 1000 continuous cycles. The sensor was cycled between calibrated actual temperatures of 24.91 °C and 63.95 °C in a standard laboratory environment. To ensure the highest fidelity and eliminate random acquisition noise during these measurements, rigorous thermal equilibrium was maintained at each temperature step prior to data collection, and each

data point presented in the cycling trace represents the arithmetic mean of five consecutive spectral scans. As shown in **Figure S16**, the emission angle oscillated consistently throughout the entire duration with no measurable drift in the signal baseline or degradation in response amplitude. A direct comparison between the initial 100 cycles and the final 100 cycles confirms identical thermal profiles. Notably, the standard deviation for these steady-state measurements was calculated to be negligible (< 0.007°), which elucidates the high smoothness of the reported traces. This verifies that the sensor maintains its calibration and structural integrity even after extensive thermal stress. To further standardize these protocols, details regarding the data acquisition (5-scan averaging) and thermal control (equilibrium times, ramp rates) have also been added to the Methods section of the revised manuscript to ensure reproducibility.

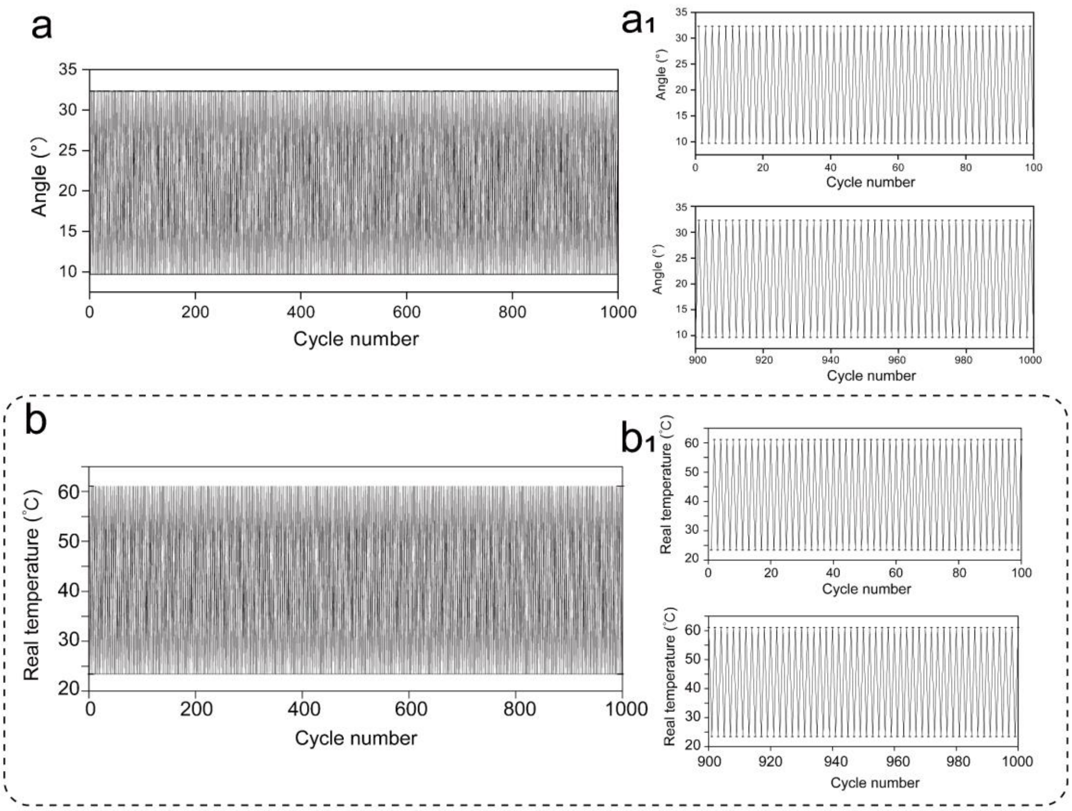

Figure S15. Long-term reliability and repeatability demonstrated via an accelerated 1000-cycle thermal aging test. a) Periodic variations of the fluorescence emission angle over 1000 accelerated thermal aging cycles, demonstrating long-term reliability and

repeatability. To ensure measurement precision and eliminate random noise, the sample was allowed to reach thermal equilibrium at each step, and each plotted point represents the arithmetic mean of five consecutive angular scans. a_1) An expanded view comparing the initial 100 cycles (top) and final 100 cycles (bottom), confirming no measurable drift or degradation in signal baseline or response amplitude. b) The corresponding calibrated actual sample temperature profile derived from the angular data (T_{actual} range: 24.91 °C to 63.95 °C). b_1) Expanded views of the temperature trace for the first 100 cycles (top) and the last 100 cycles (bottom), confirming the sensor's exceptional stability and lack of drift over the extended testing period. Note that the temperature data presented in Figure 5f and $5f_1$ in the main text are reproduced from the dashed box sections of this figure (panels b and b_1, respectively).

*3. Spatial resolution of this thermometer. The spatial resolution of this thermometer is not demonstrated and highly questionable. From Page 19, Line 405, the authors made a small sensor of 2.5 mm*2.5 mm, which is bigger than a thermocouple tip.*

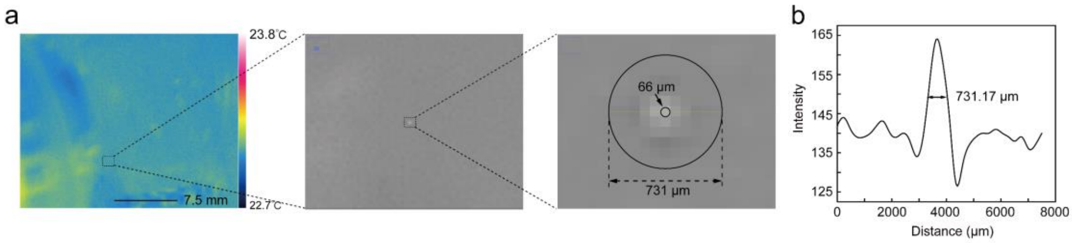

Figure S14. Quantification of the spatial resolution limit via thermal point spread function (PSF). (a) Infrared thermal imaging of the sensor surface under localized heating. A separate focused 532 nm continuous-wave laser (20 × objective, NA = 0.8) was employed as a point-like heat source. The beam was controlled to generate a measured effective excitation spot diameter of 66 μm on the gold grating surface. (b) Cross-sectional thermal intensity profile extracted from the laser-induced hot spot in (a) (yellow cross-section). The spatial resolution of the sensor platform is defined as the full width at half maximum (FWHM) of this thermal distribution. The measured FWHM of 731 μm confirms that the effective spatial resolution is governed by the

lateral thermal diffusion length within the 1-mm-thick quartz substrate, rather than the intrinsic optical limit of the sensing method.

Reply to the Reviewer: We fully agree with the reviewer that the macroscopic chip dimension (2.5 mm × 2.5 mm) represents the substrate size rather than the spatial resolution. To rigorously quantify the effective resolution limit, we performed a thermal Point Spread Function (PSF) characterization using a focused laser-induced micro-heating method. As detailed in **Figure S14**, distinct from the wide-field fluorescence excitation, a separate 532 nm laser was focused to a spot size of 66 μm to act as a localized heat source. The resulting thermal diffusion profile on the sensor surface, imaged via high-precision infrared thermography, reveals a Full Width at Half Maximum (FWHM) of approximately 731 μm. This quantitative result establishes that the current spatial resolution is governed by the lateral thermal diffusion length within the 1-mm-thick quartz substrate, rather than the intrinsic optical limit of the angle-based sensing principle. While the optical readout is capable of resolving the 66 μm excitation spot (as utilized in our Gallium phase transition experiment), the discrimination of independent thermal events on a continuous film is physically bounded by this 731 μm diffusion limit.

Presumably, at least several periods of grating need to be present for a reasonable response (also not discussed in the manuscript).

Reply to the Reviewer: The reviewer's insight concerning the finite array size is theoretically well-founded, as resonance-based devices require a sufficient coherence length. In our high-resolution characterization, the excitation beam was focused to an effective diameter (D_{spot}) of 66 μm. Given the optimized grating period (Λ) of 0.5 μm, the probe beam interacts with approximately 132 grating periods ($N = D_{\mathrm{spot}}/\Lambda \approx 132$). This array size significantly exceeds the empirical threshold established in plasmonic literature. For instance, studies on similar periodic absorbers by Li et al. demonstrate that the optical response saturates and converges to the infinite-array limit

when the number of periods exceeds 30 cycles[4]. Since our experimental condition ($N \approx 132$) is well above this threshold ($N > 30$), the sensor operates in a regime indistinguishable from the bulk periodic structure, ensuring that the angular signals measured at the micro-scale are physically valid and preserve the high Q-factor of the resonance.

Additionally, the thermal conuctivity of SiO_2 is not good.

Reply to the Reviewer: The reviewer correctly points out that the low thermal conductivity of quartz introduces thermal resistance. Our calibration experiments confirmed that this leads to a macroscopic temperature gradient across the 1-mm-thick substrate, which we have corrected using the in-situ Pt RTD calibration protocol described in the response to Point 2. However, a more critical concern is whether a thermal gradient exists microscopically within the sensor's nano-scale functional layers, which would invalidate the effective temperature assumption. To address this, we conducted a Finite Element Method (FEM) thermal simulation replicating the exact sensor geometry under steady-state heating (**Figure S10**). The simulation reveals that the vertical temperature difference ($\Delta T_{\mathrm{micro}}$) between the gold-dielectric interface (where surface plasmon polariton (SPP) coupling occurs) and the top surface of the fluorescent layer is less than 1 mK. This confirms that despite the macroscopic thermal resistance of the substrate, the nano-scale sensing volume remains effectively isothermal, validating the accuracy of the sensing principle ($\Delta T \rightarrow \Delta n$).

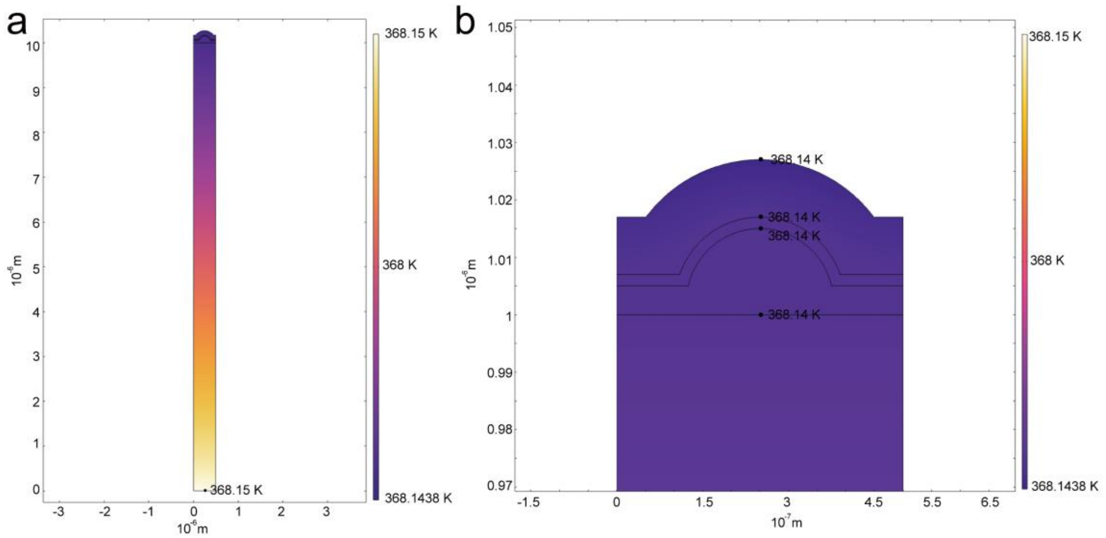

Figure S10. Finite element simulation of the internal thermal distribution within the sensor structure. a) The steady-state temperature field of the full sensor stack modeled using COMSOL Multiphysics under bottom heating at 95 °C. The simulation domain includes the 1-mm-thick quartz substrate and the top functional layers. b) Magnified view of the top functional region from a, illustrating the temperature distribution across the gold grating (150 nm), SiC dielectric (20 nm), and PVA fluorescent layer (100 nm). Quantitative analysis of the vertical temperature profile along the central axis reveals a negligible temperature difference ($\Delta T_{\mathrm{micro}} < 1$ mK) between the top surface of the fluorescence layer and the metal-dielectric interface (368.14 K), confirming that the sensing volume remains thermally uniform and validating the effective temperature assumption used in the sensing principle.

In summary, although the idea of angle-resolved thermometry combing plasmonic structure and temperature response seems interesting, neither the science nor the feasibility was solidly demonstrated in this work.

Reply to the Reviewer: We sincerely appreciate the reviewer's frank assessment, which served as a crucial stress test for our work. While the initial submission may have appeared preliminary, we believe the extensive revisions and supplementary data provided in this resubmission have transformed the manuscript into a scientifically rigorous demonstration. By effectively decoupling the signal from power fluctuations, quantifying the 0.01 °C resolution through phase transition monitoring, and proving

long-term stability over 1000 cycles, we have moved beyond a mere "feasibility study" to validate a robust, high-performance thermometry paradigm. We hope these comprehensive improvements satisfactorily address the reviewer's concerns and demonstrate the substantial scientific value of this angle-based sensing strategy.

Reviewer #2 (Remarks to the Author):

In this work, the authors report a novel approach to thermal sensing using a fluorescence signal. The proposed idea involves combining a gold nanograting with a fluorescent material that is coupled to the grating as a covering film. Throughout the manuscript, the authors claim the existence of a cascade effect that results in a strong temperature dependence of the diffraction angle (superimposed on the emission spectra), which can be used to monitor the temperature of the grating+dye structure.

The field of fluorescence sensing is developing rapidly, especially in applications targeting thermal sensing. Typically, the approach involves the use of a luminescent nanostructure, and only a few alternative methods are reported. This work is novel in that it proposes an alternative way of measuring temperature. In this sense, I believe the paper could be of interest to the scientific community. Nevertheless, I find that the paper presents some issues and drawbacks:

Reply to the Reviewer: We sincerely thank the reviewer for their positive evaluation of the novelty of our work and its potential contribution to the scientific community. We are particularly encouraged by the recognition of our angle-based approach as a distinct alternative to conventional fluorescence thermometry. The reviewer's constructive comments regarding the clarity of the physical mechanism and the practical implementation details have been instrumental in refining the manuscript. In response, we have substantially restructured the theoretical explanation, clarified the experimental procedures, and added rigorous characterizations to fully address the raised issues.

1. After reading several times, the explanation of the "cascade" effect leading to a

diffraction angle strongly dependent on temperature remains unclear to me. I believe the authors should find a clearer way to explain this phenomenon. In the end, it seems that the dominant effect is the change in the refractive index of the fluorescent layer with temperature, which affects the resonance condition, right?

Reply to the Reviewer: We appreciate the reviewer's insightful feedback regarding the clarity of the "cascaded" mechanism and the manuscript's presentation. We acknowledge that the initial explanation of the physical chain was not sufficiently explicit. To address this, we have substantially restructured the theoretical section and redesigned the schematic illustrations.

Regarding the "cascaded" effect explanation: The revised manuscript now presents a rigorous, step-by-step deconstruction of the signal transduction chain. We have completely redesigned **Figure 1** to establish a clear visual hierarchy. **Figure 1a** now explicitly illustrates the macroscopic phenomenon (thermal input → angular shift), while **Figure** 1b details the microscopic "cascaded" chain: Thermal input (ΔT) → Refractive index modulation (Δn) → SPP wavevector modulation (Δk_{SPP}) → Angular shift ($\Delta\theta_{\mathrm{side}}$).To provide a quantitative basis, we mathematically deconstructed the total sensitivity (S_{abs}) in **Figure 4** and the associated text using the chain rule:

$$S_{abs} = \left|\frac{d\theta_{side}}{dT}\right| = \underbrace{\left|\frac{dE_{MLCT}}{dT}\right|}_{\text{Step 1}} \times \underbrace{\left|\frac{dn}{dE_{MLCT}}\right|}_{\text{Step 2}} \times \underbrace{\left|\frac{d\theta_{side}}{dn}\right|}_{\text{Step 3}}$$

This formulation explicitly links the molecular thermochromism (Step 1)[5,6], the resonant refractive index dispersion governed by Kramers-Kronig relations (Step 2)[7,8], and the plasmonic momentum matching (Step 3). The theoretical product of these components (0.678 °/C) shows excellent agreement with our experimental calibration (0.580 °/C), quantitatively validating the proposed mechanism. A detailed theoretical derivation is also provided in Supplementary Note 1.

There are some repeated sentences in the manuscript, and some terms, such as SPP, are not defined (if I am not mistaken).

Reply to the Reviewer: We have conducted a thorough proofreading of the entire manuscript. We ensured that all key terms, such as SPP, are explicitly defined upon their first appearance in the Introduction. Furthermore, we have eliminated redundant phrasing throughout the text to improve readability and flow.

2. It is not clear how the temperature is determined. Is it necessary to measure the entire intensity vs. angle curve for each measurement? How is this done in real experiments?

Reply to the Reviewer: We thank the reviewer for these practical inquiries, which are crucial for assessing the sensor's real-world applicability. regarding the methodology of temperature extraction, the determination is based strictly on identifying the angular position of the secondary diffraction peak (θ_{side}). While the full intensity-versus-angle profile was recorded during the initial characterization to rigorously validate signal integrity, the sensing principle does not necessitate acquiring the entire spectral curve for every measurement. Instead, in our practical monitoring experiments, such as the battery and CPU monitoring presented in **Figure 6**, the protocol was optimized to scan only a narrow angular window (typically $2°–3°$) centered around the peak. The precise angular position (θ_{side}) is then extracted via Gaussian fitting to enable sub-step angular resolution, and this value is directly converted into temperature using the pre-established calibration equation ($T_{\text{actual}} = 80.653 - 1.724 \times \theta_{\text{side}}$).

What is the time required for these measurements? Although the authors report very high sensitivities, in order to compare with other systems, the measurement time must be provided.

Reply to the Reviewer: Regarding the measurement time, it is essential to distinguish between the limits of the current characterization apparatus and the intrinsic speed of the sensing paradigm. In the present configuration, which utilizes a motorized rotation stage for high-precision validation, the data acquisition speed is limited by the mechanical rotation rate ($1°/\text{s}$). Consequently, a typical narrow-window scan requires approximately 2 to 3 seconds, a temporal resolution that was sufficient to capture the thermal dynamics of the battery and CPU monitoring experiments. However, this

mechanical latency is an artifact of the goniometric setup and does not represent the fundamental limit of the sensor. The intrinsic response time is governed only by the thermal equilibration of the micron-scale functional layers, which is rapid (milliseconds scale). For applications requiring high-speed monitoring, we have proposed (see Response to Reviewer 1) that the angle-based paradigm is fully compatible with Fourier plane (back focal plane) imaging. In such a configuration, the angular distribution is mapped instantaneously onto a 2D detector array, eliminating mechanical scanning and allowing the measurement time to be limited only by the detector's frame rate (potentially kHz range), thereby rendering the system comparable to or faster than state-of-the-art thermometry systems.

3. If I am not wrong, the diffraction angle depends on both the temperature of the gold pattern and the luminescent layer. However, these materials have very different thermal conductivities. Is the temperature homogeneous across the structure? If not, how accurate is the thermal output?

Reply to the Reviewer: The reviewer correctly identifies that the sensing signal is governed by the optical properties of the multi-layer stack, where the disparity in thermal conductivities between the metallic grating and the fluorescent layer could theoretically introduce thermal gradients. We recognize that such inhomogeneity would render the effective temperature sensed by the SPP resonance ill-defined. To rigorously quantify the internal temperature distribution, we conducted a FEM thermal simulation under steady-state heating conditions using COMSOL Multiphysics. The model precisely replicated the sensor's geometry, comprising a 1-mm-thick quartz substrate, a 150 nm gold grating layer, a 20 nm SiC dielectric layer, and a 100 nm PVA fluorescent layer. By simulating a worst-case scenario with the substrate bottom fixed at 95 °C and convective cooling applied to the top surface, we evaluated the thermal profile across the functional layers. As illustrated in **Figure** S10, the simulation reveals that while a macroscopic temperature drop occurs across the quartz substrate due to its thermal resistance, the vertical temperature gradient within the nano-scale functional stack is negligible. Specifically, the temperature difference ($\Delta T_{\mathrm{micro}}$) between the gold-

dielectric interface (where SPP coupling occurs) and the top surface of the fluorescent layer is less than 1 mK (< 0.001 °C). This gradient is an order of magnitude smaller than the sensor's resolution limit of 0.01 °C. Consequently, despite the differences in thermal conductivity, the functional sensing volume remains effectively isothermal due to its microscopic thickness, ensuring that the diffraction angle correlates to a single, well-defined temperature across the structure.

4. What spatial resolution can be achieved with this approach?

Reply to the Reviewer: To explicitly define the sensor's applicability limits, we rigorously quantified the effective spatial resolution, which is governed by the lateral thermal diffusion length within the substrate rather than the optical diffraction limit of the sensing principle. We performed a thermal PSF characterization using a laser-induced micro-heating method. Distinct from the wide-field fluorescence excitation, a separate 532 nm continuous-wave laser was focused via a 20 × objective (NA = 0.8) to create a localized heat source with an effective diameter of approximately 66 μm. The resulting steady-state thermal distribution on the 1-mm-thick quartz substrate was imaged using a high-precision cooled infrared thermal imager. As shown in **Figure S14**, the analysis of the thermal spot profile yields a FWHM of approximately 731 μm. This value establishes the physical spatial resolution limit of the current sensor implementation, dictated by the thermal properties of the substrate. While the angle-based optical readout itself is capable of sub-micron resolution, as demonstrated in our micro-spot measurements, the discrimination of independent thermal events on the current continuous film platform is limited to this 731 μm diffusion scale. This resolution is nonetheless significantly superior to standard thermocouple measurements and sufficient for the micro-scale monitoring applications demonstrated in this work.

5. How relevant are external conditions? This question relates not only to the existence of cross-sensitivity (which should be evaluated, for instance, by checking calibration under different environmental conditions such as humidity or the presence of dust) but also to the role played by ambient temperature.

Reply to the Reviewer: The influence of environmental variables on sensor reliability and the precise definition of the measured temperature are indeed critical considerations for practical deployment. We have addressed these interconnected concerns by characterizing the sensor's environmental stability and clarifying the physical meaning of the thermal readout through our calibration protocol. To quantitatively assess cross-sensitivity to environmental conditions, specifically humidity, we conducted a systematic characterization of the angular response across a wide relative humidity (RH) range. As illustrated in **Figure S17**, the sensor exhibits exceptional stability across a broad operational window of 10% to 89% RH, where the fluorescence emission angle remains virtually constant at fixed temperatures. We identified a distinct failure mode only at near-saturation levels ($\geq$ 95% RH), characterized by a sharp decrease in emission angle attributed to the hygroscopic swelling of the PVA matrix, which alters the refractive index and grating period. This characterization confirms the sensor's robustness for operation in typical ambient environments without significant cross-sensitivity drift.

What temperature is actually being measured in their applications? Is it the surface temperature of, for example, batteries, or the temperature of the air in contact with the battery? Note that the latter could be strongly dependent on the thickness of the sensor.

Reply to the Reviewer: Regarding the specific temperature being measured and the potential influence of the sensor's thickness, the rigorous in-situ calibration protocol implemented in this study explicitly addresses the distinction between the target surface temperature and the surrounding ambient air. We recognize that the finite thickness of the sensor introduces thermal resistance, which could lead to a discrepancy between the heat source and the sensing surface. To correct for this, as detailed in our response to Reviewer 1, we employed a co-located platinum resistance thermometer (Pt RTD) directly attached to the sensor surface during calibration. By correlating the angular signal specifically to this actual local surface temperature (T_{actual}) rather than the heat source setpoint (T_{set}), the calibration curve effectively accounts for the thermal gradient across the substrate. Consequently, the reported temperatures in our application

experiments (e.g., the battery casing or CPU surface) represent the precise surface temperature of the monitored object, independent of ambient air temperature variations or the thermal drop across the sensor thickness.

6. Have the authors checked for self-heating effects due to light absorption by gold?

Reply to the Reviewer: The reviewer correctly identifies that the plasmonic resonance mechanism involves optical absorption by the gold grating, which inevitably acts as a localized heat source. Rather than treating this photothermal effect solely as a potential error source, we leveraged the sensor's high sensitivity to quantitatively characterize it via a variable-power excitation experiment. As detailed in **Figure S20**, the results demonstrate a highly linear relationship between the incident laser power and the local temperature rise, yielding a quantified photothermal coefficient of approximately 0.07–0.08 °C/mW. At the standard operating power of ~0.9 mW used for all sensing measurements in this work, this translates to a self-heating induced temperature rise of less than 0.08 °C. Crucially, because the sensor is calibrated in-situ under the exact same illumination conditions, this constant photothermal offset is inherently incorporated into the calibration baseline. Consequently, the self-heating effect does not introduce measurement error during operation, provided the excitation power is stable. Furthermore, the successful quantification of this subtle effect validates the sensor's capability to function as a high-precision micro-calorimeter, capable of resolving minute thermal perturbations induced by optical energy absorption.

Reference

1. Drezet, A. et al. Leakage radiation microscopy of surface plasmon polaritons. *Mater. Sci. Eng. B* **149**, 220–229 (2008).

2. Ohad, A. et al. Spatially resolved measurement of plasmon dispersion using Fourier-plane spectral imaging. *Photonics Res.* **6**, 653–661 (2018).

3. Genco, A. et al. *k*-Space hyperspectral imaging by a birefringent common-path interferometer. *ACS Photonics* **9**, 3563–3572 (2022).

4. Li, Z. et al. Geometry-independent unity absorption in periodic subwavelength

grating. *Opt. Express* **27**, 8375-8384 (2019).

5. Juris A, Balzani V, Barigelletti F, Campagna S, Belser P, Von Zelewsky A. Ru(II) polypyridine complexes: photophysics, photochemistry, electrochemistry, and chemiluminescence. *Coord. Chem. Rev.* **84**, 85-277 (1988).

6. Phan Huu DKA*, et al.* Thermally activated delayed fluorescence: polarity, rigidity, and disorder in condensed phases. *J. Am. Chem. Soc.* **144**, 15211-15222 (2022).

7. Khurgin JB. How to deal with the loss in plasmonics and metamaterials. *Nat. Nanotechnol.* **10**, 2-6 (2015).

8. Lucarini V, Saarinen JJ, Peiponen K-E, Vartiainen EM. Kramers-Kronig relations in optical materials research. *Springer Ser. Opt. Sci.* **110**, 1-161 (2005).

Point-by-Point Response to Editor and Reviewers' Comments

(*Black italic: Reviewer's remarks;* Blue type: Our response)

(Green background: Revised descriptions in main text)

Reviewer #1:

This reviewer acknowledges the substantial expansion of the demonstration of the sensor's reliability, which arises from the comments of both reviewers. The working principle has been solidified by the authors, especially by introducing the Fourier plane imaging capability. It seems solid in theory and might improve the feasibility of the methodology; however, this capability might still remain unexplored at this stage.

Reply to the Reviewer: We express our sincere gratitude to the reviewer for acknowledging the solidity of the working principle and the theoretical feasibility of the proposed Fourier plane imaging capability. We agree with the astute observation of the reviewer that while this capability is solidly grounded in theory, its specific experimental implementation remains an unexplored avenue in the current manuscript. We would like to clarify that the primary objective of this work is to establish the fundamental physical mechanism of the sensor which specifically involves the high sensitivity arising from the interplay between the thermal modulation of the refractive index of the polymer matrix and the temperature-dependent spectral shift of the fluorophore. For this fundamental purpose, the current high-precision goniometric setup serves as the established benchmark for characterization. It offers superior angular resolution and spectral purity allowing us to quantitatively validate the sensitivity hierarchy and distinguish the 0.58 °/°C shift with minimal optical aberration. We emphasize that the Fourier plane imaging scheme and the current rotational scanning scheme are physically equivalent as both map the same SPP momentum matching condition. These methods convert the angular information into either a spatial distribution in Fourier imaging or a temporal sequence in rotational scanning. Therefore, the rigorous experimental validation of the sensing mechanism provided in this work lays the necessary physical foundation for the future engineering realization of the

Fourier plane sensor. We fully envision Back Focal Plane imaging as the immediate next step to transition this technology from fundamental research to high-speed static industrial monitoring applications.

Further questions regarding the working molecules: 1. currently, Ru(phen)3Cl2 molecules are used. It is known for oxygen quenching. A PVA polymer film might be sufficient for resistance to oxygen quenching, but further experiments might need to be carried out to confirm this.

Reply to the Reviewer: To rigorously evaluate the resistance of the Ru(phen)$_3$Cl$_2$-doped PVA sensing layer to oxygen quenching, we further performed fluorescence stability tests across a wide pressure range spanning from a near-vacuum state of 3.8 Pa to ambient atmospheric pressure of 100 kPa (Supplementary Information, Figure S19). We adopted a strict stabilization protocol at each pressure node maintaining the chamber state for at least five minutes to ensure thermodynamic equilibrium before data acquisition. The experimental results reveal a complex pressure-dependent response that reflects the gas permeation dynamics in thin polymer films.

As the environmental pressure increased from 3.8 Pa to 100 kPa, the normalized fluorescence intensity exhibited a segmented attenuation pattern rather than a simple linear decay. Specifically, the intensity remained high at approximately 0.996 in the high-vacuum region of 3.8 Pa but experienced a discernible initial decrease to 0.974 as the pressure rose slightly to 5 Pa. Subsequently, a gradual decline was observed across the intermediate pressure range where the signal dropped from 0.965 at 10 kPa to 0.921 at 58 kPa. Finally, as the pressure approached atmospheric levels (100 kPa), the signal stabilized at a lower baseline of approximately 0.900. This saturation behavior indicates that while the external pressure facilitates oxygen permeation, the extent of quenching is fundamentally limited. The observed total attenuation of approximately 10% confirms that the dense hydrogen-bonded network of the high-molecular-weight PVA matrix provides effective gas barrier properties[1, 2]. However, the 100 nm thickness allows for a minor degree of oxygen infiltration under high pressure which triggers a residual dynamic quenching effect consistent with the Stern-Volmer relationship

observed in ruthenium-based sensor systems[3].

However, this intensity fluctuation serves to highlight the distinct advantage of our proposed methodology. In stark contrast to the 10% drift observed in the intensity signal, the emission angle serving as the core sensing parameter of our approach demonstrated exceptional robustness throughout the entire pressure loading process. The measured emission angle remained remarkably stable fluctuating strictly between 32.65° and 32.62° with a standard deviation as low as 0.003°. This result confirms that the angular resonance condition is governed by the effective refractive index of the waveguide which is virtually immune to the presence of trace oxygen molecules that significantly impact fluorescence quantum yield. Consequently, the angle-based demodulation scheme effectively isolates the temperature measurement from environmental variables such as pressure fluctuations and oxygen concentration thereby offering superior reliability over intensity-based methods which would suffer significant errors under identical conditions.

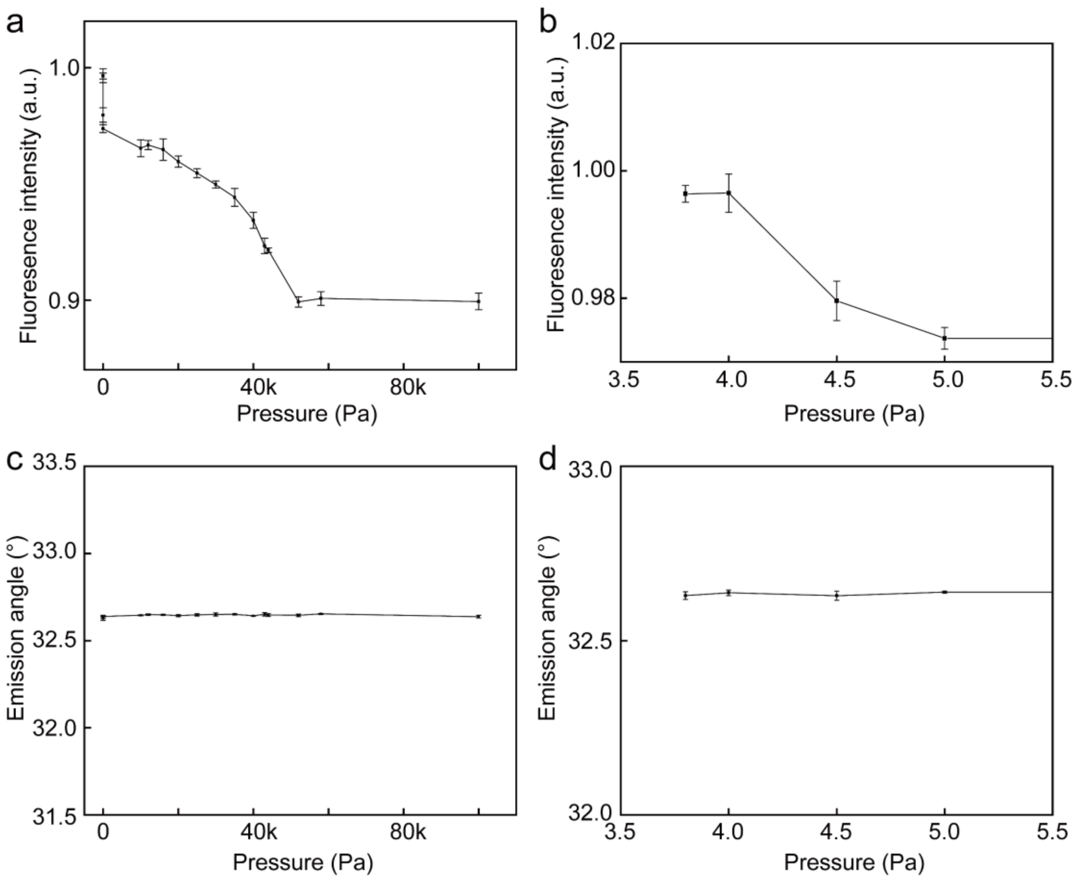

Figure S19. Fluorescence intensity and emission angle response to environmental

pressure variations. a, b) Normalized fluorescence intensity of the $[Ru(phen)_3]Cl_2$ sensing layer recorded as a function of environmental air pressure at a constant temperature of 25 °C. a) Displays the full pressure range spanning from relatively high vacuum (3.8 Pa) to ambient atmospheric pressure (100 kPa), while b) provides a magnified view of the low-pressure region between 3.5 Pa and 5.5 Pa. The fluorescence intensity exhibits a segmented attenuation of approximately 10% as the pressure increases to atmospheric levels, attributed to oxygen quenching. c, d) Corresponding fluorescence emission angle measured simultaneously across the identical pressure range, with d) highlighting the magnified low-pressure regime. The emission angle varies between 32.62° and 32.65° throughout the pressure loading process, yielding a standard deviation of 0.003°.

2. Does this methodology apply to all MLCT fluorophores, or all fluorophores that are stable as long as they are in the designed wavelength (and coupled to a certain wavelength of the plasmon structure)?

Reply to the Reviewer: The methodology established in this study is theoretically applicable to a broad spectrum of fluorophores beyond MLCT complexes, including lanthanides, quantum dots, and organic dyes, provided they exhibit stable radiative transitions within the spectral overlap region of the plasmonic structure. As derived from the grating coupling derivation in Equations 2 and 3, the sensing signal is fundamentally driven by the thermal modulation of the effective refractive index of the sensing layer rather than solely by luminescence quenching. Consequently, any fluorophore-matrix system possessing a temperature-dependent refractive index can function effectively within this framework.

In the specific case of MLCT fluorophores such as $[Ru(phen)_3]Cl_2$, the system benefits from a synergistic mechanism where temperature-dependent spectral shift introduces an additional refractive index modulation via Kramers-Kronig relations. This resonant dispersion effect acts in the same direction as the thermal expansion of the matrix, thereby amplifying the angular sensitivity. Conversely, for fluorophores that are spectrally stable against temperature variations, such as lanthanide complexes or

quantum dots encapsulated in polymer matrices[4], the sensing mechanism operates primarily through the thermal modulation of the refractive index of the host matrix. While the sensitivity magnitude in these wavelength-stable systems might be lower compared to MLCT-enhanced configurations due to the absence of the resonant dispersion contribution, they remain viable candidates for detection. These narrow-band emitters offer distinct advantages such as sharper diffraction peaks and higher spectral resolution which are critical for high-precision detection scenarios. Thus, the choice of fluorophore allows for a tunable trade-off between sensitivity and resolution within the same sensing architecture.

If the fluorophore coating is modified, or another material is applied (which has a similar structure or luminescence), how would the response curve be changed?

Reply to the Reviewer: The experimental sensitivity hierarchy observed across the three configurations, namely 0.58 °/°C for PVA/[Ru(phen)$_3$]Cl$_2$, 0.48 °/°C for PVA/Rhodamine B (RhB), and 0.35 °/°C for PMMA/[Ru(phen)$_3$]Cl$_2$, provides a quantitative basis for decoupling the contributions of the polymer matrix and the fluorophore to the overall angular shift (Supplementary Information, Figure S12). By differentiating the grating coupling equation with respect to temperature, the angular sensitivity is determined by the superposition of the thermo-optic coefficient of the matrix (dn/dT) and the temperature-dependent wavelength shift of the fluorophore ($d\lambda/dT$).

A comparison between the two PVA-based systems isolates the specific contribution of the spectral redshift. Since the PVA matrix is identical in both cases, the baseline refractive index modulation remains constant. The higher sensitivity observed in the [Ru(phen)$_3$]Cl$_2$ system relative to the RhB counterpart is attributed to the distinct photophysical responses of the probes. Specifically, the MLCT emission of Ru(phen)$_3$ is sensitive to the micro-environment. As the temperature increases, thermally activated relaxation leads to a measurable bathochromic shift of the emission wavelength[5, 6]. This spectral redshift acts in the same direction as the refractive index reduction, amplifying the total angular displacement. In contrast, the emission of

Rhodamine B arises from $\pi - \pi^*$ electronic transitions of the xanthene core[7]. While RhB exhibits thermal quenching, established thermometry studies indicate that its response is dominated by quantum yield variations with minimal spectral shift in this temperature range[8]. Consequently, the RhB sensor response is driven primarily by the matrix refractive index change without the additional spectral enhancement factor present in the Ru system.

Furthermore, the comparison between the PMMA/[Ru(phen)$_3$]Cl$_2$ and the PVA-based systems highlights the dominant role of the matrix properties. Despite employing the same wavelength-shifting [Ru(phen)$_3$]Cl$_2$ probe, the PMMA system exhibits a sensitivity of 0.35 °/°C, which is lower than that of the wavelength-stable PVA/ RhB system. This observation indicates that the temperature-dependent refractive index change of the PMMA matrix is lower than that of PVA in the tested range. According to the free volume theory, the higher glass transition temperature (T_g) and cross-linked rigidity of the PMMA network restrict the segmental mobility and thermal expansion compared to the PVA matrix[9, 10]. Consequently, the refractive index modulation of the matrix serves as the primary driver for detection sensitivity.

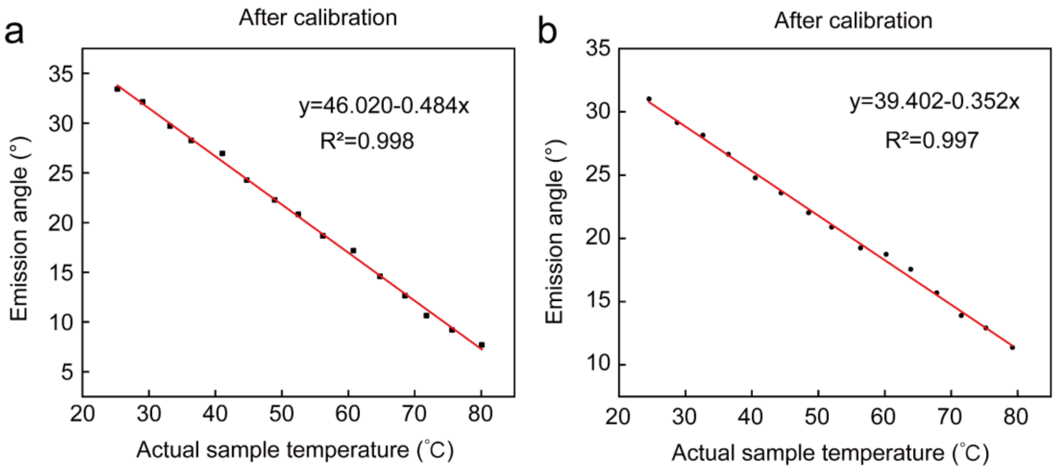

Figure S12. Comparative thermal sensitivity analysis using alternative fluorophore and matrix configurations. a) Temperature dependence of the fluorescence emission angle for the PVA matrix doped with Rhodamine B. The measured sensitivity is 0.48 °/°C. b) Temperature dependence of the emission angle for the PMMA matrix doped with [Ru(phen)$_3$]Cl$_2$. The measured sensitivity is 0.35 °/°C.

The methodology involving moving parts: It is still confusing regarding the mention of rotating turbine blades, high-speed hard disk platters, or centrifuge components. How is this going to work? How is the system mechanically stable if a grating is attached?

Reply to the Reviewer: We sincerely thank the reviewer for highlighting the practical engineering challenges associated with the applications involving high-speed moving parts. We wish to clarify that the specific examples of rotating turbine blades and hard disk platters were discussed in our previous response letter to illustrate potential scanning modalities but were not included in the main text of the manuscript. We fully concur with the reviewer that the mechanical integration of gratings onto such rapidly rotating components presents complex stability issues that are beyond the scope of the current work. Consequently, we have ensured that the outlook section of the manuscript remains strictly grounded in our experimental findings. Instead of speculative industrial scenarios, the discussion focuses on the demonstrated capabilities of the sensor as a high-precision physical detection platform. Drawing from the successful validation in monitoring the phase transition of gallium and quantifying laser-induced thermal effects, we emphasize the potential of this methodology for the fundamental investigation of thermodynamic processes such as the real-time characterization of phase-change materials and the analysis of heat dissipation dynamics in micro-optomechanical systems where high thermal resolution and non-contact operation are critical.

From Figure S17, the current emission probe decomposed at around 100 degrees Celsius, which suggests that the authors should consider the temperature range as well.

Reply to the Reviewer: We appreciate the reviewer's careful examination of the sensor's operational range. We would like to clarify the interpretation of the data presented in Figure S18 (updated from Figure S17) which characterizes the sensor's stability under varying relative humidity at constant temperatures rather than a continuous temperature sweep. The signal variations observed in Figure S18 at near-saturation humidity levels arise from the hygroscopic swelling of the hydrophilic matrix which alters the grating period and effective refractive index physically.

However, the reviewer is correct that the operational temperature range is a critical consideration. Regarding the upper limit, as shown in Table S1, the maximum calibrated temperature in this study is 79.36 °C. This boundary is not due to the decomposition of the emission probe, but is a conservative design choice dictated by the T_g of the PVA matrix. In this study, we employed a high-molecular-weight PVA (Mw ≈ 190000, >98% hydrolyzed). According to polymer physics principles, the extensive intermolecular hydrogen bonding network formed in fully hydrolyzed PVA chains restricts segmental motion, resulting in a glass transition temperature typically in the vicinity of 85 °C[11]. As the operating temperature approaches this thermodynamic critical point, the enhanced segmental mobility of the polymer chains leads to a reduction in the matrix rigidity which can induce nonlinear optical responses and restrict the calibration linearity. Conversely, the $[Ru(phen)_3]Cl_2$ fluorophore itself possesses thermal stability well beyond the current testing range. Thermogravimetric analyses of such ruthenium polypyridine complexes confirm that their onset decomposition temperature generally exceeds 270 °C which ensures their chemical integrity within the reported sensor operating window[12]. Therefore, the current temperature range is a conservative design choice based on the viscoelastic properties of the PVA matrix. We emphasize that this limitation is material-specific rather than intrinsic to the sensing methodology. Future investigations could explore substituting the PVA matrix with high-performance engineering polymers, such as polyimide, which retains mechanical stability up to 300 °C[13]. This material substitution strategy presents a promising avenue to potentially extend the operational ceiling of the angle-based sensing architecture for higher-temperature requirements.

Regarding the spatial resolution (Comment 3), the current demonstration could be potentially misleading. In thermometry, the physical dimension of the sensor is critical because a thermal equilibrium must be established between the measured object and the thermometer. This is precisely why nanothermometers are generally preferred for high-accuracy measurements at small scales, as they minimize thermal perturbation. We acknowledge that the authors carried out rigorous in-situ temperature calibration

(Figure S9) to account for the thermal resistance of the materials used. However, a fundamental limitation remains: if the target object has a smaller heat capacity than the micro-sized grating sensor, the sensor will act as a heat sink. Consequently, the measured value will represent the equilibrium temperature between the object and the sensor, rather than the intrinsic temperature of the object. Moreover, establishing thermal equilibrium also requires time, which will inevitably impact the response time of the thermometer. This limitation should be explicitly stated in the maintext to prevent any misinterpretation of the achieved spatial resolution.

Reply to the Reviewer: We express our sincere gratitude to the reviewer for the insightful analysis regarding the physical constraints of contact thermometry. We fully agree with the scientific assessment that the establishment of thermal equilibrium dictates the measurement accuracy particularly when the sensor heat capacity exceeds that of the target object. In such scenarios the sensor indeed functions as a heat sink causing the measured value to deviate from the intrinsic temperature of the target. To address this critical point and prevent potential misinterpretation we have revised the manuscript to explicitly articulate this fundamental limitation. The updated discussion on spatial resolution clarifies that the measured full width at half maximum of 731 micrometers reflects the effective thermal spreading length on the substrate rather than the optical diffraction limit. Furthermore, we explicitly acknowledged in the revised text that the thermal equilibration process imposes inherent constraints on both the measurement accuracy for low-thermal-mass objects and the temporal response speed of the sensor.

Reference

1. Mallamaci G, Al Faysal A, Guinault A, et al. Innovative poly(vinyl alcohol)-based nanolayered films: balancing mechanical and gas barrier properties. *ACS Appl. Mater. Interfaces* **17**, 36182–36191 (2025).

2. Charoensri K, Kwon D H, Kim H S, Hongrattanavichit I, Shin Y J, Park H J. Process-

controlled functional polymer films on paper: oxygen barrier and antimicrobial performance of PVA–amylose coatings. *Polymers* **18**(2), 264 (2026).

3. Zhao Y, Zhang H, Jin Q, Jia D, Liu T. Ratiometric optical fiber dissolved oxygen sensor based on fluorescence quenching principle. *Sensors* **22**(13), 4811 (2022).

4. Wang X D, Wolfbeis O S, Meier R J. Luminescent probes and sensors for temperature. *Chem. Soc. Rev.* **42**, 7834–7869 (2013).

5. N. Bustamante, et al. optimization of temperature sensing with polymer-embedded luminescent Ru(II) complexes. *Polymers* **10**(3), 234 (2018).

6. D. Jacquemin, et al. thermal equilibration between excited states or solvent effects: unveiling the origins of anomalous emissions in heteroleptic Ru(II) complexes. *Phys. Chem. Chem. Phys.* **20**, 11559–11563 (2018).

7. Lakowicz, J. R. *Principles of Fluorescence Spectroscopy* 3rd edn (Springer, New York, 2006).

8. Ross, D., Gaitan, M., Locascio, L. E. Temperature measurement in microfluidic systems using a temperature-dependent fluorescent dye. *Anal. Chem.* **73**, 4117–4123 (2001).

9. C. E. Porter, F. D. Blum. thermal characterization of PMMA thin films using modulated differential scanning calorimetry. *Macromolecules* **33**(19), 7016–7020 (2000).

10.P. N. Samanta, D. Majumdar, J. Leszczynski. thermophysical and mechanical responses of poly(vinyl alcohol) from molecular simulations. *Phys. Chem. Chem. Phys.* **27**, 9448–9462 (2025).

11. Couți N, et al. Polyvinyl alcohol, a versatile excipient for pharmaceutical 3D printing. *Polymers* **16**, 517 (2024).

12. Motaung, M. P., Ajibade, P. A., Le Roux, L. J. Synthesis and photophysical studies of Ru(II) and Co(II) complexes of bis(benzimidazolyl)pyridine and evaluation of their potential as sensitizers for dyes sensitized solar cells. *Int. J. Electrochem. Sci.* **11**, 10953–10964 (2016).

13. Liaw, D.-J. et al. Advanced polyimide materials: Syntheses, physical properties and applications. *Prog. Polym. Sci.* **37**, 907–974 (2012).
